# Unified master equation for molecules in phonon and radiation baths

**DOI:** 10.1038/s41598-022-22732-w

**Published:** 2022-11-21

**Authors:** C. H. Raymond Ooi, K. J. Cedric Chia

**Affiliations:** Department of Physics, University of Malaya, 50603 Kuala Lumpur, Malaysia

**Keywords:** Optics and photonics, Physics

## Abstract

We have developed a unified quantum optical master equation that includes the dissipative mechanisms of an impurity molecule in crystals. Our theory applies generally to polyatomic molecules where several vibrational modes give rise to intramolecular vibrational redistributions. The usual assumption on identical shapes of the nuclear potentials in ground and excited electronic states and the rotating wave approximation have been relaxed, i.e. the vibrational coordinates are different in the ground and excited states, with counter-rotating terms included for generality. Linear vibrational coupling to the lattice phonons accounts for dissipations via non-radiative transitions. The interaction of a molecule with photons includes Herzberg–Teller coupling as the first order non-Condon interaction where the transition dipole matrix elements depend linearly on vibrational coordinates. We obtain new cross terms as the result of mixing the terms from the zeroth-order (Condon) and first-order (non-Condon) approximations. The corresponding Lamb shifts for all Liouvilleans are derived explicitly including the contributions of counter-rotating terms. The computed absorption and emission spectra for carbon monoxide is in good agreement with experimental data. We use our unified model to obtain the spectra for nitrogen dioxide, demonstrating the capability of our theory to incorporate all typical dissipative relaxation and decoherence mechanisms for polyatomic molecules. The molecular quantum master equation is a promising theory for studying molecular quantum memory.

## Introduction

Amongst many quantum technology applications, quantum memories are at the heart of quantum networks. Quantum repeater^1,2^ solves the issue of the imperfection transmission channels for long distance quantum communication thus making quantum key distribution possible for arbitrarily long distances. In recent years and there have been active research on the design of different quantum information protocols^2–4^. There has been great interest in using molecules as a platform for quantum information processing. Molecule has many degrees of freedom and can be the promising candidate for the development of many applications in quantum information processing. It allows us to store and retrieve larger amount of quantum bits simultaneously such that probabilistic events can by synchronized and operations can be timed appropriately. The power of synthetic chemistry provides the ability to manipulate and modify molecules, this means chemical systems offer advantages that are unavailable to solid state systems, for example rare-earth ion-doped crystals^5,6^ and diamond color centers^7,8^. Some other key advantages besides the ease of fabrication for scalability, organic molecules can have strong zero-phonon lines (ZPL), providing bright and stable sources of photons that is competitive with solid-state devices^9^.

Before molecule-based quantum memories could be realized, one needs to have a good understanding on the relaxation and decoherence channels associated with the molecular system. Both radiation and radiationless transitions must be considered on top of other collision-induced dephasing processes that can occur in the condense phase. For polyatomic molecule, uni-molecular process such as intramolecular vibrational energy redistribution (IVR) can also impact the dynamics in a profound way^10^.

In a recent work^11^, a two-level electronic states (TLS) coupled to localized molecular vibration (LMV) was used to model a single dibenzoterrylene (DBT) molecule. The shapes of the ground and excited electronic levels of interest are assumed to be identical except their minima are shifted with respect to each other. The interaction between DBT and its environment (anthracene nano-crystal) is described by a linear and quadratic coupling term between phonons and TLS. They further showed that the quadratic term is a consequence of anharmonicity of the thermal phonon modes and it is crucial for capturing the temperature dependent homogeneous broadening of the ZPL in the emission spectra. Here we show that by introducing two sets of bosonic operators for the ground and excited potential energy surface (PES) coordinates, we do not obtain the quadratic term and only need to focus on linear coupling terms that we derived. Coupling to the external electromagnetic fields (EM) is also considered in^11^ and the system is solved using the polaron transformation to obtain polaron master equation^12,13^. This polaron transformation approach has gained many attention lately because of its capability to include non-Markovian effect of the thermal phonon bath, some recent applications of this approach can be found in^14–18^.

Most of the works found in the literature have assumed that the two nuclear potentials in the ground and excited electronic levels are only displaced but having identical curvature (i.e. same vibrational frequency). This model is commonly referred to as “displaced harmonic oscillator” in the literature and is closely related to the Holstein–Hamiltonian^19,20^ that employs second quantization to obtain a linear coupling term between the electronic operators and the vibrational operators. However, in reality the nuclear potentials are not only displaced but also having different curvature which is the case for the polyatomic molecules. Even though the Franck–Condon factor^21^ between harmonic oscillators of different shape has been investigated^22,23^, to the best of our knowledge, this has not been considered in the context of an open quantum system framework for modelling realistic molecule.

In this work, we present a unified model of a typical polyatomic molecule interacting with the phonon and radiation baths or environmental reservoir. We consider the vibrational potential of the excited electronic state and ground state are different in vibrational frequency, each has more than one mode (multi-vibrational modes).

A molecule is a system with more than one degrees of freedom in contrast to an atom, a common approach to deal with the complexity of a multipartite system coupling to the reservoir is to assume that each part of the system interacts independently with its own reservoir. For example in the study of vibrational relaxation of molecules in condensed phase, it is assumed that different vibrational modes of the molecule dissipates independently through their own reservoir and they do not interact with each other^24,25^. This approximation has received attention recently and it was shown that it does not capture the correct dynamics of the system^26–28^. Therefore, here we relax this approximation in our model and we will see the consequence of this is that one can obtain additional terms what we hereby referred to as the *“cross term Liouvillean”*. These terms arise naturally from the derivation of the master equation when we allow different parts of the system to couple to the same reservoir. Compared to multimode Brownian oscillator model (MBO) model of Yan and Mukamel^29–31^, and Tanimura^32^ where theory are more interested in the transient dynamics, the novelty of this work is that it is a unified theory for the dissipation mechanisms as it was not considered before in any other related works to the best of our knowledge.

**Figure 1:**
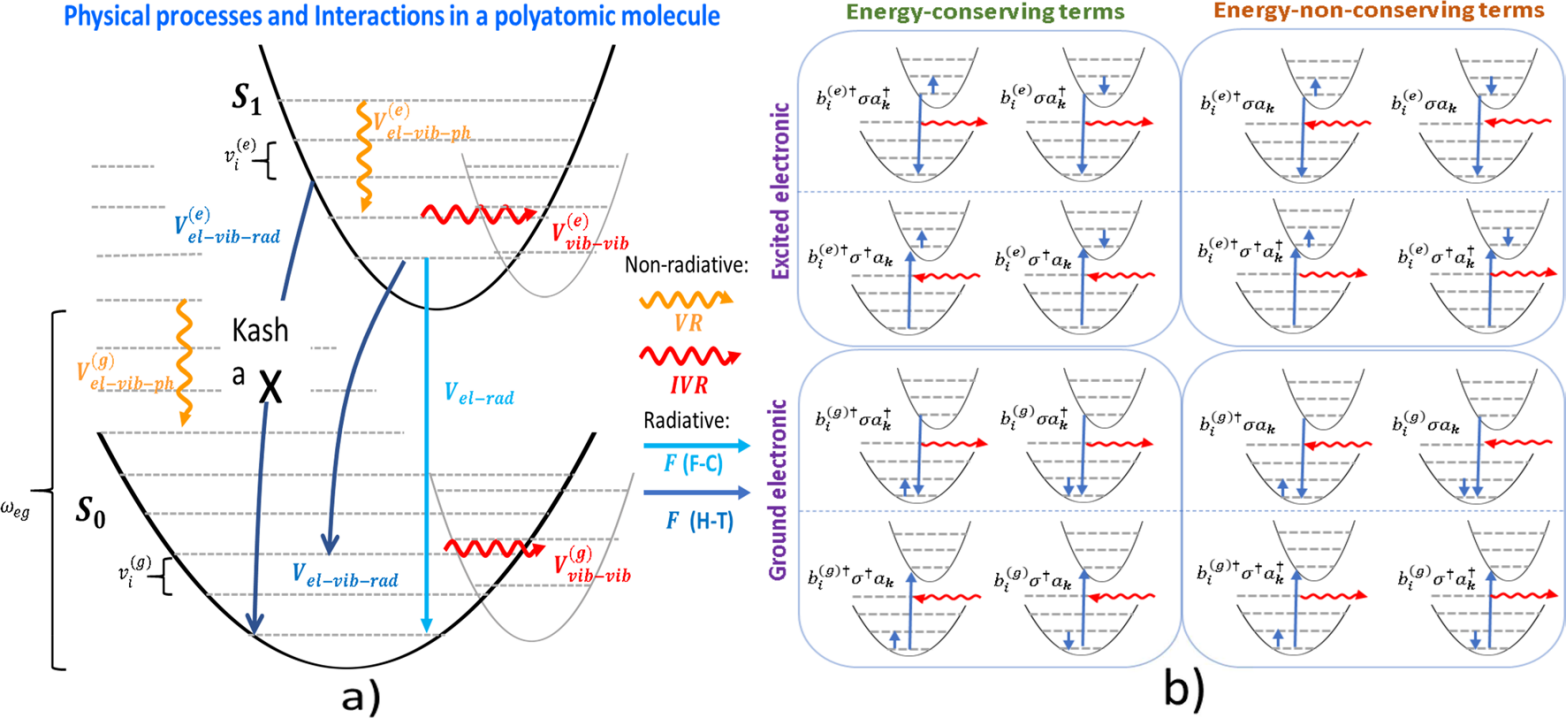
(**a**) Open quantum system model of an impurity molecule in phonon and radiation baths. The system (molecule) contains two electronic states, the ground singlet state $S_{0}$ and excited singlet state $S_{1}$ with energy gap $\omega _{eg}=\omega _{e}-\omega _{g}$ and localized vibrational modes with frequency $v_{i}^{\left( g\right) }$($v_{i}^{\left( e\right) }$) for the ground (excited) state. The localized vibrational modes of the molecule are described by a set of harmonic oscillators. The vibrational frequencies are shifted to $\tilde{v} _{i}^{\left( g\right) }$($\tilde{v}_{i}^{\left( e\right) }$) as a consequence of coupling to the phonon bath and internal coupling within the system. The VR process from interactions with phonon bath manifest the Kasha rule which states that the vibrational relaxation in the excited state is typically very fast such that fluorescence only occur from the ground vibrational levels of the excited state. (**b**) A schematic diagram of different interactions that can take place in the Herzberg–Teller interaction term. The left panels are energy-preserving terms and the right panels are energy non-preserving terms usually neglected under RWA. The processes in the excited and ground electronic states are distinguished.

In “The model” section, we introduce our model for the molecules that consists of two electronic states, each with vibrational sublevels (see Fig. 1 ). The coupling terms to the phonon thermal bath and radiation field derived in this section is where the *Condon-approximation has been relaxed* which resulting in a nuclear coordinates-dependent transition dipole matrix elements. The dynamics of the reduced system (molecule) is investigated in “Dynamics of the quantum system” section using master equation. We *do not make the usual rotating wave * approximation to investigate the different time scales of our system. “Two-time correlation functions” section is devoted to the absorption and emission spectra which will be simulated numerically using Python package QuTiP^33^ using all the microscopic parameters in the theory. For simplicity we consider diatomic molecules, such as carbon monoxide (CO). The discussions of the results in “Results and discussion” section include comparison with the absorption spectrum obtained from experimental data confirm our theory. We also simulate the spectra of nitrogen dioxide and discuss the dependence on various damping mechanisms and parameters such as phonon cutoff frequency.

## The model

In this section, we develop a general theoretical description of a single impurity molecule in phonon reservoir. The theory may be adapted to f-centers in ionic crystals. The model is illustrated using Fig. 1, two electronic states are considered: ground ($S_{0}$) and excited singlet state ($S_{1}$) which we labelled as $\left| g\right\rangle$ with energy $\hbar \omega _{g}$ and $\left| e\right\rangle$ with energy $\hbar \omega _{g}$ respectively. The black parabolas represent potential energy surfaces (PES) in the ground and excited states, assumed to be harmonic but having different frequencies which we labelled as $v_{i}^{(g)}$ and $v_{i}^{(e)}$, respectively. The grey parabolas represent other vibrational modes that can be found in polyatomic molecule and the index *i* denote different vibrational modes. The frequency difference between ground vibrational levels in the ground and excited state is $\omega _{eg}+\frac{1}{2}(v_{i}^{(e)}-v_{i}^{(g)})$ (that gives the zero-phonon line (ZPL)), with $\omega _{eg}=\omega _{e}-\omega _{g}$. The ground and excited PES are shifted with respect to each other because the electronic configuration that determines the molecular bond strength of the excited state is different in general from the ground state. The straight and wavy arrows in Fig. 1 represent radiative and non-radiative transitions respectively due to coupling with the phonon and radiation reservoirs. The physical origin of these interaction terms are summarized in caption of Fig. 1. The following section is devoted to deriving these interaction terms by considering their physical origin.

### Molecular–phonon interaction

We introduce two set of coordinates $x_{i}^{\left( g\right) }$ and $x_{i}^{\left( e\right) }$ for the *i*-th vibrational mode of the ground and excited PES respectively. Since the excited PES is shifted with respect to the ground PES, the two coordinates are related via $x_{i}^{\left( e\right) }=x_{i}^{\left( g\right) }-s_{i}$ where $s_{i}$ is the displacement between the ground and excited PES of *i*-th vibrational mode. The Hamiltonian describing the molecular system and the phonon bath can thus be written as

1 $$\begin{aligned} H_{\mathrm {mol-ph}}=\sigma _{gg}\left( \hbar \omega _{g}+U_{g}\right) +\sigma _{ee}\left( \hbar \omega _{e}+U_{e}\right) \end{aligned}$$

where $\sigma _{gg}=\left| g\right\rangle \left\langle g\right| =\sigma \sigma ^{\dagger }$ and $\sigma _{ee}=\left| e\right\rangle \left\langle e\right| =\sigma ^{\dagger }\sigma$, with Pauli raising ($\sigma ^{\dagger }=\left| e\right\rangle \left\langle g\right|$) and lowering ($\sigma =\left| g\right\rangle \left\langle e\right|$) operators. $U_{g}$ and $U_{e}$ in Eq. (1) denote the vibrational and phonon (*ph*) energies in the ground and excited states, respectively, defined as

2 $$\begin{aligned} U_{\alpha } &=  H_{\alpha }+U_{\alpha }^{\left( ph\right) } \nonumber \\ &=  \sum _{i}\left( \frac{p_{i}^{\left( \alpha \right) 2}}{2m_{i}}+\frac{1}{2} m_{i}v_{i}^{\left( \alpha \right) 2}x_{i}^{\left( \alpha \right) 2}\right) +\sum _{\mathbf {l}}\left( \frac{p_{\mathbf {l}}^{2}}{2m_{\mathbf {l}}}+\frac{1}{ 2}m_{\mathbf {l}}\omega _{\mathbf {l}}^{2}(q_{\mathbf {l}}-\sum _{i}x_{i}^{ \left( \alpha \right) })^{2}\right) \end{aligned}$$

with $\alpha =\left\{ e,g\right\}$. The first summation describes the approximated harmonic nuclear PES with vibrational momentum $p_{i}^{\left( \alpha \right) }$, displacement $x_{i}^{\left( \alpha \right) }$, mass $m_{i}$ and frequency $v_{i}^{\left( \alpha \right) }$ for the *i*-th vibrational mode of the $\alpha$ electronic state. The second summation described the dissipative coupling of the nuclear vibrational motion to phonon bath through the term $q_{\mathbf {l}}-\sum _{i}x_{i}^{\left( \alpha \right) }$, with the index $\mathbf {l}$ labelling different phononic modes; $p_{\mathbf {l}}$ , $q_{\mathbf {l}}$, $m_{\mathbf {l}}$ and $\omega _{\mathbf {l} }$ the phonon momentum, displacement, mass and frequency.

We may decompose Eq. (1) as $~H_{\mathrm {mol-ph}}=H_{ \mathrm {el}}+H_{\mathrm {vib}}+H_{\mathrm {ph}}+V_{\mathrm {el-vib-ph}}+V_{ \mathrm {vib-vib}}$ with

3 $$\begin{aligned} H_{\mathrm {el}}&= {} \hbar \omega _{g}\sigma _{gg}+\hbar \omega _{e}\sigma _{ee}\text {,} \end{aligned}$$

4 $$\begin{aligned} H_{\mathrm {vib}}&= {} \sum _{\alpha }H_{\mathrm {vib}}^{\left( \alpha \right) }=\sum _{\alpha }\sigma _{\alpha \alpha }\sum _{i}\left( \frac{p_{i}^{\left( \alpha \right) 2}}{2m_{i}}+\frac{1}{2}m_{i}\tilde{v}_{i}^{\left( \alpha \right) 2}x_{i}^{\left( \alpha \right) 2}\right) =\sum _{\alpha }\sigma _{\alpha \alpha }\sum _{i}\hbar \tilde{v}_{i}^{\left( \alpha \right) }\left( b_{i}^{\left( \alpha \right) \dagger }b_{i}^{\left( \alpha \right) }+\frac{1 }{2}\right) \text {,} \end{aligned}$$

5 $$\begin{aligned} H_{\mathrm {ph}}&= {} \sum _{\mathbf {l}}\left( \frac{p_{\mathbf {l}}^{2}}{2m_{ \mathbf {l}}}+\frac{1}{2}m_{\mathbf {l}}\omega _{\mathbf {l}}^{2}q_{\mathbf {l} }{}^{2}\right) =\sum _{\mathbf {l}}\hbar \omega _{\mathbf {l}}\left( d_{\mathbf { l}}^{\dagger }d_{\mathbf {l}}+\frac{1}{2}\right) \end{aligned}$$

where the vibrational frequency is shifted $\tilde{v}_{i}^{\left( \alpha \right) 2}=v_{i}^{\left( \alpha \right) 2}+\frac{2W}{m_{i}}$ with $W=\frac{1 }{2}\sum _{\mathbf {l}}m_{\mathbf {l}}\omega _{\mathbf {l}}^{2}$ due to the phonon bath. In deriving these results, we have introduced two set of bosonic operators, for the molecular vibrational coordinates:

6 $$\begin{aligned} x_{i}^{\left( \alpha \right) }=\sqrt{\frac{\hbar }{2m_{i}\tilde{v} _{i}^{\left( \alpha \right) }}}\left( b_{i}^{\left( \alpha \right) \dagger }+b_{i}^{\left( \alpha \right) }\right) \text {, }p_{i}^{\left( \alpha \right) }=i\sqrt{\frac{\hbar m_{i}\tilde{v}_{i}^{\left( \alpha \right) }}{2}} \left( b_{i}^{\left( \alpha \right) \dagger }-b_{i}^{\left( \alpha \right) }\right) \end{aligned}$$

and for oscillators in phonon bath:

7 $$\begin{aligned} q_{\mathbf {l}}=\sqrt{\frac{\hbar }{2m_{\mathbf {l}}\omega _{\mathbf {l}}}} \left( d_{\mathbf {l}}^{\dagger }+d_{\mathbf {l}}\right) \text {, }p_{\mathbf {l} }=i\sqrt{\frac{\hbar m_{\mathbf {l}}\omega _{\mathbf {l}}}{2}}\left( d_{ \mathbf {l}}^{\dagger }-d_{\mathbf {l}}\right) \text {.} \end{aligned}$$

Coupling between the vibrational levels of $\alpha$ electronic state with the phonon bath leads to non-radiative (NR) vibrational relaxation (VR) governed by $V_{\mathrm {el-vib-ph}}=\sum _{\alpha }V_{\mathrm {el-vib-ph} }^{\left( \alpha \right) }$,

8 $$\begin{aligned} V_{\mathrm {el-vib-ph}}^{\left( \alpha \right) }&= {} -\sigma _{\alpha \alpha }\sum _{i}x_{i}^{\left( \alpha \right) }\sum _{\mathbf {l}}m_{\mathbf {l}}\omega _{\mathbf {l}}^{2}q_{\mathbf {l}} \nonumber \\&= {} -\sigma _{\alpha \alpha }\sum _{i}\sum _{\mathbf {l}}\hbar \Theta _{i\mathbf { l}}^{\left( \alpha \right) }\left( d_{\mathbf {l}}^{\dagger }+d_{\mathbf {l} }\right) \left( b_{i}^{\left( \alpha \right) \dagger }+b_{i}^{\left( \alpha \right) }\right) \text {.} \end{aligned}$$

Intramolecular vibrational redistribution (IVR) as derived in “Appendix 1” is due to $V_{\mathrm {vib-vib}}=\sum _{\alpha }V_{\mathrm { vib-vib}}^{\left( \alpha \right) }$,

9 $$\begin{aligned} V_{\mathrm {vib-vib}}^{\left( \alpha \right) }&= {} \sigma _{\alpha \alpha }\sum _{\mathbf {l}}\frac{1}{2}m_{\mathbf {l}}\omega _{\mathbf {l} }^{2}\sum _{i<j}x_{i}^{\left( \alpha \right) }x_{j}^{\left( \alpha \right) }=\sigma _{\alpha \alpha }\sum _{i<j}k_{ij}^{\left( \alpha \right) }x_{i}^{\left( \alpha \right) }x_{j}^{\left( \alpha \right) } \nonumber \\&= {} \sigma _{\alpha \alpha }\sum _{i<j}\hbar \chi _{ij}^{\left( \alpha \right) }\left( b_{i}^{\left( \alpha \right) \dagger }+b_{i}^{\left( \alpha \right) }\right) \left( b_{j}^{\left( \alpha \right) \dagger }+b_{j}^{\left( \alpha \right) }\right) \end{aligned}$$

where $k_{ij}^{\left( \alpha \right) }=\sum _{\mathbf {l}}\frac{1}{2}m_{ \mathbf {l}}\omega _{\mathbf {l}}^{2}$ and the coupling constants are defined as

10 $$\begin{aligned} \Theta _{i\mathbf {l}}^{\left( \alpha \right) }&= {} \frac{1}{\hbar }m_{\mathbf { l}}\omega _{\mathbf {l}}^{2}\sqrt{\frac{\hbar }{2m_{\mathbf {l}}\omega _{ \mathbf {l}}}}\sqrt{\frac{\hbar }{2m_{i}\tilde{v}_{i}^{\left( \alpha \right) } }\text {,}} \end{aligned}$$

11 $$\begin{aligned} \chi _{ij}^{\left( \alpha \right) }&= {} \frac{1}{\hbar }k_{ij}^{\left( \alpha \right) }\sqrt{\frac{\hbar }{2m_{i}\tilde{v}_{i}^{\left( \alpha \right) }}} \sqrt{\frac{\hbar }{2m_{j}\tilde{v}_{j}^{\left( \alpha \right) }}}\text {.} \end{aligned}$$

The effect of displaced excited state PES has been discussed and the closed form of the overlap integral between harmonic potentials with different frequencies has been obtained in several works^22,23^ but the existing works do not take into account dissipation due to phonon reservoir. If we neglect IVR and assume $v_{i}^{\left( g\right) }=v_{i}^{\left( e\right) }$, Eq. (1) reduces to what is commonly referred to as the “Holstein Hamiltonian”^19,20^. Therefore, one can see that we are introducing a more general model in this section.

### Molecule–radiation interaction

The interaction Hamiltonian between a molecule and radiation which gives rise to spontaneous emission has the form $H_{\mathrm {mol-rad}}=-{\mu }\cdot \mathbf {E}$ where ${\mu }$ denotes the electronic dipole operator. Expressing ${\mu }$ in a complete basis, one can write

12 $$\begin{aligned} {\mu }&= {} \left( \left| g\right\rangle \left\langle g\right| +\left| e\right\rangle \left\langle e\right| \right) {\mu } \left( \left| g\right\rangle \left\langle g\right| +\left| e\right\rangle \left\langle e\right| \right) ={\mu }_{eg}\sigma ^{\dagger }+{\mu }_{ge}\sigma \nonumber \\&= {} \sum _{i}\left\{ \mathbf {M}_{i}^{\left( eg\right) }\left( x_{i}^{\left( g\right) },x_{i}^{\left( e\right) }\right) \sigma ^{\dagger }+\mathbf {M} _{i}^{\left( ge\right) }\left( x_{i}^{\left( g\right) },x_{i}^{\left( e\right) }\right) \sigma \right\} \end{aligned}$$

where we have defined the dipole matrix elements ${\mu } _{ge}=\left\langle g\right| {\mu }\left| e\right\rangle =\sum _{i}\mathbf {M}_{i}^{\left( ge\right) }\left( x_{i}^{\left( g\right) },x_{i}^{\left( e\right) }\right)$ with $x_{i}^{\left( g\right) }$ and $x_{i}^{\left( e\right) }$ dependence. These are the nuclear coordinates of the molecule we introduced earlier. Neglecting the nuclear dependence is referred to as the Franck–Condon approximation but we relax this approximation in this work. The electric field operator *E* can be written as^34^

13 $$\begin{aligned} \mathbf {E(r)=}\sum _{\mathbf {k}}\left( \vec {E}_{\mathbf {k}}a_{\mathbf {k}}+ \vec {E}_{\mathbf {k}}^{*}a_{\mathbf {k}}^{\dagger }\right) \mathbf {\ } \end{aligned}$$

with $\vec {E}_{\mathbf {k}}=\sqrt{\frac{\hbar \omega _{\mathbf {k}}}{ 2\varepsilon _{0}V}}{\varepsilon }_{\mathbf {k}}e^{i\mathbf {k\cdot r}}$. Here ${\varepsilon }_{\mathbf {k}}$ denotes the unit polarization vector, $\omega _{\mathbf {k}}$ the frequency of photon mode $\mathbf {k}$, *V* the quantization volume and $\ \varepsilon _{0}$ the permittivity of free space. We can write down the interaction Hamiltonian:

14 $$\begin{aligned} H_{\mathrm {mol-rad}}=-\sum _{i}\sum _{\mathbf {k}}\left( \mathbf {M}_{i}^{\left( eg\right) }\left( x_{i}^{\left( g\right) },x_{i}^{\left( e\right) }\right) \sigma ^{\dagger }+\mathbf {M}_{i}^{\left( ge\right) }\left( x_{i}^{\left( g\right) },x_{i}^{\left( e\right) }\right) \sigma \right) \cdot \left( \vec {E }_{\mathbf {k}}a_{\mathbf {k}}+\vec {E}_{\mathbf {k}}^{*}a_{\mathbf {k} }^{\dagger }\right) \text {.} \end{aligned}$$

The dipole matrix elements can be expanded with respect to the nuclear dependence using Taylor expansion

15 $$\begin{aligned} \mathbf {M}_{i}^{\left( eg\right) }\left( x_{i}^{\left( g\right) },x_{i}^{\left( e\right) }\right) =\mathbf {M}_{i}^{\left( eg\right) 0}+ \mathbf {M}_{i}^{\left( eg\right) g}\frac{x_{i}^{\left( g\right) }}{ a_{i}^{\left( g\right) }}+\mathbf {M}_{i}^{\left( eg\right) e}\frac{ x_{i}^{\left( e\right) }}{a_{i}^{\left( e\right) }}+\dots \end{aligned}$$

normalized by $a_{i}^{\left( \alpha \right) }=\sqrt{\frac{\hbar }{2m_{i} \tilde{v}_{i}^{\left( \alpha \right) }}}$ where higher order terms have been dropped and we only focus on the first order (Herzberg–Teller) term. The molecule-radiation Hamiltonian can thus be written as

16 $$\begin{aligned} H_{\mathrm {mol-rad}}&= {} -\sum _{i}\sum _{\mathbf {k}}\left[ \mathbf {M} _{i}^{\left( eg\right) 0}\sigma ^{\dagger }\cdot \left( \vec {E}_{\mathbf {k} }a_{\mathbf {k}}+\vec {E}_{\mathbf {k}}^{*}a_{\mathbf {k}}^{\dagger }\right) +\mathbf {M}_{i}^{\left( ge\right) 0}\sigma \cdot \left( \vec {E}_{\mathbf {k} }a_{\mathbf {k}}+\vec {E}_{\mathbf {k}}^{*}a_{\mathbf {k}}^{\dagger }\right) \right] \nonumber \\&-\sum _{\alpha =\left\{ g,e\right\} }\sum _{i}\sum _{\mathbf {k}}\left[ \mathbf {M}_{i}^{\left( eg\right) \left( \alpha \right) }x_{i}^{\left( \alpha \right) }\sigma ^{\dagger }\cdot \left( \vec {E}_{\mathbf {k}}a_{\mathbf {k}}+ \vec {E}_{\mathbf {k}}^{*}a_{\mathbf {k}}^{\dagger }\right) +\mathbf {M} _{i}^{\left( ge\right) \left( \alpha \right) }x_{i}^{\left( \alpha \right) }\sigma \cdot \left( \vec {E}_{\mathbf {k}}a_{\mathbf {k}}+\vec {E}_{\mathbf {k} }^{*}a_{\mathbf {k}}^{\dagger }\right) \right] \nonumber \\&= {} V_{\mathrm {el-rad}}+V_{\mathrm {el-vib-rad}} \end{aligned}$$

where the first coupling term $V_{\mathrm {el-rad}}$ corresponds to the usual fluorescence process under Franck–Condon approximation while the second coupling term $V_{\mathrm {el-vib-rad}}=\sum _{\alpha =\left\{ g,e\right\} }V_{ \mathrm {el-vib-rad}}^{\left( \alpha \right) }$ corresponds to Herzberg–Teller interaction.

Using the bosonic operators we introduced earlier in equation 6, we have

17 $$\begin{aligned} V_{\mathrm {el-rad}}&= {} -\hbar \sum _{i}\sum _{\mathbf {k}}\left[ \sigma ^{\dagger }\left( \varsigma _{i,\mathbf {k}}^{\left( eg\right) }a_{\mathbf {k} }+\varsigma _{i,\mathbf {k}}^{\left( eg\right) *}a_{\mathbf {k}}^{\dagger }\right) +\sigma \left( \varsigma _{i,\mathbf {k}}^{\left( ge\right) }a_{ \mathbf {k}}+\varsigma _{i,\mathbf {k}}^{\left( ge\right) *}a_{\mathbf {k} }^{\dagger }\right) \right] \text {,} \end{aligned}$$

18 $$\begin{aligned} V_{\mathrm {el-vib-rad}}^{\left( \alpha \right) }&= {} -\hbar \sum _{i}\left( b_{i}^{\left( \alpha \right) \dagger }+b_{i}^{\left( \alpha \right) }\right) \sum _{\mathbf {k}}\left[ \sigma ^{\dagger }\left( \xi _{i,\mathbf {k}}^{\left( eg\right) \left( \alpha \right) }a_{\mathbf {k}}+\xi _{i,\mathbf {k}}^{\left( eg\right) \left( \alpha \right) *}a_{\mathbf {k}}^{\dagger }\right) +\sigma \left( \xi _{i,\mathbf {k}}^{\left( ge\right) \left( \alpha \right) }a_{\mathbf {k}}+\xi _{i,\mathbf {k}}^{\left( ge\right) \left( \alpha \right) *}a_{\mathbf {k}}^{\dagger }\right) \right] \text {.} \end{aligned}$$

The counter-rotating terms referred to the terms that do not conserve energy (see Fig. 1). These are the terms $\propto \sigma a_{\mathbf { k}}$ or $\sigma ^{\dagger }a_{\mathbf {k}}^{\dagger }$ when Eqs. (17) and (18) are expanded. These terms are usually dropped when considering longer time-scale by invoking the rotating-wave approximation (RWA). But the validity of RWA has been questioned in recent papers^35–37^. Aiming to construct a generalized model, these counter-rotating terms are kept in this work as would be relevant at shorter timescales.

The coupling constants in Eqs. (17) and (18) are defined as $\varsigma _{i,\mathbf {k}}^{\left( eg\right) (*)}=\frac{1}{\hbar }\mathbf {M}_{i}^{\left( eg\right) 0}\cdot \vec {E}_{ \mathbf {k}}^{(*)}$ and $\xi _{i,\mathbf {k}}^{\left( eg\right) \left( \alpha \right) (*)}=\frac{1}{\hbar }\mathbf {M}_{i}^{\left( eg\right) \left( \alpha \right) }\cdot \vec {E}_{\mathbf {k}}^{(*)}$. If we assume $\varsigma _{i,\mathbf {k}}^{\left( ge\right) }=\varsigma _{i,\mathbf {k} }^{\left( eg\right) }$ and $\xi _{\mathbf {k},i}^{\left( eg\right) \left( \alpha \right) }=\xi _{\mathbf {k},i}^{\left( ge\right) \left( \alpha \right) }=\xi _{\mathbf {k},i}^{\left( \alpha \right) }$, Eqs. (17) and (18) reduces to a simpler form

19 $$\begin{aligned} V_{\mathrm {el-rad}}&= {} -\hbar \sum _{i}\sum _{\mathbf {k}}\varsigma _{i,\mathbf { k}}^{\left( eg\right) }\left( \sigma ^{\dagger }+\sigma \right) \left( a_{ \mathbf {k}}+a_{\mathbf {k}}^{\dagger }\right) \text {,} \end{aligned}$$

20 $$\begin{aligned} V_{\mathrm {el-vib-rad}}^{\left( \alpha \right) }&= {} -\hbar \sum _{i}\sum _{ \mathbf {k}}\xi _{\mathbf {k},i}^{\left( \alpha \right) }\left( b_{i}^{\left( \alpha \right) \dagger }+b_{i}^{\left( \alpha \right) }\right) \left( \sigma ^{\dagger }+\sigma \right) \left( a_{\mathbf {k}}^{\dagger }+a_{\mathbf {k} }\right) \text {.} \end{aligned}$$

### Total Hamiltonian

In this section, we give a summary of the results of this section. Starting with the total Hamiltonian given by $H=H_{\mathrm {mol-ph}}+H_{\mathrm {mol-rad }}+H_{\mathrm {rad}}+V_{C}$ where $H_{\mathrm {rad}}=\sum _{\mathbf {k}}\hbar \omega _{\mathbf {k}}\left( a_{\mathbf {k}}^{\dagger }a_{\mathbf {k}}+\frac{1}{2 }\right)$ is the radiation bath free-Hamiltonian and we inserted an additional term $V_{C}$ to take into account other coherent interaction that can take place within the system (such as external coherent fields). We expand the total Hamiltonian and group the individual terms as follows:

21 $$\begin{aligned} H&= {} \left( H_{\mathrm {el}}+H_{\mathrm {vib}}+H_{\mathrm {ph}}+V_{\mathrm { vib-vib}}+V_{\mathrm {el-vib-ph}}\right) +\left( V_{\mathrm {el-rad}}+V_{ \mathrm {el-vib-rad}}\right) +H_{\mathrm {rad}}+V_{C} \nonumber \\&= {} \left( H_{\mathrm {el}}+H_{\mathrm {vib}}\right) +\left( H_{\mathrm {ph}}+H_{ \mathrm {rad}}\right) +\left( V_{\mathrm {el-vib-ph}}+V_{\mathrm {el-rad}}+V_{ \mathrm {el-vib-rad}}\right) +\left( V_{\mathrm {vib-vib}}+V_{C}\right) \nonumber \\&= {} (H_{S}+H_{E})+(V_{SE}+V_{SC})=H_{0}+V \end{aligned}$$

where $H_{0}=H_{S}+H_{E}$ denotes free-Hamiltonian

22 $$\begin{aligned} H_{S}&= {} H_{\mathrm {el}}+H_{\mathrm {vib}}=\sum _{\alpha }\sigma _{\alpha \alpha }\left\{ \hbar \omega _{\alpha }+\sum _{i}\hbar \tilde{v}_{i}^{\left( \alpha \right) }\left( b_{i}^{\left( \alpha \right) \dagger }b_{i}^{\left( \alpha \right) }+\frac{1}{2}\right) \right\} \text {,} \end{aligned}$$

23 $$\begin{aligned} H_{E}&= {} H_{\mathrm {ph}}+H_{\mathrm {rad}}=\sum _{\mathbf {l}}\hbar \omega _{ \mathbf {l}}\left( d_{\mathbf {l}}^{\dagger }d_{\mathbf {l}}+\frac{1}{2}\right) +\sum _{\mathbf {k}}\hbar \omega _{\mathbf {k}}\left( a_{\mathbf {k}}^{\dagger }a_{\mathbf {k}}+\frac{1}{2}\right) \text {.} \end{aligned}$$

and the interaction Hamiltonian $V=V_{SE}+V_{SC}$ includes coherent coupling within the system ($V_{SC}$) and coupling to the environmental baths ($V_{SE}$)

24 $$\begin{aligned} V_{SC}&= {} V_{\mathrm {vib-vib}}+V_{C} \end{aligned}$$

25 $$\begin{aligned} V_{SE}&= {} V_{\mathrm {el-rad}}+V_{\mathrm {el-vib-rad}}+V_{\mathrm {el-vib-ph}} \text {.} \end{aligned}$$

Note that the the zero-point energy terms are included in the expressions above. This is to take into account the possibility when the frequency is different between the ground and excited PES. These terms will also contribute to the coherent evolution when the master equation is derived in the next section.

### Relationship between ground and excited vibrational operators

Consider the two states molecular Hamiltonians with harmonic PES

26 $$\begin{aligned} H_{\alpha }=\sigma _{\alpha \alpha }\left\{ \hbar \omega _{\alpha }+\sum _{i}\hbar \tilde{v}_{i}^{\left( \alpha \right) }\left( b_{i}^{\left( \alpha \right) \dagger }b_{i}^{\left( \alpha \right) }+\frac{1}{2}\right) \right\} \end{aligned}$$

For notational simplicity, consider single mode

27 $$\begin{aligned} x_{g}=\sqrt{\frac{\hbar }{2mv_{g}}}\left( b_{g}^{\dagger }+b_{g}\right) \text {, }p_{g}=i\sqrt{\frac{\hbar mv_{g}}{2}}\left( b_{g}^{\dagger }-b_{g}\right) \end{aligned}$$

The PES are displaced by *s*, the relationship $$x_{e}=x_{g}-s$$ and $$ p_{e}=p_{g}$$ gives

28 $$\begin{aligned} b_{e}=\gamma _{+}b_{g}+\gamma _{-}b_{g}^{\dagger }-\Lambda _{e} \end{aligned}$$

where

29 $$\begin{aligned} \gamma _{\pm }=\frac{1}{2}\left( \sqrt{\frac{v_{e}}{v_{g}}}\pm \sqrt{\frac{ v_{g}}{v_{e}}}\right) \text {, }\Lambda _{e/g}=s\sqrt{\frac{mv_{e/g}}{2\hbar } } \end{aligned}$$

with the relations $\gamma _{\pm }^{2}=\frac{1}{4}\left( \frac{v_{e}}{v_{g}}+ \frac{v_{g}}{v_{e}}\right) \pm \frac{1}{2},\gamma _{+}^{2}+\gamma _{-}^{2}= \frac{1}{2}\left( \frac{v_{e}}{v_{g}}+\frac{v_{g}}{v_{e}}\right) ,\gamma _{-}+\gamma _{+}=\sqrt{\frac{v_{e}}{v_{g}}}$.

### Interaction picture

The two states molecular Hamiltonian is $H_{g}+H_{e}=\tilde{H}_{\mathrm {el}}+ \tilde{H}_{\mathrm {vib}}+V_{\mathrm {el-vib}}$

30 $$\begin{aligned} \tilde{H}_{\mathrm {el}}&= {} \hbar \omega _{g}\sigma _{gg}+\{\hbar \omega _{e}+\frac{mv_{e}^{2}}{2}l^{2}\}\sigma _{ee} \end{aligned}$$

31 $$\begin{aligned} \tilde{H}_{\mathrm {vib}}&= {} \hbar v_{g}\left( b_{g}^{\dagger }b_{g}+\frac{1}{ 2}\right) \end{aligned}$$

32 $$\begin{aligned} V_{\mathrm {el-vib}}&= {} V_{\mathrm {el-vib}}^{\left( 1\right) }+V_{\mathrm { el-vib}}^{\left( 2\right) } \nonumber \\&= {} \left[ \hbar \eta ^{(1)}\left( b_{g}+b_{g}^{\dagger }\right) +\hbar \eta ^{(2)}\left( b_{g}^{\dagger }+b_{g}\right) ^{2}\right] \sigma _{ee} \nonumber \\&= {} \left[ xx_{0}\left\{ -mv^{\prime }{}^{2}-2W\right\} +x^{2}\frac{1}{2} m\left( v^{\prime }{}^{2}-v^{2}\right) \right] \sigma _{ee} \end{aligned}$$

where $$\eta ^{(1)}=-mv_{e}^{2}l\sqrt{\frac{1}{2mv_{g}\hbar }}=-v_{e}^{2}l \sqrt{\frac{m}{2v_{g}\hbar }},\eta ^{(2)}=\left( \frac{v_{e}^{2}-v_{g}^{2}}{ 4v_{g}}\right) $$.

In the transformation from Schrodinger picture to interaction picture the free Hamiltonian $H_{0}=(H_{\mathrm {el}}+H_{\mathrm {vib}})+(H_{\mathrm {rad} }+H_{\mathrm {ph}})$ does NOT include $V_{\mathrm {el-vib}}$. The matrix for $b_{i}^{\left( e\right) }$ is different from $b_{i}^{\left( g\right) }$ by factors $\frac{\tilde{v}_{i}^{\left( e\right) }}{\tilde{v} _{i}^{\left( g\right) }}$. Practically the difference $\tilde{v}_{i}^{\left( e\right) }-\tilde{v}_{i}^{\left( g\right) }$ is less than 10$^{12}$ s$^{-1}$ so the ratio $\frac{\tilde{v}_{i}^{\left( e\right) }}{\tilde{v}_{i}^{\left( g\right) }}\sim 1$. Hence it can be a good approximation (for three vibrational states) $$b_{i}^{\left( e\right) }=\left[ \begin{array}{ccc} \Lambda _{e} &{} \lambda _{i}^{\left( +\right) } &{} 0 \\ \lambda _{i}^{\left( -\right) } &{} \Lambda _{e} &{} \sqrt{2}\lambda _{i}^{\left( +\right) } \\ 0 &{} \sqrt{2}\lambda _{i}^{\left( -\right) } &{} \Lambda _{e} \end{array} \right] \sim \left[ \begin{array}{ccc} \Lambda _{e} &{} 1 &{} 0 \\ 0 &{} \Lambda _{e} &{} \sqrt{2} \\ 0 &{} 0 &{} \Lambda _{e} \end{array} \right] =b_{i}^{\left( g\right) }+\Lambda _{e}$$**.**

For an arbitrary operator *O*, we will use the “tilde” to denote its interaction picture $\tilde{O}\left( t\right) =e^{iH_{0}t/\hbar }Oe^{-iH_{0}t/\hbar }$. Denoting $\omega _{eg}=\omega _{e}-\omega _{g}$, the system and environment operators when expressed in the interaction picture are given by

33 $$\begin{aligned} \tilde{\sigma }\left( t\right)&= {} \sigma e^{-i\tilde{\omega }_{eg}t}\text {, } \tilde{b}_{i}^{\left( g\right) }\left( t\right) =b_{i}^{\left( g\right) }e^{-i\tilde{v}_{i}^{\left( g\right) }t}\text {,} \end{aligned}$$

34 $$\begin{aligned} \tilde{d}_{\mathbf {l}}\left( t\right)&= {} d_{\mathbf {l}}e^{-i\omega _{\mathbf { l}}t}\text {, }\tilde{a}_{\mathbf {k}}\left( t\right) =a_{\mathbf {k} }e^{-i\omega _{\mathbf {k}}t}\text {.} \end{aligned}$$

where

35 $$\begin{aligned} \tilde{\omega }_{eg}&= {} \hbar (\omega _{e}-\omega _{g})+\frac{mv_{e}^{2}}{2} l^{2}+l{}^{2}W \end{aligned}$$

36 $$\begin{aligned} \tilde{v}_{i}^{\left( g\right) 2}&= {} v_{i}^{\left( g\right) 2}+\frac{2W}{ m_{i}} \end{aligned}$$

Our theoretical model for the polyatomic molecule will include include the following processes:

- radiative transition via fluorescence described by the term $V_{\mathrm { el-rad}}$ (Franck–Condon) and first-order Non-Condon radiative transition described by the term $V_{\mathrm {el-vib-rad}}$ (Herzberg–Teller),
- non-radiative (NR) transition involving vibrational relaxation (VR) through the term $V_{\mathrm {el-vib-ph}}$ and intra-molecular vibrational energy redistribution (IVR) by the term $V_{\mathrm {vib-vib}}$.

The inclusion of internal conversion (IC) would require non-adiabatic process to be accounted beyond the BO approximation and is not the focus of this present work. Intersystem crossing involving electronic states of different multiplicities are also not discussed here.

A molecule in a higher excited singlet state $S_{n}$ with $n>1$ will rapidly undergo NR relaxations through internal conversion IC (between different electronic states), IVR (between different vibrational modes) and vibrational relaxation VR (within the same electronic potential) to the vibrational ground state of the $S_{1}$ before fluorescence takes place (radiative transition). Hence, only vibrational ground state of $S_{1}\longrightarrow S_{0}$ fluorescence is expected in the emission spectra, this is commonly referred to as Kasha’s rule (depicted in Fig. 1).

## Dynamics of the quantum system

We now develop a master equation under the Markov approximation for phonon and radiation baths which will be implemented into the Python package QuTiP^33^ where numerical simulations can be performed. As previously mentioned in the introduction, when dealing with a multipartite system (e.g. molecule), a common approach is to apply the assumption that each part of the system interacts with their own environment independently. Here we relax this assumption and we will see that leads to the “cross-term Liouvillean”. The derivation of master equation can be found in several textbooks^38,39^, in “Appendix 3” we provide a generalized derivation of the master equation that is used in this section to show how the “cross-term Liouvillean” arises.

### Master equation and internal coupling

Our starting point is the Liouville–von Neumann equation for the total density matrix $\rho _{T}\left( t\right)$ in the interaction picture

37 $$\begin{aligned} \frac{d}{dt}\tilde{\rho }_{T}\left( t\right) =-\frac{i}{\hbar }\left[ \tilde{V }\left( t\right) ,\tilde{\rho }_{T}\left( t\right) \right] \end{aligned}$$

where we recall that

38 $$\begin{aligned} \tilde{V}\left( t\right) =\tilde{V}_{SC}\left( t\right) +\tilde{V} _{SE}\left( t\right) \end{aligned}$$

with $V_{SC}$ describes the coherent internal coupling terms within the system and $V_{SE}$ describes coupling terms between the system and the environment (phonons and radiation). Substituting Eq. (38) into (37), we get

39 $$\begin{aligned} \frac{d}{dt}\tilde{\rho }_{T}\left( t\right) =-\frac{i}{\hbar }\left[ \tilde{V }_{SC}\left( t\right) +\tilde{V}_{SE}\left( t\right) ,\tilde{\rho }_{T}\left( t\right) \right] \end{aligned}$$

which is separated into a coherent and dissipation part. We follow the standard procedure to substitute the formal integration solution back into the expression above and invoke the Born approximation. Upon tracing out the environment, we obtain

40 $$\begin{aligned} \frac{d}{dt}\tilde{\rho }_{S}\left( t\right) =-\frac{i}{\hbar }\left[ \tilde{V }_{SC}\left( t\right) ,\tilde{\rho }_{T}\left( t\right) \right] -\frac{1}{ \hbar ^{2}}\int _{0}^{t}dt^{\prime }Tr_{E}\left[ \tilde{V}_{SE}\left( t\right) ,\left[ \tilde{V}_{SE}\left( t^{\prime }\right) ,\tilde{\rho } _{S}\left( t^{\prime }\right) \otimes \rho _{E}\right] \right] \end{aligned}$$

where $\rho _{S}=Tr_{E}\left[ \rho _{T}\right] =Tr_{\mathrm {rad}}Tr_{\mathrm { ph}}\left[ \rho _{T}\right]$ is the reduced system density operator and $\rho _{E}=\rho _{ph}\otimes \rho _{rad}$.

Recall from “The model” section, we have grouped all the coherent coupling terms within the system in $V_{SC}$ while $V_{SE}$ includes coupling terms between the system and the environment. In the interaction picture, we have

41 $$\begin{aligned} \tilde{V}_{SE}\left( t\right) =\tilde{V}_{\mathrm {el-vib-ph}}\left( t\right) +\tilde{V}_{\mathrm {el-rad}}\left( t\right) +\tilde{V}_{\mathrm {el-vib-rad} }\left( t\right) \text {.} \end{aligned}$$

Upon substituting Eq. (41) into Eq. (40), we obtain overlapping (crossed) terms between different interaction terms in the integral:

42 $$\begin{aligned} \sum \limits _{\mathrm {E}}\sum \limits _{\mathrm {X,Y}}Tr_{E}\left[ \tilde{V}_{ \mathrm {X-E}}\left( t\right) ,\left[ \tilde{V}_{\mathrm {Y-E}}\left( t^{\prime }\right) ,\tilde{\rho }_{S}\left( t^{\prime }\right) \otimes \rho _{E}\right] \right]&= {} Tr_{\mathrm {ph}}\left[ \tilde{V}_{\mathrm {el-vib-ph} }\left( t\right) ,\left[ \tilde{V}_{\mathrm {el-vib-ph}}\left( t^{\prime }\right) ,\tilde{\rho }_{S}\left( t^{\prime }\right) \otimes \rho _{\mathrm {ph }}\right] \right] \nonumber \\&\quad +\, Tr_{\mathrm {rad}}\left[ \tilde{V}_{\mathrm {el-rad}}\left( t\right) ,\left[ \tilde{V}_{\mathrm {el-rad}}\left( t^{\prime }\right) ,\tilde{\rho }_{S}\left( t^{\prime }\right) \otimes \rho _{\mathrm {rad}}\right] \right] \nonumber \\&\quad +\,Tr_{\mathrm {rad}}\left[ \tilde{V}_{\mathrm {el-vib-rad}}\left( t\right) , \left[ \tilde{V}_{\mathrm {el-vib-rad}}\left( t^{\prime }\right) ,\tilde{\rho } _{S}\left( t^{\prime }\right) \otimes \rho _{\mathrm {rad}}\right] \right] \nonumber \\&\quad +\,Tr_{\mathrm {rad}}\left[ \tilde{V}_{\mathrm {el-rad}}\left( t\right) ,\left[ \tilde{V}_{\mathrm {el-vib-rad}}\left( t^{\prime }\right) ,\tilde{\rho } _{S}\left( t^{\prime }\right) \otimes \rho _{\mathrm {rad}}\right] \right] \nonumber \\&\quad +\,Tr_{\mathrm {rad}}\left[ \tilde{V}_{\mathrm {el-vib-rad}}\left( t\right) , \left[ \tilde{V}_{\mathrm {el-rad}}\left( t^{\prime }\right) ,\tilde{\rho } _{S}\left( t^{\prime }\right) \otimes \rho _{\mathrm {rad}}\right] \right] \end{aligned}$$

with $\mathrm {E}=\mathrm {ph}$,$\mathrm {rad}$ and $\mathrm {X,Y}= \mathrm {el,el-vib}$ (but note that there is no $\mathrm {el-ph}$ interaction term). The non-overlapping terms (i.e. first three lines on the RHS of Eq. (42)) are represented by the Liouvillean

43 $$\begin{aligned} \mathscr {L}_{\mathrm {X-E}}=-\frac{1}{\hbar ^{2}}\int _{0}^{t}dt^{\prime }Tr_{ \mathrm {E}}\left[ \tilde{V}_{\mathrm {X-E}}\left( t\right) ,\left[ \tilde{V}_{ \mathrm {X-E}}\left( t^{\prime }\right) ,\tilde{\rho }_{S}\left( t^{\prime }\right) \otimes \rho _{\mathrm {E}}\right] \right] \end{aligned}$$

while the other two terms (i.e. last two lines on the RHS of Eq. (42)) are represented by the Liouvillean

44 $$\begin{aligned} \mathscr {L}_{\mathrm {\{X\times Y\}-E}}&= {} -\frac{1}{\hbar ^{2}} \int _{0}^{t}dt^{\prime }Tr_{\mathrm {E}}\left[ \tilde{V}_{\mathrm {X-E}}\left( t\right) ,\left[ \tilde{V}_{\mathrm {Y-E}}\left( t^{\prime }\right) ,\tilde{ \rho }_{S}\left( t^{\prime }\right) \otimes \rho _{\mathrm {E}}\right] \right] \\&\quad -\, \frac{1}{\hbar ^{2}}\int _{0}^{t}dt^{\prime }Tr_{\mathrm {E}}\left[ \tilde{V }_{\mathrm {Y-E}}\left( t\right) ,\left[ \tilde{V}_{\mathrm {X-E}}\left( t^{\prime }\right) ,\tilde{\rho }_{S}\left( t^{\prime }\right) \otimes \rho _{ \mathrm {E}}\right] \right] \text {.} \nonumber \end{aligned}$$

Hence, the master equation when written out explicitly has the following form

45 $$\begin{aligned} \frac{d}{dt}\tilde{\rho }_{S}\left( t\right) =-\frac{i}{\hbar }\left[ \tilde{V }_{SC}\left( t\right) ,\tilde{\rho }_{T}\left( t\right) \right] +\mathscr {L}_{ \mathrm {el-vib-ph}}+\mathscr {L}_{\mathrm {el-rad}}+\mathscr {L}_{\mathrm { el-vib-rad}}+\mathscr {L}_{\mathrm {\{el\times (el-vib)\}-rad}} \end{aligned}$$

where here after we will refer $\mathscr {L}_{\mathrm {\{el\times (el-vib)\}-rad}}$ as the crossed-term Liouvillean.

### Liouvilleans of $\mathrm {el-vib-ph}$ interaction

The Liouvillean for $\mathrm {el-vib-ph}$ interaction is derived in a standard manner from

46 $$\begin{aligned} \tilde{V}_{\mathrm {el-vib-ph}}^{\left( \alpha \right) }=-\sigma _{\alpha \alpha }\sum _{i}\sum _{\mathbf {l}}\hbar \Theta _{i\mathbf {l}}^{\left( \alpha \right) }\left( d_{\mathbf {l}}^{\dagger }e^{i\omega _{l}t}+d_{\mathbf {l} }e^{-i\omega _{l}t}\right) \left( b_{i}^{\left( \alpha \right) \dagger }e^{iv_{i}^{\left( \alpha \right) }t}+b_{i}^{\left( \alpha \right) }e^{-iv_{i}^{\left( \alpha \right) }t}\right) \end{aligned}$$

only with Markov approximation. By defining $S_{\alpha i}=\sigma _{\alpha \alpha }b_{i}^{\left( \alpha \right) }$, $S_{\alpha i}^{\dagger }=\sigma _{\alpha \alpha }b_{i}^{\left( \alpha \right) \dagger }$ and $\Delta \tilde{v }_{i,i^{\prime }}^{\left( \pm \right) \left( \alpha ,\alpha ^{\prime }\right) }=\tilde{v}_{i}^{\left( \alpha \right) }\pm \tilde{v}_{i^{\prime }}^{\left( \alpha ^{\prime }\right) }$, the Liouvillean is written in a compact form $\mathscr {L}_{\mathrm {el-vib-ph}}=\sum _{\alpha ,\alpha ^{\prime },i,i^{\prime }}\mathscr {L}_{\mathrm {el-vib-ph}}^{\left( \alpha ,\alpha ^{\prime },i,i^{\prime }\right) }=\sum _{*}\mathscr {L}_{\mathrm {el-vib-ph} }^{\left( *\right) }$ where $*$ is a symbol we introduced for $\left( \alpha ,\alpha ^{\prime },i,i^{\prime }\right)$ that shows up frequently in this work. The explicit form of the Liouvillean can be derived using the method given in “Appendix 3”

47 $$\begin{aligned} \mathscr {L}_{\mathrm {el-vib-ph}}^{\left( *\right) }&= {} -\Upsilon _{\left\{ n+1\right\} }^{\left( *\right) }\left( \left( S_{\alpha i}^{\dagger }S_{\alpha ^{\prime }i^{\prime }}\tilde{\rho }_{S}\left( t\right) -S_{\alpha ^{\prime }i^{\prime }}\tilde{\rho }_{S}\left( t\right) S_{\alpha i}^{\dagger }\right) e^{i\Delta \tilde{v}_{i,i^{\prime }}^{\left( -\right) \left( \alpha ,\alpha ^{\prime }\right) }t}\right. \nonumber \\&\left. \quad +\, \left( \tilde{\rho }_{S}\left( t\right) S_{\alpha ^{\prime }i^{\prime }}^{\dagger }S_{\alpha i}^{\dagger }-S_{\alpha i}^{\dagger }\tilde{\rho }_{S}\left( t\right) S_{\alpha ^{\prime }i^{\prime }}^{\dagger }\right) e^{i\Delta \tilde{v}_{i,i^{\prime }}^{\left( +\right) \left( \alpha ,\alpha ^{\prime }\right) }t}\right) \nonumber \\&\quad -\, \Upsilon _{\left\{ n\right\} }^{\left( *\right) }\left( \left( \tilde{ \rho }_{S}\left( t\right) S_{\alpha ^{\prime }i^{\prime }}S_{\alpha i}^{\dagger }-S_{\alpha i}^{\dagger }\tilde{\rho }_{S}\left( t\right) S_{\alpha ^{\prime }i^{\prime }}\right) e^{i\Delta \tilde{v}_{i,i^{\prime }}^{\left( -\right) \left( \alpha ,\alpha ^{\prime }\right) }t}\right. \nonumber \\&\left. \quad +\, \left( S_{\alpha i}^{\dagger }S_{\alpha ^{\prime }i^{\prime }}^{\dagger }\tilde{\rho }_{S}\left( t\right) -S_{\alpha ^{\prime }i^{\prime }}^{\dagger }\tilde{\rho }_{S}\left( t\right) S_{\alpha i}^{\dagger }\right) e^{i\Delta \tilde{v}_{i,i^{\prime }}^{\left( +\right) \left( \alpha ,\alpha ^{\prime }\right) }t}\right) +H.c.+\Lambda _{\mathrm { el-vib-ph}}^{(*)}\text {.} \end{aligned}$$

Here we stress the important result of Eq. (47). In the case of a diatomic molecule with single vibrational mode, we may simply set $i=i^{\prime }$ (or dropping the indices *i*) and the exponentials with $\Delta \tilde{v}_{i,i^{\prime }}^{\left( -\right) \left( \alpha ,\alpha ^{\prime }\right) }$ vanish. However, the time-dependent exponentials do not vanish even in the case of a simple diatomic molecule because they are consequence of the counter-rotating terms. Therefore, we can see that multi-vibrational modes in polyatomic molecule lead to the time-dependence that gives beating dynamics while counter-rotating terms lead to the more rapid time-dependence (due to $\left( +\right)$ superscripts). The last term $\Lambda _{\mathrm {el-vib-ph}}^{\left( *\right) }$ in Eq. (47) is the Lamb shift term given by Eq. (125) in “Appendix 5”. The decay constants are defined as

48 $$\begin{aligned} \Upsilon _{\left\{ \ldots \right\} }^{\left( *\right) }=\Upsilon _{\left\{ \cdots \right\} }^{\left( \alpha ,\alpha ^{\prime },i,i^{\prime }\right) }\left( \tilde{v}_{i^{\prime }}^{\left( \alpha ^{\prime }\right) }\right) =\int d^{3}l~\digamma \left( \mathbf {l}\right) \Theta _{i\mathbf {l}}^{\left( \alpha \right) }\Theta _{i^{\prime }\mathbf {l}}^{\left( \alpha ^{\prime }\right) }\pi \delta \left( \omega _{l}-\tilde{v}_{i^{\prime }}^{\left( \alpha ^{\prime }\right) }\right) \left\{ \cdots \right\} _{\mathbf {l}}= \frac{\lambda }{\omega _{c}^{2}}\left( \tilde{v}_{i^{\prime }}^{\left( \alpha ^{\prime }\right) }\right) ^{3}\exp \left( -\tilde{v}_{i^{\prime }}^{\left( \alpha ^{\prime }\right) }/\omega _{c}\right) \left\{ \cdots \right\} _{\tilde{v}_{i^{\prime }}^{\left( \alpha ^{\prime }\right) }} \end{aligned}$$

where

49 $$\begin{aligned} \int d^{3}l~\digamma \left( \mathbf {l}\right) \Theta _{i\mathbf {l}}^{\left( \alpha \right) }\Theta _{i^{\prime }\mathbf {l}}^{\left( \alpha ^{\prime }\right) }..\longrightarrow \int d\omega _{\mathbf {l}}J_{i,i^{\prime }}^{\left( \alpha ,\alpha ^{\prime }\right) }\left( \omega _{\mathbf {l} }\right) ..=\pi J_{i,i^{\prime }}^{\left( \alpha ,\alpha ^{\prime }\right) }\left( \tilde{v}_{i^{\prime }}^{\left( \alpha ^{\prime }\right) }\right) \left\{ \cdots \right\} _{\tilde{v}_{i^{\prime }}^{\left( \alpha ^{\prime }\right) }} \end{aligned}$$

is the spectral density (^12^) of the phonons. The thermal average phonon number is

50 $$\begin{aligned} \bar{n}\left( \omega _{l}\right) =\left\langle d_{\mathbf {l}}^{\dagger }d_{ \mathbf {l}}\right\rangle _{\mathrm {ph}}=\frac{1}{e^{\hbar \omega _{l}/k_{B}T}-1} \end{aligned}$$

where $\left\{ \cdots \right\} =\left\{ \bar{n},\bar{n}+1\right\}$ and $\digamma \left( \mathbf {l}\right)$ is the phonon density of states. Here a super-Ohmic bath has been chosen for the phonon bath to model realistic three-dimensional acoustic phonon (see “Appendix 4”), $\lambda$ and $\omega _{c}$ are the overall coupling strength and the phonon cut-off frequency.

Note that the cross term Liouvilleans correspond to those with $\alpha \ne \alpha ^{\prime }$ in the above The following formula $\lim _{t\rightarrow \infty }\int _{0}^{t}d\tau e^{\pm ix\tau }=\pi \delta \left( x\right) \pm i \frac{P}{x}$ is an approximation. In general

51 $$\begin{aligned} \int _{0}^{t}d\tau e^{\pm ix\tau }\cdots =\int _{0}^{t}d\tau (\cos x\tau \pm i\sin x\tau )\ldots \text {.} \end{aligned}$$

### Liouvillean of $\mathrm {el-rad}$ interaction (Condon approximation)

One can refer to any standard text for the detailed derivation, the result we obtained is

52 $$\begin{aligned} \mathscr {L}_{\mathrm {el-rad}}&= {} \Gamma _{\left\{ n+1\right\} }\left[ 2\sigma \tilde{\rho }_{S}\left( t\right) \sigma ^{\dagger }-\left\{ \sigma ^{\dagger }\sigma ,\tilde{\rho }_{S}\left( t\right) \right\} \right] +\Gamma _{\left\{ n\right\} }\left[ 2\sigma ^{\dagger }\tilde{\rho }_{S}\left( t\right) \sigma -\left\{ \sigma \sigma ^{\dagger },\tilde{\rho }_{S}\left( t\right) \right\} \right] \nonumber \\&+\Gamma _{\left\{ 2n+1\right\} }\left[ \sigma ^{\dagger }\tilde{\rho } _{S}\left( t\right) \sigma ^{\dagger }e^{i2\omega _{eg}t}+\sigma \tilde{\rho } _{S}\left( t\right) \sigma e^{-i2\omega _{eg}t}\right] +\Lambda _{\mathrm { el-rad}} \end{aligned}$$

where the Lamb shift $\Lambda _{\mathrm {el-rad}}$ is given by Eq. (128) and we have defined the decay term (obtained in “Appendix 4”)

53 $$\begin{aligned} \Gamma _{\left\{ \cdots \right\} }\left( \omega _{eg}\right)&= {} \sum _{i,i^{\prime }}\int d^{3}k~D\left( \mathbf {k}\right) \varsigma _{i, \mathbf {k}}^{\left( ge\right) }\varsigma _{i^{\prime },\mathbf {k}}^{\left( ge\right) }\pi \delta \left( \omega _{k}-\omega _{eg}\right) \left\{ \cdots \right\} _{\mathbf {k}} \nonumber \\&= {} \sum _{i,i^{\prime }}\frac{1}{2}\frac{\omega _{eg}^{3}\left| \mathbf {M} _{i}^{\left( eg\right) 0}\right| \left| \mathbf {M}_{i^{\prime }}^{\left( eg\right) 0}\right| }{3\varepsilon _{0}\pi \hbar c^{3}} \left\{ \cdots \right\} _{eg} \end{aligned}$$

with the thermal average photon number

54 $$\begin{aligned} \bar{n}\left( \omega _{k}\right) =\left\langle a_{\mathbf {k}}^{\dagger }a_{ \mathbf {k}}\right\rangle _{\mathrm {rad}}=\frac{1}{e^{\hbar \omega _{k}/k_{B}T}-1}\text {.} \end{aligned}$$

The form of $\mathscr {L}_{\mathrm {el-rad}}$ is similar to the spontaneous emission Liouvillean in atomic system and in agreement with the study comparing non-Markovian and Markovian dynamics without RWA^40^ where time dependent decay rate is also obtained. This also helps confirm the calculation of $\mathscr {L}_{\mathrm {el-vib-ph}}^{\left( \alpha ,\alpha ^{\prime }\right) }$ in the previous section.

### Liouvilleans of $\mathrm {el-vib-rad}$ non-Condon interaction

In this section, we calculate the Liouvillean due to Herzberg—Teller interaction Hamiltonian. The $\mathrm {el-vib-rad}$ interaction

55 $$\begin{aligned} V_{\mathrm {el-vib-rad}}^{\left( \alpha \right) }=-\hbar \sum _{i}\sum _{ \mathbf {k}}\xi _{\mathbf {k},i}^{\left( \alpha \right) }\left( b_{i}^{\left( \alpha \right) \dagger }+b_{i}^{\left( \alpha \right) }\right) \left( \sigma ^{\dagger }+\sigma \right) \left( a_{\mathbf {k}}+a_{\mathbf {k}}^{\dagger }\right) \end{aligned}$$

gives the Liouvillean

56 $$\begin{aligned} \mathscr {L}_{\mathrm {el-vib-rad}}=\sum _{*}\mathscr {L}_{\mathrm {el-vib-rad }}^{\left( *\right) }=\mathscr {L}_{\mathrm {el-vib-rad}}^{\left( *\right) \left( +\right) }+\mathscr {L}_{\mathrm {el-vib-rad}}^{\left( *\right) \left( -\right) }+\Lambda _{\mathrm {el-vib-rad}}^{\left( *\right) } \end{aligned}$$

The Lamb shift term $\Lambda _{\mathrm {el-vib-rad}}^{\left( *\right) }$ is given in Eq. (132), in order to express this Liouvillean in a compact way, we introduced the following definition:

57 $$\begin{aligned} B_{\alpha i}&= {} b_{i}^{\left( \alpha \right) }\sigma \text {, }B_{\alpha i}^{\dagger }=b_{i}^{\left( \alpha \right) \dagger }\sigma ^{\dagger }\text {,} \end{aligned}$$

58 $$\begin{aligned} C_{\alpha i}&= {} b_{i}^{\left( \alpha \right) }\sigma ^{\dagger }\text {, } C_{\alpha i}^{\dagger }=b_{i}^{\left( \alpha \right) \dagger }\sigma \text {.} \end{aligned}$$

Again, following the method established in “Appendix 3”, we obtain

59 $$\begin{aligned} \mathscr {L}_{\mathrm {el-vib-rad}}^{\left( *\right) \left( +\right) }&= {} - \tilde{\Gamma }_{\left\{ n+1\right\} }^{\left( *\right) +}\left( \left( B_{\alpha i}^{\dagger }B_{\alpha ^{\prime }i^{\prime }}\tilde{\rho } _{S}\left( t\right) -B_{\alpha ^{\prime }i^{\prime }}\tilde{\rho }_{S}\left( t\right) B_{\alpha i}^{\dagger }\right) e^{i\Delta \tilde{v}_{i,i^{\prime }}^{\left( -\right) \left( \alpha ,\alpha ^{\prime }\right) }t}+\left( \tilde{\rho }_{S}\left( t\right) B_{\alpha ^{\prime }i^{\prime }}^{\dagger }C_{\alpha i}^{\dagger }-C_{\alpha i}^{\dagger }\tilde{\rho }_{S}\left( t\right) B_{\alpha ^{\prime }i^{\prime }}^{\dagger }\right) e^{i\Delta \tilde{v}_{i,i^{\prime }}^{\left( +\right) \left( \alpha ,\alpha ^{\prime }\right) }t}\right. \nonumber \\&\left. \quad -\, B_{\alpha i}^{\dagger }\tilde{\rho }_{S}\left( t\right) B_{\alpha ^{\prime }i^{\prime }}^{\dagger }e^{i\left( \Delta \tilde{ v}_{i,i^{\prime }}^{\left( +\right) \left( \alpha ,\alpha ^{\prime }\right) }+2\omega _{eg}\right) t}-B_{\alpha ^{\prime }i^{\prime }}\tilde{\rho } _{S}\left( t\right) C_{\alpha i}^{\dagger }e^{i\left( \Delta \tilde{v} _{i,i^{\prime }}^{\left( -\right) \left( \alpha ,\alpha ^{\prime }\right) }-2\omega _{eg}\right) t}\right) \nonumber \\&\quad -\, \tilde{\Gamma }_{\left\{ n\right\} }^{\left( *\right) +}\left( \left( \tilde{\rho }_{S}\left( t\right) B_{\alpha ^{\prime }i^{\prime }}B_{\alpha ^{\prime }i^{\prime }}^{\dagger }-B_{\alpha i}^{\dagger }\tilde{\rho } _{S}\left( t\right) B_{\alpha ^{\prime }i^{\prime }}\right) e^{i\Delta \tilde{v}_{i,i^{\prime }}^{\left( -\right) \left( \alpha ,\alpha ^{\prime }\right) }t}+\left( C_{\alpha i}^{\dagger }B_{\alpha ^{\prime }i^{\prime }}^{\dagger }\tilde{\rho }_{S}\left( t\right) -B_{\alpha ^{\prime }i^{\prime }}^{\dagger }\tilde{\rho }_{S}\left( t\right) C_{\alpha i}^{\dagger }\right) e^{i\Delta \tilde{v}_{i,i^{\prime }}^{\left( +\right) \left( \alpha ,\alpha ^{\prime }\right) }t}\right. \nonumber \\&\quad \left. -\, C_{\alpha i}^{\dagger }\tilde{\rho }_{S}\left( t\right) B_{\alpha ^{\prime }i^{\prime }}e^{i\left( \Delta \tilde{v} _{i,i^{\prime }}^{\left( -\right) \left( \alpha ,\alpha ^{\prime }\right) }-2\omega _{eg}\right) t}-B_{\alpha i}^{\dagger }\tilde{\rho }_{S}\left( t\right) B_{\alpha ^{\prime }i^{\prime }}^{\dagger }e^{i\left( \Delta \tilde{ v}_{i,i^{\prime }}^{\left( +\right) \left( \alpha ,\alpha ^{\prime }\right) }+2\omega _{eg}\right) t}\right) +H.c. \end{aligned}$$

where we have defined a new symbol for difference between electronic and vibrational frequency $\tilde{v}_{i^{\prime },eg}^{\left( \pm \right) \left( \alpha ^{\prime }\right) }=\omega _{eg}\pm \tilde{v}_{i^{\prime }}^{\left( \alpha ^{\prime }\right) }$ and the vibrational frequency between different modes in the exponentials are defined as $\Delta \tilde{v} _{i,i^{\prime }}^{\left( \pm \right) \left( \alpha ,\alpha ^{\prime }\right) }=\tilde{v}_{i}^{\left( \alpha \right) }\pm \tilde{v}_{i^{\prime }}^{\left( \alpha ^{\prime }\right) }$.

The decay constants are derived in “Appendix 4”

60 $$\begin{aligned} \tilde{\Gamma }_{\left\{ \cdots \right\} }^{\left( *\right) \pm }&= {} \int d^{3}k~D\left( \mathbf {k}\right) \xi _{\mathbf {k},i^{\prime }}^{\left( eg\right) \left( \alpha ^{\prime }\right) }\xi _{\mathbf {k},i}^{\left( eg\right) \left( \alpha \right) }\pi \delta \left( \omega _{k}-\tilde{v} ^{(\pm )}\right) \left\{ \cdots \right\} _{\mathbf {k}} \nonumber \\&= {} \frac{1}{2}\frac{v^{(\pm )3}\left| \mathbf {M}_{i}^{\left( eg\right) \alpha }\right| \left| \mathbf {M}_{i^{\prime }}^{\left( eg\right) \alpha ^{\prime }}\right| }{3\pi \varepsilon _{0}\hbar c^{3}}\left\{ \cdots \right\} _{\tilde{v}^{(\pm )}} \end{aligned}$$

where $\tilde{v}^{(\pm )}=\left\{ \tilde{v}_{i^{\prime },eg}^{\left( +\right) \left( \alpha ^{\prime }\right) },\tilde{v}_{i^{\prime },eg}^{\left( -\right) \left( \alpha ^{\prime }\right) }\right\}$. *In the case of diatomic molecule we have checked that this Liouvillean reduces to the correct form by dropping the i index. *The rapid time oscillating terms in the last two lines containing $2\omega _{eg}$ are due to non-energy conserving counter-rotating terms in the Hamiltonian.

Similarly, the terms with $\tilde{\Gamma }$ evaluated at $\tilde{v} _{i^{\prime },eg}^{\left( -\right) \left( \alpha ^{\prime }\right) }$ is given by

61 $$\begin{aligned} \mathscr {L}_{\mathrm {el-vib-rad}}^{\left( *\right) \left( -\right) }&= {} - \tilde{\Gamma }_{\left\{ n+1\right\} }^{\left( *\right) -}\left( \left( \tilde{\rho }_{S}\left( t\right) C_{\alpha ^{\prime }i^{\prime }}C_{\alpha i}^{\dagger }-C_{\alpha i}^{\dagger }\tilde{\rho }_{S}\left( t\right) C_{\alpha ^{\prime }i^{\prime }}\right) e^{i\Delta \tilde{v}_{i,i^{\prime }}^{\left( -\right) \left( \alpha ,\alpha ^{\prime }\right) }t}+\left( B_{\alpha i}^{\dagger }C_{\alpha ^{\prime }i^{\prime }}^{\dagger }\tilde{\rho }_{S}\left( t\right) -C_{\alpha ^{\prime }i^{\prime }}^{\dagger }\tilde{\rho } _{S}\left( t\right) B_{\alpha i}^{\dagger }\right) e^{i\Delta \tilde{v} _{i,i^{\prime }}^{\left( +\right) \left( \alpha ,\alpha ^{\prime }\right) }t}\right. \nonumber \\&\left. \quad -\, B_{\alpha i}^{\dagger }\tilde{\rho }_{S}\left( t\right) C_{\alpha ^{\prime }i^{\prime }}e^{i\left( \Delta \tilde{v} _{i,i^{\prime }}^{\left( -\right) \left( \alpha ,\alpha ^{\prime }\right) }+2\omega _{eg}\right) t}-C_{\alpha ^{\prime }i^{\prime }}^{\dagger }\tilde{ \rho }_{S}\left( t\right) C_{\alpha i}^{\dagger }e^{i\left( \Delta \tilde{v} _{i,i^{\prime }}^{\left( +\right) \left( \alpha ,\alpha ^{\prime }\right) }-2\omega _{eg}\right) t}\right) \nonumber \\&\quad -\, \tilde{\Gamma }_{\left\{ n\right\} }^{\left( *\right) -}\left( \left( C_{\alpha i}^{\dagger }C_{\alpha ^{\prime }i^{\prime }}\tilde{\rho } _{S}\left( t\right) -C_{\alpha ^{\prime }i^{\prime }}\tilde{\rho }_{S}\left( t\right) C_{\alpha i}^{\dagger }\right) e^{i\Delta \tilde{v}_{i,i^{\prime }}^{\left( -\right) \left( \alpha ,\alpha ^{\prime }\right) }t}\left( \tilde{ \rho }_{S}\left( t\right) C_{\alpha ^{\prime }i^{\prime }}^{\dagger }B_{\alpha i}^{\dagger }-B_{\alpha i}^{\dagger }\tilde{\rho }_{S}\left( t\right) C_{\alpha ^{\prime }i^{\prime }}^{\dagger }\right) e^{i\Delta \tilde{v}_{i,i^{\prime }}^{\left( +\right) \left( \alpha ,\alpha ^{\prime }\right) }t}\right. \nonumber \\&\left. \quad -\, C_{\alpha i}^{\dagger }\tilde{\rho }_{S}\left( t\right) C_{\alpha ^{\prime }i^{\prime }}^{\dagger }e^{i\left( \Delta \tilde{ v}_{i,i^{\prime }}^{\left( +\right) \left( \alpha ,\alpha ^{\prime }\right) }-2\omega _{eg}\right) t}-C_{\alpha ^{\prime }i^{\prime }}\tilde{\rho } _{S}\left( t\right) B_{\alpha i}^{\dagger }e^{i\left( \Delta \tilde{v} _{i,i^{\prime }}^{\left( -\right) \left( \alpha ,\alpha ^{\prime }\right) }+2\omega _{eg}\right) t}\right) +H.c.\text {.} \end{aligned}$$

The terms correspond to the energy conserving processes that are illustrated in LHS of Fig. 1b. In the case when a single mode diatomic molecule is considered, the summation of index *i* is dropped, the exponentials become unity and the first and fifth lines of both Eqs. (59) and (61) in the two Liouvilleans reduce to the usual Lindblad form.

The terms $\left( B_{\alpha i}^{\dagger }B_{\alpha ^{\prime }i^{\prime }} \tilde{\rho }_{S}\left( t\right) -B_{\alpha ^{\prime }i^{\prime }}\tilde{\rho } _{S}\left( t\right) B_{\alpha i}^{\dagger }\right) \rightarrow b_{i}^{\left( \alpha \right) \dagger }b_{i}^{\left( \alpha \right) }\sigma _{ee}\tilde{\rho }_{S}-b_{i}^{\left( \alpha \right) }\sigma \tilde{\rho }_{S}\left( t\right) \sigma ^{\dagger }b_{i}^{\left( \alpha \right) \dagger }$ in the first line corresponding to $\mathscr {L}_{ee}=-b_{i}^{\left( \alpha \right) \dagger }b_{i}^{\left( \alpha \right) }\tilde{\rho }_{ee}$, $\mathscr {L} _{gg}=b_{i}^{\left( \alpha \right) }\tilde{\rho }_{gg}b_{i}^{\left( \alpha \right) \dagger }$ (i.e. terms with diagonal elements $\langle n|\tilde{\rho } _{\alpha \alpha }|n\rangle$). The second line $\tilde{\rho }_{S}\left( t\right) B_{\alpha ^{\prime }i^{\prime }}^{\dagger }C_{\alpha i}^{\dagger }-C_{\alpha i}^{\dagger }\tilde{\rho }_{S}\left( t\right) B_{\alpha ^{\prime }i^{\prime }}^{\dagger }\rightarrow \tilde{\rho }_{S}\left( t\right) \sigma _{ee}b_{i}^{\left( \alpha \right) \dagger }b_{i}^{\left( \alpha \right) \dagger }-|g\rangle b_{i}^{\left( \alpha \right) \dagger }\tilde{\rho } _{ee}\left( t\right) b_{i}^{\left( \alpha \right) \dagger }\langle g|$ gives off-diagonal elements difference by 2 vibrational quantas $\langle n|\tilde{ \rho }_{\alpha \alpha }|n\pm 2\rangle$.

### Liouvilleans of crossed terms with radiation bath

Finally, the method we introduced in “Appendix 3” can also be applied to obtain the crossed-term Liouvillean

62 $$\begin{aligned} \mathscr {L}_{\mathrm {\{el\times (el-vib)\}-rad}}&= {} \mathscr {L}_{\mathrm { (el\times el-vib)-rad}}+\mathscr {L}_{\mathrm {(el-vib\times el)-rad}} \nonumber \\&= {} \sum _{\alpha ,i}\left( \mathscr {L}_{\mathrm {(el\times el-vib)-rad} }^{\left( \alpha ,i\right) }+\mathscr {L}_{\mathrm {(el-vib\times el)-rad} }^{\left( \alpha ,i\right) }\right) \text {.} \end{aligned}$$

For the first term in Eq. (62), we have

63 $$\begin{aligned} \mathscr {L}_{\mathrm {(el\times el-vib)-rad}}^{\left( \alpha ,.i\right) }= \mathscr {L}_{\mathrm {(el\times el-vib)-rad}}^{\left( \alpha ,.i\right) \left( +\right) }+\mathscr {L}_{\mathrm {(el\times el-vib)-rad}}^{\left( \alpha ,i\right) \left( -\right) }+\Lambda _{\mathrm {(el\times el-vib)-rad} }^{\left( \alpha ,i\right) }\text {.} \end{aligned}$$

The Lamb shift term $\Lambda _{\mathrm {(el\times el-vib)-rad}}^{\left( \alpha ,i\right) }$ is given by Eq. (139) and the corresponding real parts are

64 $$\begin{aligned} \mathscr {L}_{\mathrm {(el\times el-vib)-rad}}^{\left( \alpha ,i\right) \left( +\right) }&= {} -\hat{\Gamma }_{\left\{ n+1\right\} }^{\left( \alpha ,i\right) +}\left( \left( \tilde{\rho }_{S}\left( t\right) B_{\alpha i}^{\dagger }\sigma -\sigma \tilde{\rho }_{S}\left( t\right) B_{\alpha i}^{\dagger }\right) e^{i\tilde{v}_{i}^{\left( \alpha \right) }t}-\sigma ^{\dagger } \tilde{\rho }_{S}\left( t\right) B_{\alpha i}^{\dagger }e^{i\left( \tilde{v} _{i}^{\left( \alpha \right) }+2\omega _{eg}\right) t}\right) \nonumber \\&\quad -\, \hat{\Gamma }_{\left\{ n\right\} }^{\left( \alpha ,i\right) +}\left( \left( \sigma B_{\alpha i}^{\dagger }\tilde{\rho }_{S}\left( t\right) -B_{\alpha i^{\prime }}^{\dagger }\tilde{\rho }_{S}\left( t\right) \sigma \right) e^{i\tilde{v}_{i}^{\left( \alpha \right) }t}-B_{\alpha i}^{\dagger } \tilde{\rho }_{S}\left( t\right) \sigma ^{\dagger }e^{i\left( \tilde{v} _{i}^{\left( \alpha \right) }+2\omega _{eg}\right) t}\right) +H.c. \end{aligned}$$

and

65 $$\begin{aligned} \mathscr {L}_{\mathrm {(el\times el-vib)-rad}}^{\left( \alpha ,i\right) \left( -\right) }&= {} -\hat{\Gamma }_{\left\{ n+1\right\} }^{\left( \alpha ,i\right) -}\left( \left( \sigma ^{\dagger }C_{\alpha i}^{\dagger }\tilde{\rho } _{S}\left( t\right) -C_{\alpha i}^{\dagger }\tilde{\rho }_{S}\left( t\right) \sigma ^{\dagger }\right) e^{i\tilde{v}_{i}^{\left( \alpha \right) }t}-C_{\alpha i}^{\dagger }\tilde{\rho }_{S}\left( t\right) \sigma e^{i\left( \tilde{v}_{i}^{\left( \alpha \right) }-2\omega _{eg}\right) t}\right) \nonumber \\&\quad -\, \hat{\Gamma }_{\left\{ n\right\} }^{\left( \alpha ,i\right) -}\left( \left( \tilde{\rho }_{S}\left( t\right) C_{\alpha i}^{\dagger }\sigma ^{\dagger }-\sigma ^{\dagger }\tilde{\rho }_{S}\left( t\right) C_{\alpha i}^{\dagger }\right) e^{i\tilde{v}_{i}^{\left( \alpha \right) }t}-\sigma \tilde{\rho }_{S}\left( t\right) C_{\alpha i}^{\dagger }e^{i\left( \tilde{v} _{i}^{\left( \alpha \right) }-2\omega _{eg}\right) t}\right) +H.c. \end{aligned}$$

where the decay constants are defined as

66 $$\begin{aligned} \hat{\Gamma }_{\left\{ \cdots \right\} }^{\left( \alpha ,i\right) \pm }\left( v\right)&= {} \sum _{i^{\prime }}\int d^{3}k~D\left( \mathbf {k}\right) \xi _{ \mathbf {k},i}^{\left( eg\right) \left( \alpha \right) }\varsigma _{i^{\prime },\mathbf {k}}^{\left( ge\right) }\pi \delta \left( \omega -\hat{v}^{(\pm )}\right) \left\{ \cdots \right\} _{\mathbf {k}} \nonumber \\&= {} \sum _{i^{\prime }}\frac{1}{2}\frac{\hat{v}^{(\pm )3}\left| \mathbf {M} _{i^{\prime }}^{\left( eg\right) 0}\right| \left| \mathbf {M} _{i}^{\left( eg\right) \alpha }\right| }{3\pi \varepsilon _{0}\hbar c^{3} }\left\{ \cdots \right\} _{\hat{v}^{(\pm )}} \end{aligned}$$

with $\hat{v}^{(\pm )}=\left\{ \tilde{v}_{i^{\prime },eg}^{\left( +\right) \left( \alpha \right) },\tilde{v}_{i^{\prime },eg}^{\left( -\right) \left( \alpha \right) }\right\}$. Similarly, for the the second term in Eq. (62), we have

67 $$\begin{aligned} \mathscr {L}_{\mathrm {(el-vib\times el)-rad}}^{\left( \alpha ,i\right) }= \mathscr {L}_{\mathrm {(el-vib\times el)-rad}}^{\left( \alpha ,i\right) \left( +\right) }+\mathscr {L}_{\mathrm {(el-vib\times el)-rad}}^{\left( \alpha ,i\right) \left( -\right) }+\Lambda _{\mathrm {(el-vib\times el)-rad} }^{\left( \alpha ,i\right) }\text {.} \end{aligned}$$

The Lamb shift term $\Lambda _{\mathrm {(el-vib\times el)-rad}}^{\left( \alpha ,i\right) }$ is given by Eq. (142) and the corresponding real parts are

68 $$\begin{aligned} \mathscr {L}_{\mathrm {(el-vib\times el)-rad}}^{\left( \alpha ,i\right) \left( +\right) }&= {} -\check{\Gamma }_{\left\{ n+1\right\} }^{\left( \alpha ,i\right) +}\left( \tilde{\rho }_{S}\left( t\right) \sigma ^{\dagger }C_{\alpha i}^{\dagger }-C_{\alpha i}^{\dagger }\tilde{\rho }_{S}\left( t\right) \sigma ^{\dagger }\right) e^{i\tilde{v}_{i}^{\left( \alpha \right) }t} \nonumber \\&-\check{\Gamma }_{\left\{ n\right\} }^{\left( \alpha ,i\right) +}\left( C_{\alpha i}^{\dagger }\sigma ^{\dagger }\tilde{\rho }_{S}\left( t\right) -\sigma ^{\dagger }\tilde{\rho }_{S}\left( t\right) C_{\alpha i}^{\dagger }\right) e^{i\tilde{v}_{i}^{\left( \alpha \right) }t}+H.c. \end{aligned}$$

and

69 $$\begin{aligned} \mathscr {L}_{\mathrm {(el-vib\times el)-rad}}^{\left( \alpha ,i\right) \left( -\right) }&= {} -\check{\Gamma }_{\left\{ n+1\right\} }^{\left( \alpha ,i\right) -}\left( B_{\alpha i}^{\dagger }\sigma \tilde{\rho }_{S}\left( t\right) -\sigma \tilde{\rho }_{S}\left( t\right) B_{\alpha i}^{\dagger }\right) e^{i\tilde{v}_{i}^{\left( \alpha \right) }t} \nonumber \\&-\check{\Gamma }_{\left\{ n\right\} }^{\left( \alpha ,i\right) -}\left( \tilde{\rho }_{S}\left( t\right) \sigma B_{\alpha i}^{\dagger }-B_{\alpha i}^{\dagger }\tilde{\rho }_{S}\left( t\right) \sigma \right) e^{i \tilde{v}_{i}^{\left( \alpha \right) }t}+H.c.\text {.} \end{aligned}$$

The decay constants are defined as

70 $$\begin{aligned} \check{\Gamma }_{\left\{ \cdots \right\} }^{\left( \alpha ,i\right) \pm }\left( v\right)&= {} \sum _{i^{\prime }}\int d^{3}k~D\left( \mathbf {k}\right) \xi _{\mathbf {k},i}^{\left( eg\right) \left( \alpha \right) }\varsigma _{i^{\prime },\mathbf {k}}^{\left( ge\right) }\pi \delta \left( \omega _{k}- \check{v}^{(\pm )}\right) \left\{ \cdots \right\} _{\mathbf {k}} \nonumber \\&= {} \sum _{i^{\prime }}\frac{1}{2}\frac{\check{v}^{(\pm )3}\left| \mathbf {M }_{i}^{\left( eg\right) \alpha }\right| \left| \mathbf {M}_{i^{\prime }}^{\left( eg\right) 0}\right| }{3\varepsilon _{0}\pi \hbar c^{3}} \left\{ \cdots \right\} _{\check{v}^{(\pm )}} \end{aligned}$$

where $\check{v}^{(\pm )}=\left\{ \tilde{v}_{i,eg}^{\left( +\right) \left( \alpha \right) },\tilde{v}_{i,eg}^{\left( -\right) \left( \alpha \right) }\right\}$. Note that $\hat{\Gamma }_{\left\{ \cdots \right\} }^{\left( \alpha ,i\right) \pm }=\check{\Gamma }_{\left\{ \cdots \right\} }^{\left( \alpha ,i\right) \pm }$.

## Two-time correlation functions

This section is devoted to derive the formula for absorption and emission specturm of a molecule from the two-time correlation function. In general the polarization $\mathbf {P}$ will be a nonlinear functional of the electric field $\mathbf {E}$, however we will restrict ourselves to discuss the simplest case of a linear relationship $\mathbf {P=}\chi \mathbf {E}$ which means the response of the molecular system is completely determined by the linear electric susceptibility $\chi$. According to (Maxwell’s propagation equation, Beer’s law), the absorption coefficient of a medium is given by the imaginary component of the its wave number $k_{med}=\omega \sqrt{ \varepsilon }/c=k_{R}+ik_{I}$,

71 $$\begin{aligned} \alpha \left( \omega \right) =2k_{I}=\frac{2\omega }{c}\text {Im}\left( \sqrt{ \varepsilon }\right) =\frac{2\omega }{c}\text {Im}\left( \sqrt{1+\chi } \right) \simeq \frac{\omega }{c}\text {Im}\chi \left( \omega \right) \end{aligned}$$

To connect $\varepsilon \left( \omega \right)$ with dipole correlation, we start with Liouville-von Neumann of the form

72 $$\begin{aligned} \frac{\partial \rho }{\partial t}=\left( \frac{1}{i\hbar }\right) \left[ -\sum \limits _{\alpha }\mu _{\alpha }\left( t\right) \mathscr {E}_{\alpha }\left( t\right) ,\rho \right] \text {.} \end{aligned}$$

Formally integrating this leads to

73 $$\begin{aligned} \rho (t)&= {} \rho (0)+\left( \frac{1}{i\hbar }\right) [-\sum \limits _{\alpha }\int _{0}^{t}\mu _{\alpha }\left( t^{\prime }\right) \mathscr {E}_{\alpha }\left( t^{\prime }\right) ,\rho \left( t^{\prime }\right) ]dt^{\prime }=\sum _{n=1}^{\infty }\rho ^{\left( n\right) }(t) \nonumber \\&= {} \rho (0)+\frac{1}{i\hbar }[-\sum \limits _{\alpha }\int _{0}^{t}\mu _{\alpha }\left( t^{\prime }\right) \mathscr {E}_{\alpha }\left( t^{\prime }\right) ,\rho \left( 0\right) ]dt^{\prime }-\frac{1}{\hbar ^{2}}[\sum \limits _{\alpha }\int _{0}^{t}\mu _{\alpha }\left( t^{\prime }\right) \mathscr {E}_{\alpha }\left( t^{\prime }\right) ,[\sum \limits _{\beta }\int _{0}^{t}\mu _{\beta }\left( t^{\prime \prime }\right) \mathscr {E}_{\beta }\left( t^{\prime \prime }\right) ,\rho \left( t^{\prime \prime }\right) ]dt^{\prime \prime }]dt^{\prime }\ldots \end{aligned}$$

In general we may write

74 $$\begin{aligned} \rho ^{\left( n\right) }\left( t\right)&= {} \left( \frac{i}{\hbar }\right) ^{n}\int _{0}^{\infty }d\tau _{n}\int _{0}^{\infty }d\tau _{n-1}\dots \int _{0}^{\infty }d\tau _{1}\mathscr {E}\left( t-\tau _{n}\right) \mathscr {E} \left( t-\tau _{n}-\tau _{n-1}\right) \dots \mathscr {E}\left( t-\tau _{n}-\dots -\tau _{1}\right) \nonumber \\&\times \left[ \mu \left( -\tau _{n}\right) ,\left[ \mu \left( -\tau _{n}-\tau _{n-1}\right) ,\dots ,\left[ \mu \left( -\tau _{n}-\tau _{n-1}\dots -\tau _{1}\right) ,\rho ^{\left( eq\right) }\right] \dots \right] \right] \end{aligned}$$

where $\mu \left( t\right) =e^{iHt/\hbar }\mu e^{-iHt/\hbar }$ is the dipole operator given in the Heisenberg picture (i.e. *H* contains the thermal environment and the interaction terms).

The molecular response pertaining to its optical properties is the dipole induced in the molecule, that can be calculated from the expectation value of the dipole moment and polarization

75 $$\begin{aligned} \left\langle P_{q}\left( r,t\right) \right\rangle&= {} \sum _{n=1}^{\infty }\left\langle P_{q}\left( r,t\right) \right\rangle ^{\left( n\right) }=N\left\langle \mu _{q}\left( t\right) \right\rangle \text {,} \end{aligned}$$

76 $$\begin{aligned} \left\langle \mu _{q}\left( t\right) \right\rangle&= {} Tr\left( \mu _{q}\left( t\right) \rho (t)\right) =\sum _{n=0}^{\infty }\left\langle \mu _{q}\left( t\right) \right\rangle ^{\left( n\right) }=\sum _{n=0}^{\infty }Tr\left( \mu _{q}\left( t\right) \rho ^{\left( n\right) }\right) \end{aligned}$$

where

77 $$\begin{aligned} \left\langle \mu _{q}\left( t\right) \right\rangle ^{\left( 0\right) }&= {} Tr\left( \mu _{q}\left( t\right) \rho ^{\left( 0\right) }\right) =Tr\left( \mu _{q}\left( t\right) \rho ^{\left( eq\right) }\right) \text {,} \end{aligned}$$

78 $$\begin{aligned} \left\langle \mu _{q}\left( t\right) \right\rangle ^{\left( 1\right) }&= {} Tr\left( \mu _{q}\left( t\right) \rho ^{\left( 1\right) }\right) =-\left( \frac{1}{i\hbar }\right) \sum \limits _{\alpha }\int _{0}^{t}Tr\left( \mu _{q}\left( t\right) [\mu _{\alpha }\left( t^{\prime }\right) ,\rho \left( 0\right) ]\mathscr {E}_{\alpha }\left( t^{\prime }\right) dt^{\prime }\right) \text {.} \end{aligned}$$

From the linear polarization

79 $$\begin{aligned} \left\langle P_{q}\left( r,t\right) \right\rangle ^{\left( 1\right) }&= {} -N\left( \frac{1}{i\hbar }\right) \sum \limits _{\alpha }\int _{0}^{t}Tr\left( \mu _{q}\left( t\right) [\mu _{\alpha }\left( t^{\prime }\right) ,\rho \left( 0\right) ]\mathscr {E}_{\alpha }\left( t^{\prime }\right) dt^{\prime }\right) \nonumber \\&= {} \varepsilon _{0}\sum \limits _{\alpha }\int _{0}^{t}\chi _{q\alpha }^{\left( 1\right) }\left( r;t,t^{\prime }\right) \mathscr {E}_{\alpha }\left( t^{\prime }\right) dt^{\prime }\text {,} \end{aligned}$$

we have the susceptibility tensor

80 $$\begin{aligned} \chi _{q\alpha }^{\left( 1\right) }\left( r;t,t^{\prime }\right)&= {} -N\left( \frac{1}{i\hbar }\right) Tr\left( \mu _{q}\left( t\right) [\mu _{\alpha }\left( t^{\prime }\right) ,\rho \left( 0\right) ]\right) ,t^{\prime }=t-\tau \end{aligned}$$

81 $$\begin{aligned} \chi _{q\alpha }^{\left( 1\right) }\left( \omega \right)&= {} \int _{-\infty }^{\infty }\chi _{q\alpha }^{\left( 1\right) }(t-t^{\prime })e^{i\omega (t-t^{\prime })}dt^{\prime }=-N\left( \frac{1}{i\hbar }\right) \int _{-\infty }^{\infty }Tr\left( \mu _{q}\left( t\right) [\mu _{\alpha }\left( t-\tau \right) ,|g\rangle \langle g|\rho _{vib}]\right) e^{i\omega \tau }d\tau \nonumber \\&= {} -N\left( \frac{1}{i\hbar }\right) \int _{-\infty }^{\infty }Tr[\mu _{q}\left( t\right) \mu _{\alpha }\left( t-\tau \right) \rho \left( 0\right) -\mu _{q}\left( t\right) \rho \left( 0\right) \mu _{\alpha }\left( t-\tau \right) ]e^{i\omega \tau }d\tau \nonumber \\&= {} -N\left( \frac{1}{i\hbar }\right) \int _{-\infty }^{\infty }[\left\langle \mu _{q}\left( t\right) \mu _{\alpha }\left( t-\tau \right) \right\rangle -\left\langle \mu _{\alpha }\left( t-\tau \right) \mu _{q}\left( t\right) \right\rangle ]e^{i\omega \tau }d\tau \nonumber \\&= {} -N\left( \frac{1}{i\hbar }\right) \int _{-\infty }^{\infty }C_{q\alpha }^{\left( 1\right) }\left( \tau \right) e^{i\omega \tau }d\tau =-N\left( \frac{1}{i\hbar }\right) 2i\int _{-\infty }^{\infty }\text {Im}\left\langle \mu _{q}\left( \tau \right) \mu _{\alpha }\left( 0\right) \right\rangle e^{i\omega \tau }d\tau \end{aligned}$$

where

82 $$\begin{aligned} C_{q\alpha }^{\left( 1\right) }\left( \tau \right)&= {} \left\langle [\mu _{q}\left( t\right) ,\mu _{\alpha }\left( t-\tau \right) ]\right\rangle \nonumber \\\rightarrow & {} \left\langle \mu _{q}\left( \tau \right) \mu _{\alpha }\left( 0\right) \right\rangle -\left\langle \mu _{\alpha }\left( 0\right) \mu _{q}\left( \tau \right) \right\rangle =A-A^{*}=2i\text {Im}A\text {.} \end{aligned}$$

Using the fact that $e^{iH^{\left( mol\right) }\tau /\hbar }\left| g\right\rangle =e^{i\left( \omega _{g}+H_{g}/\hbar \right) \tau }\left| g\right\rangle$ and $e^{iH\tau /\hbar }\left| e\right\rangle =e^{i\left( \omega _{e}+H_{e}/\hbar \right) \tau }\left| e\right\rangle$ where $H^{\left( mol\right) }=\sigma _{gg}\left( \hbar \omega _{g}+H_{g}\right) +\sigma _{ee}\left( \hbar \omega _{e}+H_{e}\right)$ is the pure molecular Hamiltonian (i.e. $H_{\mathrm {mol-ph}}$ but with phonon related terms dropped), we can write the molecular dipole moment as $\mu _{q}\left( t\right) =\mu _{q}^{eg}\left( \mathbf {u,}t\right) +\mu _{q}^{ge}\left( \mathbf {u,}t\right)$ with

83 $$\begin{aligned} \mu _{q}^{eg}\left( \mathbf {u,}t\right)&= {} \hat{M}_{q}^{eg}\left( \mathbf {u,} t\right) \hat{\sigma }_{+}\left( t\right) =e^{iHt}\hat{M}_{q}^{eg}|e\rangle \langle g|e^{-iHt}=e^{i\omega _{e}t}e^{iH_{e}t}\hat{M}_{q}^{eg}\left| e\right\rangle \left\langle g\right| e^{-i\omega _{g}t}e^{-iH_{g}t}\text { ,} \end{aligned}$$

84 $$\begin{aligned} \hat{\mu }_{q}^{ge}\left( \mathbf {u,}t\right)&= {} \hat{M}_{q}^{ge}\left( \mathbf {u,}t\right) \hat{\sigma }_{-}\left( t\right) =e^{iHt}\hat{M} _{q}^{ge}|g\rangle \langle e|e^{-iHt}=e^{i\omega _{g}t}e^{iH_{g}t}\hat{M} _{q}^{ge}\left| g\right\rangle \left\langle e\right| e^{-i\omega _{e}t}e^{-iH_{e}t}\text {.} \end{aligned}$$

The two-time correlation can be written as^41,42^

85a $$\begin{aligned} \left\langle \mu _{q}\left( t\right) \mu _{\alpha }\left( t-\tau \right) \right\rangle&= {} Tr\{\{\mu _{q}^{eg}\left( t\right) +\mu _{q}^{ge}\left( t\right) \}\{\mu _{\alpha }^{eg}\left( t^{\prime }\right) +\mu _{\alpha }^{ge}\left( t^{\prime }\right) \}\rho ^{th}\} \nonumber \\&= {} Tr_{vib}\{\langle g|\{\hat{M}_{q}^{eg}\left( t\right) \hat{\sigma } _{+}\left( t\right) +\hat{M}_{q}^{ge}\left( t\right) \hat{\sigma }_{-}\left( t\right) \}\{\hat{M}_{\alpha }^{eg}\left( t^{\prime }\right) \hat{\sigma } _{+}\left( t^{\prime }\right) +\hat{M}_{\alpha }^{ge}\left( t^{\prime }\right) \hat{\sigma }_{-}\left( t^{\prime }\right) \}|g\rangle \langle g|\rho _{vib}^{th}|g\rangle \} \nonumber \\&= {} Tr_{vib}\{\langle g|\{e^{i\omega _{g}t}e^{iH_{g}t}\hat{M} _{q}^{ge}\left| g\right\rangle \left\langle e\right| e^{-i\omega _{e}t}e^{-iH_{e}t}\}\{e^{i\omega _{e}t^{\prime }}e^{iH_{e}t^{\prime }}\hat{M} _{\alpha }^{eg}\left| e\right\rangle \left\langle g\right| e^{-i\omega _{g}t^{\prime }}e^{-iH_{g}t^{\prime }}\}|g\rangle \rho _{vib}^{th}\} \nonumber \\&= {} Tr_{vib}e^{i\omega _{g}t}e^{-i\omega _{e}t}e^{i\omega _{e}t^{\prime }}e^{-i\omega _{g}t^{\prime }}\{\{e^{iH_{g}t}\hat{M} _{q}^{ge}e^{-iH_{e}t}e^{iH_{e}t^{\prime }}\hat{M}_{\alpha }^{eg}e^{-iH_{g}t^{\prime }}\rho _{vib}^{th}\} \nonumber \\&= {} e^{-i\omega _{eg}\tau }Tr_{vib}\{\hat{M}_{q}^{ge}e^{-iH_{e}\tau }\hat{M} _{\alpha }^{eg}e^{iH_{g}\tau }\rho _{vib}^{th}\} \nonumber \\&= {} e^{-i\omega _{eg}\tau }Tr_{vib}\{e^{iH_{g}\tau }\hat{M} _{q}^{ge}e^{-iH_{e}\tau }\hat{M}_{\alpha }^{eg}\rho _{vib}^{th}\} \end{aligned}$$

where $\tau =t-t^{\prime }$ and we assume thermal equilibrium vibronic state $\rho ^{th}=|g\rangle \langle g|\rho _{vib}^{th}$ so the trace is over the $|g\rangle$ only.

### Absorption spectra

Hence, the absorption spectrum can now be related to the correlation function via the linear susceptibility as

86a $$\begin{aligned} \alpha \left( \omega \right)\simeq & {} \frac{\omega }{c}\text {Im}\chi _{q\alpha }^{\left( 1\right) }\left( \omega \right) =\frac{\omega }{c} N\left( \frac{1}{\hbar }\right) \text {Im}i\int _{-\infty }^{\infty }C_{q\alpha }^{\left( 1\right) }\left( \tau \right) e^{i\omega \tau }d\tau = \frac{2\omega N}{c\hbar }\text {Re}\int _{0}^{\infty }\left\langle [\mu _{q}\left( t\right) ,\mu _{\alpha }\left( t-\tau \right) ]\right\rangle e^{i\omega \tau }d\tau \nonumber \\&= {} \frac{2\omega N}{c\hbar }\text {Re}\int _{0}^{\infty }Tr_{vib}[\{\hat{M} _{q}^{ge}e^{-iH_{e}\tau }\hat{M}_{\alpha }^{eg}e^{iH_{g}\tau }\rho _{vib}^{th}\}-\{\hat{M}_{\alpha }^{ge}e^{iH_{e}\tau }\hat{M} _{q}^{eg}e^{-iH_{g}\tau }\rho _{vib}^{th}\}e^{i2\omega _{eg}\tau }]e^{i(\omega -\omega _{eg})\tau }d\tau \nonumber \\&= {} \frac{2\omega N}{c\hbar }\text {Re}\int _{0}^{\infty }Tr_{vib}\{\hat{M} _{q}^{ge}e^{-iH_{e}\tau }\hat{M}_{\alpha }^{eg}e^{iH_{g}\tau }\rho _{vib}^{th}\}e^{i(\omega -\omega _{eg})\tau }d\tau \text {.} \end{aligned}$$

For the second term $\left\langle \mu _{\alpha }\left( t-\tau \right) \mu _{q}\left( t\right) \right\rangle$ we interchange $\alpha$ and $q,t^{\prime }$ and *t* but it vanishes.

### Emission spectra

By Fourier transform of the correlation

87 $$\begin{aligned} \left\langle E^{\left( -\right) }\left( \mathbf {r},t\right) E^{\left( +\right) }\left( \mathbf {r},t+\tau \right) \right\rangle =I_{0}\left( \mathbf {r}\right) \left\langle \mu _{q}^{eg}\left( \mathbf {u,}t\right) \mu _{q}^{ge}\left( \mathbf {u,}t+\tau \right) \right\rangle \end{aligned}$$

with $I_{0}\left( \mathbf {r}\right) =\left( \frac{\omega ^{2}\sin \eta }{ 4\pi \varepsilon _{0}c^{2}\left| \mathbf {r-r}_{0}\right| }\right) ^{2}$ we have the emission spectra

88 $$\begin{aligned} S\left( \omega _{L}\right)&= {} \frac{1}{\pi }\Re \int _{0}^{\infty }d\tau \left\langle E^{\left( -\right) }\left( \mathbf {r},t\right) E^{\left( +\right) }\left( \mathbf {r},t+\tau \right) \right\rangle e^{i\omega _{L}\tau } \nonumber \\&= {} \frac{1}{\pi }I_{0}\left( \mathbf {r}\right) \Re \int _{0}^{\infty }d\tau \sum _{v^{\prime \prime }}\left\langle v^{\prime \prime }\right| \left[ \hat{M}_{q}^{eg}e^{iH_{g}\tau }\hat{M}_{q}^{ge}e^{-iH_{e}\tau }\rho _{e}^{th} \right] \left| v^{\prime \prime }\right\rangle e^{i(\omega _{L}-\omega _{eg})\tau } \end{aligned}$$

where

89 $$\begin{aligned} \left\langle \hat{\mu }_{eg}\left( \mathbf {u,}t\right) \hat{\mu }_{ge}\left( t+\tau \right) \right\rangle&= {} \left\langle \{e^{iHt}\hat{M}_{q}^{eg}\sigma _{+}(0)e^{-iHt}\}\{e^{iH(t+\tau )}\hat{M}_{q}^{ge}\sigma _{-}(0)e^{-iH(t+\tau )}\}\right\rangle _{ss} \nonumber \\&= {} Tr_{e}\left[ e^{iHt}\hat{M}_{q}^{eg}\left| e\right\rangle \left\langle g\right| e^{-iHt}e^{iH(t+\tau )}\hat{M}_{q}^{ge}\left| g\right\rangle \left\langle e\right| e^{-iH(t+\tau )}\rho _{e}^{th} \right] e^{i\omega _{L}\tau } \nonumber \\&= {} \sum _{v^{\prime \prime }}\left\langle e,v^{\prime \prime }\right| \left[ \begin{array}{c} e^{i\omega _{e}t}e^{iH_{e}t}\hat{M}_{q}^{eg}\left| e\right\rangle \left\langle g\right| e^{-i\omega _{g}t}e^{-iH_{g}t}e^{i\omega _{g}(t+\tau )}e^{iH_{g}(t+\tau )} \\ \times \hat{M}_{q}^{ge}\left| g\right\rangle \left\langle e\right| e^{-i\omega _{e}(t+\tau )}e^{-iH_{e}(t+\tau )}\rho _{e}^{th} \end{array}\right] \left| e,v^{\prime \prime }\right\rangle \nonumber \\&= {} \sum _{v^{\prime \prime }}\left\langle v^{\prime \prime }\right| \left[ e^{iH_{e}t}\hat{M}_{q}^{eg}e^{-iH_{g}t}e^{iH_{g}(t+\tau )}\hat{M} _{q}^{ge}e^{-iH_{e}(t+\tau )}\rho _{e}^{th}\right] \left| v^{\prime \prime }\right\rangle e^{-i\omega _{eg}(\tau )} \nonumber \\&= {} \sum _{v^{\prime \prime }}\left\langle v^{\prime \prime }\right| \left[ \hat{M}_{q}^{eg}e^{iH_{g}\tau }\hat{M}_{q}^{ge}e^{-iH_{e}(t+\tau )}\rho _{e}^{th}e^{iH_{e}t}\right] \left| v^{\prime \prime }\right\rangle e^{-i\omega _{eg}(\tau )}\text {.} \end{aligned}$$

**Figure 2:**
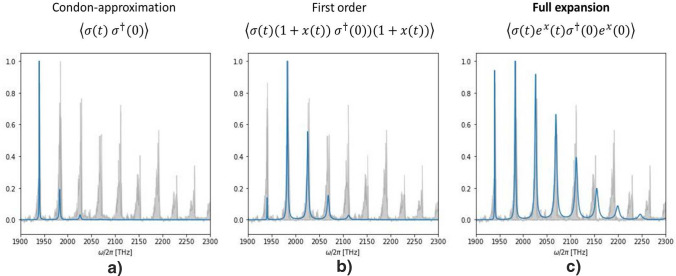
Absorption lineshape of carbon monoxide at 303 K. Blue-solid lines show theoretical model and grey background shows experimental data obtained from^43^ (digitalized). The dipole matrix elements $\mathbf {M}^{eg}$ and $\mathbf {M}^{ge}$ are expanded with respect to the nuclear coordinates *x* in different orders: (**a**) Condon-approximation (**b**) Truncated at first-order (Herzberg–Teller) term (**c**) full exponential. The parameters: $\omega _{eg}=2\pi \times 1940\,\mathrm{THz}$, $v^{\left( g\right) }=v^{\left( e\right) }=2\pi \times 43\,\mathrm{THz}$, $s=0.62\times 10^{-11}$ m, $\omega _{c}=2\pi \times 5.7THz$, $\Gamma =10^{9}\,\mathrm{GHz}$ and $m=1.1\times 10^{-26}$ kg.

**Figure 3:**
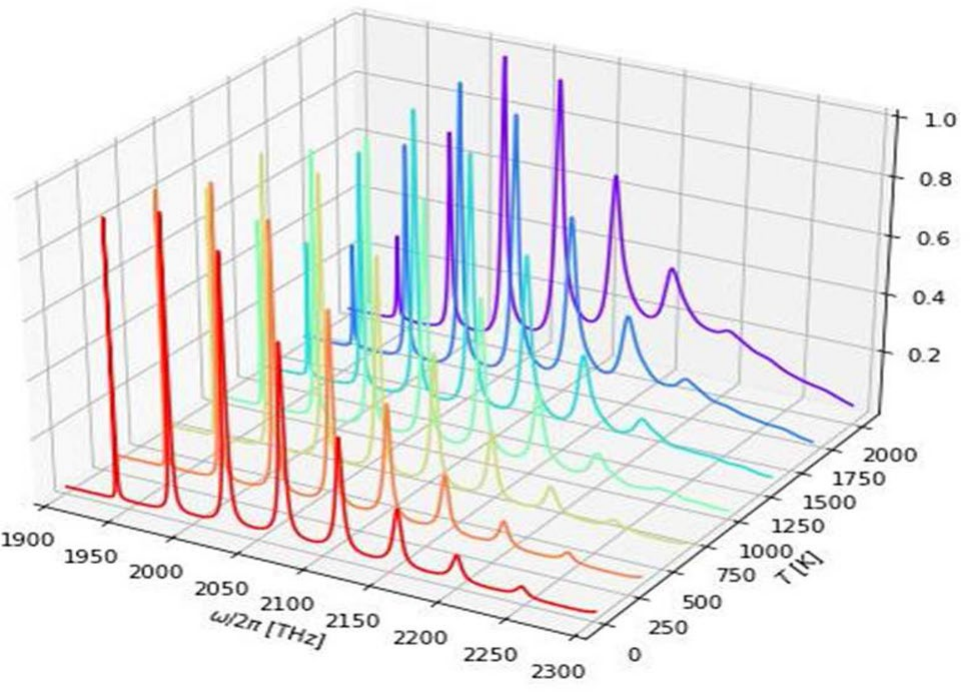
Absorption lineshape of carbon monoxide with varying temperature (y-axis) in the case when $\mathbf {M}^{eg}$ and $\mathbf {M}^{ge}$ are expanded fully. The same parameters as in Fig. 2 were used.

**Figure 4:**
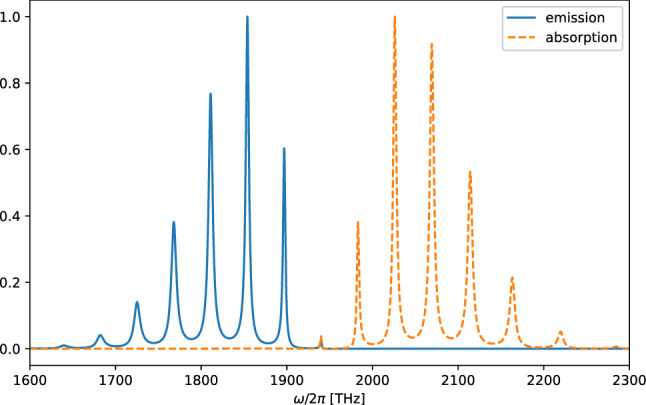
Emission lineshape (solid blue line) versus absorption lineshape (orange dotted line) of carbon monoxide. A breaking in the mirror symmetry is observed. Both the emission and absorption lineshapes are plotted using the same set of parameters as in Fig. 2.

**Figure 5:**
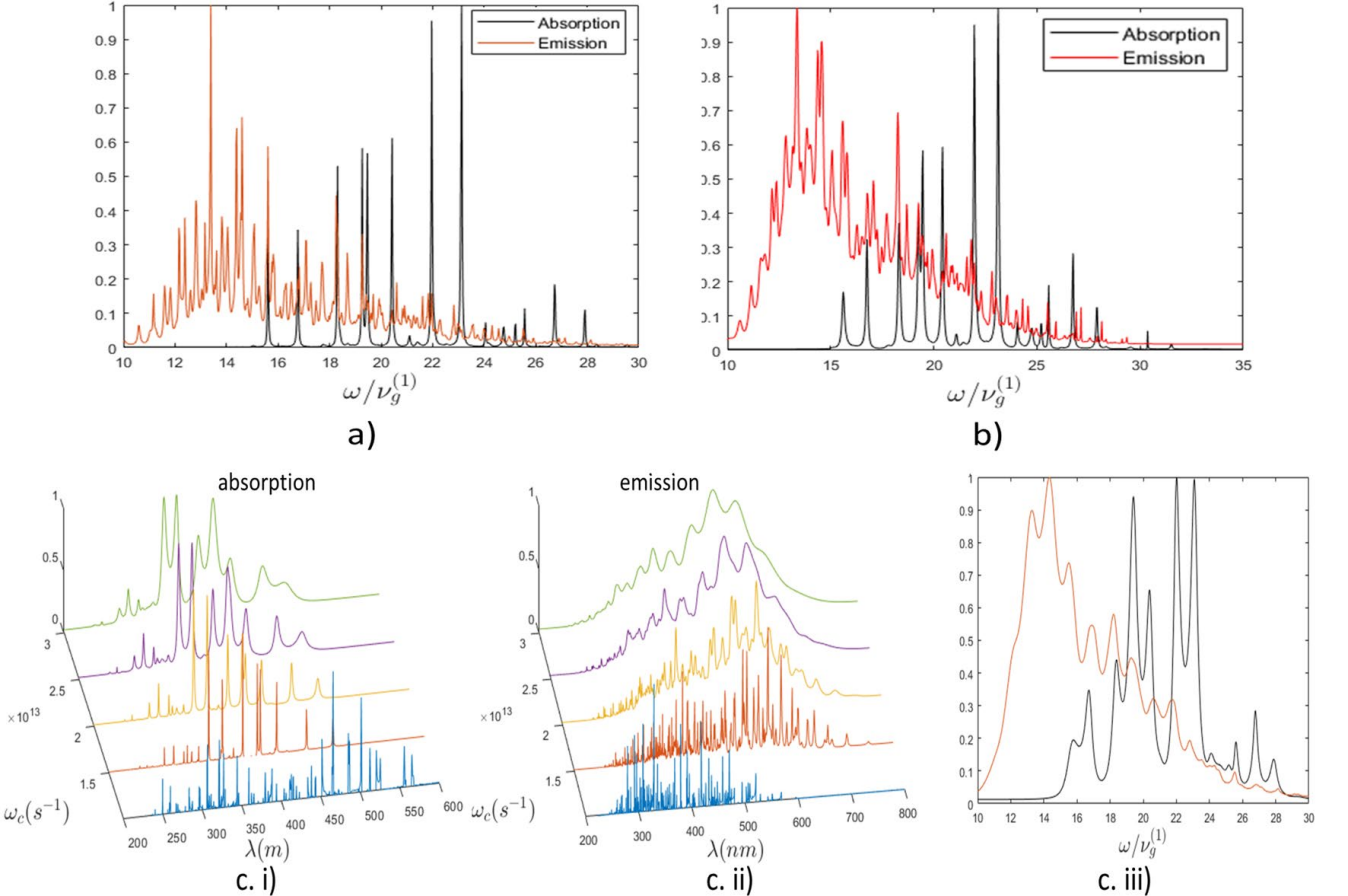
Emission lineshape (solid blue line) versus absorption lineshape (orange dotted line) of nitrogen dioxide. Liouvillean includes: (**a**) electronic-radiation and vibrational dampings, (**b**) electronic-radiation, electronic-phonon and vibrational dampings, with cutoff frequency $$\omega _{c}=2\pi 3.2\times 10^{12}$$s$$^{-1}$$, (**c**) electronic-radiation, electronic-vibrational-radiation, cross-term, electronic-phonon and vibrational dampings for (i) absorption, (ii) emission spectra versus wavelengths for several cutoff frequencies $$\omega_{c}=2\protect\pi[1.6,2.4,3.2,4.0,4.8]\times 10^{12}\rm{s}^{-1}$$, (iii) spectra for the largest $$\omega_{c}$$. We use 3 vibrational levels and 3 modes. with displacements $$ s_{i}=10^{-11}$$m, $$\nu _{i}^{(g)}$$ = $$2\pi [1322.5,750.9,1616.0]$$cm$$^{-1},$$ $$\omega ^{(e)}=2\pi 28000$$ cm $$^{-1}$$.

## Results and discussion

Although our theory is general for typical polyatomic molecules and unifies the main dissipative mechanisms, we only focus on a simple diatomic molecule in this work and the analysis for polyatomic molecule will be included in a future work. For a diatomic molecule, there exists only a single vibrational mode and this means we can drop all the *i* indices and $\sum _{i,i^{\prime }}$ in the results derived in the preceding sections. The coherent internal coupling term $V_{vib-vib}$ also plays no role here. The results shown in this section are obtained from numerical simulation using QuTiP Python package^33^. The “cross-term Liouvillean” and counter-rotating terms derived in “Dynamics of the quantum system” are not included in the numerical simulation of this study but will be included in a future work.

### Absorption lineshape

Carbon monoxide (CO) has been chosen in this work because it has importance indirect effects on global warming making its experimental measured data readily available. In Fig. 2 we plotted the absorption spectrum of CO in comparison with experimental data measured by^43^ . The parameters we used are summarized in figure caption. We see that under the usual Condon approximation (Fig. 2a), we can only capture the ZPL (i.e. $\left| g,0\right\rangle \rightarrow \left| e,0\right\rangle$ transition) but failed to capture succeeding peaks for $\left| g,0\right\rangle \rightarrow \left| e,3\right\rangle ,\left| e,4\right\rangle ,\dots$ transition. On the other hand, if $\mathbf {M}^{\left( eg\right) }$ and $\mathbf {M}^{\left( ge\right) }$ are expanded to first order term (Herzberg–Teller) with respect to the vibrational coordinates *x* (Fig. 2b), one observe more peaks in the spectrum but the overall strength of the peaks are not in agreement with the experimental data. This is because each term in the expansion of $\mathbf {M}^{\left( eg\right) }$ (and $\mathbf {M}^{\left( ge\right) }$) would account for the strength of different peaks and interfere with each other. We see that only in the case when the dipole matrix elements $\mathbf {M}^{\left( eg\right) }$ and $\mathbf {M}^{\left( ge\right) }$ are expanded fully with respect to the nuclear coordinates, the absorption spectrum predicted by the theory (solid blue line) is consistent with the experimental data^43^(grey shadow background). This emphasizes the importance of non-Condon terms when molecular spectrum is considered. The inconsistency between the theoretical predicted peaks and the experimental measured peaks beyond 2150 *THz* is due to anharmonicity of the nuclear potentials of CO not included in our harmonic potential model. This highlights the limitations of harmonic approximation of the nuclear potentials even in a simple diatomic molecule.

### Temperature dependence

The absorption spectrum is also plotted with varying temperature in Fig. 3. We observe an inhomogeneous broadening due to the phonon bath which is typical for systems found in lattice or solid medium^44,45^. The physical picture behind this is as follows: as the temperature of the environment increases, multi-phonon absorption processes can take place more frequently such that the life-time of the ground vibronic states decreases while the absorption peaks become broadened. On the other hand, at higher temperature the zero phonon $\left| g,0\right\rangle \rightarrow \left| e,0\right\rangle$ transition becomes less likely to occur and we observe a drop in the intensity of the ZPL.

### Emission and absorption spectra

In Fig. 4 we plotted the emission spectrum (blue-solid line) in comparison with the absorption spectrum (orange-dash line) at $T=303K$ and the spectra are expanded to first-order non-Condon term (Herzberg–Teller). The same set of parameters as the previous figures are used. We observed violation of the mirror image symmetry as a consequence of taking into account the first-order non-Condon term (Herzberg–Teller) for the emission and absorption spectrum. This is also discussed in^46^ where a strong deviation from mirror image symmetry was observed in $\pi$ -conjugated, organic molecules. This again stress the importance of non-Condon effects when we further generalize our system to polyatomic molecule in the future work.

### Decay constants

Full derivation of the decay constants associated with Liouvilleans (cross terms and non-cross terms) can be found in the “Appendix 4”. The decay constants that are related with the radiation bath (i.e. $\Gamma ,~\tilde{\Gamma },~\hat{\Gamma }$ and $\check{\Gamma }$) are derived by following the common approach in quantum optics, one can perform the integration by changing to spherical coordinates in $\mathbf {k}$-space and introduce the photon density of states^34,47^. The spectra is highly dependent on the photon density of states $D\left( \mathbf {k}\right)$ and non-Markovian. Only in free space we can use $\ D\left( \mathbf {k}\right) d^{3}k\longrightarrow \frac{\omega ^{2}V }{8\pi ^{3}c^{3}}d\omega \sin \theta ~d\theta ~d\phi$ with Markov approximation

### Spectral density

However, there are still many problems where the functional form of the decay constants in the Liouvillean is not known, the coupling between the molecule and the phonon bath is one instance ($\Upsilon _{\left\{ \cdots \right\} }^{\left( \alpha ,\alpha ^{\prime },i,i^{\prime }\right) }$). In these cases, a phenomenological approach has been proposed by introducing the spectral density $J\left( \omega \right)$ and the physical motivation behind this can be found in^12,48,49^. It was shown by these authors that for any problem in which a thermal equilibrium statistical average is taken over the initial states of the environment and a sum over the final states, complete information about the effect of the environment is encapsulated in the single “spectral function” $J\left( \omega \right)$. This approach has since been applied in a variety of physical system studies where the coupling between a system (e.g. atom, molecule, crystal defect, electron) and acoustic phonon is considered. For our case, this means that we replace the integral over phonon density of states and coupling constants with

90 $$\begin{aligned}&\int d^{3}l~\digamma \left( \mathbf {l}\right) \Theta _{i\mathbf {l}}^{\left( \alpha \right) }\Theta _{i^{\prime }\mathbf {l}}^{\left( \alpha ^{\prime }\right) }..\longrightarrow \int d\omega ~_{\mathbf {l}}J_{i,i^{\prime }}^{\left( \alpha ,\alpha ^{\prime }\right) }\left( \omega _{\mathbf {l} }\right) ..=\pi J_{i,i^{\prime }}^{\left( \alpha ,\alpha ^{\prime }\right) }\left( \tilde{v}_{i^{\prime }}^{\left( \alpha ^{\prime }\right) }\right) \left\{ \cdots \right\} _{\tilde{v}_{i^{\prime }}^{\left( \alpha ^{\prime }\right) }} \end{aligned}$$

91 $$\begin{aligned}&\int d^{3}l~\digamma \left( \mathbf {l}\right) \Theta _{i\mathbf {l}}^{\left( \alpha \right) }\Theta _{i^{\prime }\mathbf {l}}^{\left( \alpha ^{\prime }\right) }\longrightarrow \int d\omega ~J\left( \omega \right) \end{aligned}$$

where we assumed the same spectral density $J\left( \omega \right)$ applies for different electronic state $\alpha$ and vibrational mode *i*. A common assumption is that the functional form of $J\left( \omega \right)$ is a reasonably smooth function of $\omega$^12^ and it is of the form $\omega ^{S}$ up to some cut-off frequency $\omega _{c}$

92 $$\begin{aligned} J\left( \omega \right) =\frac{\omega ^{S}}{\omega _{c}^{S-1}}f\left( \omega ,\omega _{c}\right) \end{aligned}$$

*A summary of different physical system that was modelled using spectral density of different S values can be found below. *There are three cases:

- $S=1$ is commonly referred to as the “Ohmic bath”: Mesoscopic metal ring^50^, Josephson-junction circuits^51^,
- $0<S<1$ as the “sub-Ohmic bath”: Mesoscopic ring^50^, Nano-electro-mechanical devices^52^.
- $S>1$ as the “super-Ohmic bath”: InGaAs/GaAs quantum dot^53^ , DBT-doped nanocrystal of anthracene^11^, SiV and NV centers in diamond^54^.

Here $f\left( \omega ,\omega _{c}\right)$ is the cut-off function of the spectral density and there exists different form in the literature. In the original paper^12,49^, a smooth cut-off function of the form $f\left( \omega ,\omega _{c}\right) =\exp \left( -\omega /\omega _{c}\right)$ was introduced and this remains the most used form of the spectral density. An extensive review of the spectral can be found in^48^ where the necessity and justification to introduce a cut-off frequency have been discussed. The coefficient $\omega _{c}^{S-1}$ in equation 92 has the units of frequency to the power of $S-1$, so that $J\left( \omega \right)$ has units of frequency. It is introduced into the functional form to provide an overall coupling strength of the interaction and its value is usually obtained from a fitting process. Instead of using a smooth cut-off function for the spectral density, another possibility is to introduce $f\left( \omega ,\omega _{c}\right) =\left( \sqrt{\omega _{c}^{2}-\omega ^{2}}/\omega _{c}\right) \Theta \left( \omega _{c}-\omega \right)$ where $\Theta \left( \omega _{c}-\omega \right)$ is the step-function^55^.

Among different types of the spectral density, the case when $S=3$ has received the most attention because of its capability to describe three-dimensional acoustic phonon bath. A super-Ohmic bath with $S=3$ was applied in the numerical simulation presented in this work.

### Counter-rotating terms

Additional counter-rotating terms were also included in this work. For example, the counter rotating terms in the Herzberg–Teller interaction give rise to additional off-diagonal elements between the electronic states that capture pure decoherence process which were not studied before. This is especially relevant in the short time scale comparable to the frequency shift caused by the counter-rotating terms.

### Time-dependent decay

The time-dependent decay constants obtained here through the use of cosine and sine functions, before approximating them as Dirac delta function and the Cauchy principal value, are also important results but they are neglected in most of the existing works if one apply the crude assumption that each part of the system coupled to their own environment independently. Even though it has been vastly applied in many studies on molecular vibrational processes^24,25^, the validity of this assumption remains unclear and must not be applied in quantum information processing where the decoherence rates are crucial.

### Herzberg–Teller

The significance of non-Condon effects in molecular spectroscopy has been established both theoretically and experimentally^32,56–58^ but it remains to-date a lack of the description of non-Condon effects from an open quantum system approach. The derivation of non-Condon Liouvillean ($\mathscr {L}_{\mathrm {el-vib-rad} }^{\left( \alpha ,\alpha ^{\prime },i,i^{\prime }\right) }$) given in this work is a first-step towards investigating non-Condon interaction using a master equation approach. Besides the terms that can be expressed in the usual Lindblad form (Eq. 52) as discussed in Section 3, terms that cannot be expressed in Lindblad form with time-dependent decay constants were obtained. The results obtained here are non-trivial even in the case of a simple diatomic molecule. By taking the matrix elements of $\mathscr {L}_{ \mathrm {el-vib-rad}}^{\left( \alpha ,\alpha ^{\prime },i,i^{\prime }\right) }$, we can see that it contributes to the evolution of the diagonal (population) and off-diagonal (coherence) elements of the reduced system density matrix which could easily be missed if one apply the Condon-approximation.

### Cross-term Liouvilleans

An important result of this work are the “cross-term Liouvilleans” that were not considered in any existing works. These terms are usually neglected under the assumption that different part of the system coupled to their own independent environment. However, the validity of this assumption remains unclear and was shown recently that it leads to an incorrect description of the system’s evolution^26,27,59^. By relaxation this assumption, we see that the “cross-term Liouvillean” (e.g. $\mathscr {L}_{\mathrm {(el\times el-vib)-rad}}^{\left( \alpha \right) }$ and $\mathscr {L}_{\mathrm {(el-vib\times el)-rad}}^{\left( \alpha \right) }$) provides additional decoherence to the system (as well as dissipation between the vibronic states) that was not considered previously. For example $\mathscr {L}_{\mathrm {(el\times el-vib)-rad}}^{\left( \alpha \right) \left( +\right) }$ contains terms in the form $\sigma ^{\dagger }\tilde{\rho } _{S}\left( t\right) B_{\alpha i^{\prime }}^{\dagger }=\sigma ^{\dagger } \tilde{\rho }_{S}\left( t\right) b_{i^{\prime }}^{\left( \alpha \right) \dagger }\sigma ^{\dagger }=\left| e\right\rangle \left\langle g\right| \tilde{\rho }_{S}\left( t\right) b_{i^{\prime }}^{\left( \alpha \right) \dagger }\left| e\right\rangle \left\langle g\right|$ that will contributes to the off-diagonal elements between different electronic states, while terms in the form $\sigma \tilde{\rho }_{S}\left( t\right) B_{\alpha i^{\prime }}^{\dagger }=\sigma \tilde{\rho }_{S}\left( t\right) b_{i^{\prime }}^{\left( \alpha \right) \dagger }\sigma ^{\dagger }=\left| g\right\rangle \left\langle e\right| \tilde{\rho }_{S}\left( t\right) b_{i^{\prime }}^{\left( \alpha \right) \dagger }\left| e\right\rangle \left\langle g\right|$ contribute to the diagonal elements. These terms can be significant when molecular quantum memory is considered where accurate decoherence and dissipation rates are essential.

### Molecular Lamb shift

Another important theoretical results of this work are the Lamb-shift terms computed in “Appendix 5”. These terms are usually neglected as it is often assumed that the contribution of Lamb-shift is negligible^60–62^. However, it was shown that these Lamb-shift terms can have significant impact on the dynamics in shorter time-scale^63^ hence it is important to include them in order to achieve a unified theoretical description of the coherent dynamics at any time-scale.

Furthermore, because the assumption that different part of the system coupled to their own independent environment is relaxed in this work, we naturally obtained the Lamb-shift terms that come with the “cross-term Liouvilleans”. These terms have not been considered or discussed in any previous works, an in-depth theoretical analysis of these terms will be given in a future work to investigate the significance of these Lamb-shift terms.

### Spectra of Polyatomic Molecule

Figure 5a shows the spectra of emission lineshape (blue line) versus absorption lineshape (orange line) of nitrogen dioxide for Liouvillean composed of electronic-radiation (non-Condon) and vibrational dampings. As the electronic-phonon interaction is added Fig. 5b, the number of narrow peaks reduces as phonon broadening causes the peaks to become unresolved. Additionally, the effects of Herzberg–Teller interaction and the cross terms depend on the strength of the dipole derivative in $\tilde{\Gamma}_{\left\{ \cdots \right\}}$. The effects of cross terms are very small and noticeable only for small critical frequencies.

Figure 5c.i and c.ii show the effects of varying the cutoff frequencies when the Liouvillean includes all contributions. The spectral peaks are well-resolved for small cutoff frequencies but the broadening causes the spectral lines to coalesce beyond around $5\tilde{v}_{i}$. The absorption(black) and emission(red) spectra for the largest $\omega_c$ are plotted in Fig. 5c.iii versus frequency.

## Conclusions

We have presented a unified theory for impurity molecule (polyatomics) in crystals from an open quantum system approach. The main results of this work are the Herzberg–Teller interaction Liouvillean and the “cross term Liouvillean” which have not been seen in any existing work. Different raising and lowering operators were introduced for the harmonic nuclear potentials in the ground and excited electronic states. This leads to a new form of a Liouvilean containing both electronic and vibrational system operators that is simpler, more insightful and more elegant.

To capture the correct dynamics of the molecular system and include all the possible interactions, several approximations have been relaxed: (a) identical shapes of nuclear potentials; (b) Condon-approximation; (c) rotating wave approximation; (d) the assumption that different parts of the system coupled to their own environment independently. Complete decoherence and dissipation terms due to the radiation and phonon reservoirs obtained in the master equation allow us to correctly model the decay and decoherence rates when using molecules for quantum information processing, particularly for quantum memory.

Another new result of this work is the molecular Lamb-shift appearing in the Liouvilleans, usually neglected as it is often assumed that the contribution of Lamb-shift is negligible^60–62^. It was shown that these Lamb-shift terms can have significant impact on the dynamics in shorter time-scale^63^. Thus, it is important to capture the correct expressions for the molecular system. Furthermore, we have also obtained the Lamb-shift terms in the cross-term Liouvillean.

The validity of the theoretical model is verified as it is reduced to a simplified single vibrational mode and was shown to give the correct (non-trivial) absorption and emission spectra of a diatomic molecule (CO). Thus, the unified theoretical model presented in this work constitutes a proper foundation for our future work where interactions with coherent fields such as laser, magnetic and cavity fields will be included.

## Data Availability

The datasets generated and/or analysed during the current study are not publicly available as it is obtained from digitizing a figure in ref.^45^, but are available from the corresponding author on reasonable request.

## Appendix 1: Intramolecular vibrational redistribution

Intramolecular vibrational redistribution (IVR) is a process in which energy is redistributed between different vibrational normal modes of a vibrationally excited molecule. This will only happen in polyatomic atomic molecule with $N>2$ where *N* is the number of atoms within the molecule of interest. From Eq. (2) we have $\sum _{\mathbf {l}}\frac{1}{2}m_{ \mathbf {l}}\omega _{\mathbf {l}}^{2}\sum _{i,j}x_{i}^{\left( \alpha \right) }x_{j}^{\left( \alpha \right) }$ that accounts for IVR.

For a polyatomic molecule, the potential energy now depends on all the displacement of atoms from their equilibrium positions:

93 $$\begin{aligned} U_{\alpha }^{\left( mol\right) }=U_{\alpha }^{\left( mol\right) }\left( 0\right) +\sum _{i}\left. \frac{\partial U_{\alpha }^{\left( mol\right) }}{ \partial x_{i}^{\left( \alpha \right) }}\right| _{0}x_{i}^{\left( \alpha \right) }+\frac{1}{2}\sum _{i,j}\left. \frac{\partial ^{2}U_{\alpha }^{\left( mol\right) }}{\partial x_{i}^{\left( \alpha \right) }\partial x_{j}^{\left( \alpha \right) }}\right| _{0}x_{i}^{\left( \alpha \right) }x_{j}^{\left( \alpha \right) }+\cdots \end{aligned}$$

where the index $\alpha =\left\{ g,e\right\}$. $U_{\alpha }^{\left( mol\right) }\left( 0\right)$ may be set equal to 0 and the first derivatives also vanishes at equilibrium, thus taking only second order term we have:

94 $$\begin{aligned} U_{\alpha }^{\left( mol\right) }=\frac{1}{2}\sum _{i,j}k_{ij}^{\left( \alpha \right) }x_{i}^{\left( \alpha \right) }x_{j}^{\left( \alpha \right) } \end{aligned}$$

with $k_{ij}^{\left( \alpha \right) }=\left. \frac{\partial ^{2}U_{\alpha }^{\left( mol\right) }}{\partial x_{i}^{\left( \alpha \right) }\partial x_{j}^{\left( \alpha \right) }}\right| _{0}$. So, the IVR is related to the potential $U_{\alpha }^{\left( mol\right) }$ as

95 $$\begin{aligned} V_{\mathrm {vib-vib}}^{\left( \alpha \right) }=\sigma _{\alpha \alpha }U_{\alpha }^{\left( mol\right) }=\frac{1}{2}\sigma _{\alpha \alpha }\left( \sum _{i\ne j}k_{ij}^{\left( \alpha \right) }x_{i}^{\left( \alpha \right) }x_{j}^{\left( \alpha \right) }\right) \end{aligned}$$

## Appendix 2: Linear and quadratic coupling terms

From the definition $x_{i}^{\left( e\right) }=x_{i}^{\left( g\right) }-s_{i}$ and $p_{i}^{\left( e\right) }=p_{i}^{\left( g\right) }$, we may establish the following relationship^64^

96 $$\begin{aligned} b_{i}^{\left( e\right) }&= {} \lambda _{i}^{\left( +\right) }b_{i}^{\left( g\right) }+\lambda _{i}^{\left( -\right) }b_{i}^{\left( g\right) \dagger }-\Lambda _{e}\text {,} \end{aligned}$$

97 $$\begin{aligned} b_{i}^{\left( g\right) }&= {} \lambda _{i}^{\left( +\right) }b_{i}^{\left( e\right) }-\lambda _{i}^{\left( -\right) }b_{i}^{\left( e\right) \dagger }+\Lambda _{g} \end{aligned}$$

with

98 $$\begin{aligned} \lambda _{i}^{\left( \pm \right) }=\frac{1}{2}\left( \sqrt{\frac{\tilde{v} _{i}^{\left( e\right) }}{\tilde{v}_{i}^{\left( g\right) }}}\pm \sqrt{\frac{ \tilde{v}_{i}^{\left( g\right) }}{\tilde{v}_{i}^{\left( e\right) }}}\right) \text {, }\Lambda _{e/g}=s_{i}\sqrt{\frac{m_{i}\tilde{v}_{i}^{\left( e/g\right) }}{2\hbar }}\text {.} \end{aligned}$$

It follows from Eq. (96) that

99 $$\begin{aligned} b_{i}^{\left( e\right) \dagger }b_{i}^{\left( e\right) }=\left( \lambda _{i}^{\left( +\right) 2}+\lambda _{i}^{\left( -\right) 2}\right) b_{i}^{\left( g\right) \dagger }b_{i}^{\left( g\right) }-\Lambda _{e}\left( \lambda _{i}^{\left( -\right) }+\lambda _{i}^{\left( +\right) }\right) \left( b_{i}^{\left( g\right) }+b_{i}^{\left( g\right) \dagger }\right) +\Lambda _{e}^{2}+\lambda _{i}^{\left( -\right) 2} \end{aligned}$$

which we will substitute into Eq. (22):

100 $$\begin{aligned} H_{S}&= {} \hbar \omega _{g}\sigma _{gg}+\hbar \omega _{e}\sigma _{ee}+\sum _{i}\hbar \tilde{v}_{i}^{\left( g\right) }\sigma _{gg}\left( b_{i}^{\left( g\right) \dagger }b_{i}^{\left( g\right) }+\frac{1}{2}\right) +\sum _{i}\hbar \tilde{v}_{i}^{\left( e\right) }\sigma _{ee}\left( b_{i}^{\left( e\right) \dagger }b_{i}^{\left( e\right) }+\frac{1}{2}\right) \nonumber \\&= {} \hbar \omega _{g}\sigma _{gg}+\hbar \omega _{e}\sigma _{ee}+\sum _{i}\hbar \tilde{v}_{i}^{\left( g\right) }\sigma _{gg}\left( b_{i}^{\left( g\right) \dagger }b_{i}^{\left( g\right) }+\frac{1}{2}\right) \nonumber \\&\quad +\, \sum _{i}\hbar \tilde{v}_{i}^{\left( e\right) }\sigma _{ee}\left[ \left( \lambda _{i}^{\left( +\right) 2}+\lambda _{i}^{\left( -\right) 2}\right) b_{i}^{\left( g\right) \dagger }b_{i}^{\left( g\right) }-\Lambda _{e}\left( \lambda _{i}^{\left( -\right) }+\lambda _{i}^{\left( +\right) }\right) \left( b_{i}^{\left( g\right) }+b_{i}^{\left( g\right) \dagger }\right) +\Lambda _{e}^{2}+\lambda _{i}^{\left( -\right) 2}+\frac{1}{2}\right] \text {. } \end{aligned}$$

If we now substitute the definitions for the constants given in Eq. (98), we get

101 $$\begin{aligned} H_{S}&= {} \hbar \omega _{g}\sigma _{gg}+\hbar \omega _{e}\sigma _{ee}+\sum _{i}\hbar \tilde{v}_{i}^{\left( g\right) }\sigma _{gg}\left( b_{i}^{\left( g\right) \dagger }b_{i}^{\left( g\right) }+\frac{1}{2}\right) \nonumber \\&\quad +\, \sum _{i}\sigma _{ee}\left( \frac{1}{2}\hbar \left( \frac{\tilde{v} _{i}^{\left( e\right) 2}-\tilde{v}_{i}^{\left( g\right) 2}}{\tilde{v} _{i}^{\left( g\right) }}\right) \left( b_{i}^{\left( g\right) \dagger }b_{i}^{\left( g\right) }+\frac{1}{2}\right) +\hbar \tilde{v}_{i}^{\left( g\right) }\left( b_{i}^{\left( g\right) \dagger }b_{i}^{\left( g\right) }+ \frac{1}{2}\right) -m_{i}\tilde{v}_{i}^{\left( e\right) 2}s_{i}\sqrt{\frac{ \hbar }{2m_{i}\tilde{v}_{i}^{\left( g\right) }}}\left( b_{i}^{\left( g\right) }+b_{i}^{\left( g\right) \dagger }\right) +s_{i}^{2}\frac{m_{i} \tilde{v}_{i}^{\left( e\right) 2}}{2}\right) \text {.} \end{aligned}$$

One can show that $\left( b_{i}^{\left( g\right) \dagger }b_{i}^{\left( g\right) }+\frac{1}{2}\right) =\frac{1}{2}\left( b_{i}^{\left( g\right) \dagger }+b_{i}^{\left( g\right) }\right) ^{2}$ and this gives us

102 $$\begin{aligned} H_{S}&= {} \hbar \omega _{g}\sigma _{gg}+\hbar \omega _{e}\sigma _{ee}+\sum _{i}\hbar \tilde{v}_{i}^{\left( g\right) }\sigma _{gg}\left( b_{i}^{\left( g\right) \dagger }b_{i}^{\left( g\right) }+\frac{1}{2}\right) \nonumber \\&\quad +\, \sum _{i}\left( \hbar \varkappa _{i}^{\left( 2\right) }\sigma _{ee}\left( b_{i}^{\left( g\right) \dagger }+b_{i}^{\left( g\right) }\right) ^{2}+\hbar \tilde{v}_{i}^{\left( g\right) }\sigma _{ee}\left( b_{i}^{\left( g\right) \dagger }b_{i}^{\left( g\right) }+\frac{1}{2}\right) -\hbar \varkappa _{i}^{\left( 1\right) }\sigma _{ee}\left( b_{i}^{\left( g\right) }+b_{i}^{\left( g\right) \dagger }\right) +s_{i}^{2}\frac{m_{i}\tilde{v} _{i}^{\left( e\right) 2}}{2}\sigma _{ee}\right) \end{aligned}$$

with linear and quadratic coupling constants defined as

103 $$\begin{aligned} \varkappa _{i}^{\left( 1\right) }=\frac{1}{\hbar }m_{i}\tilde{v}_{i}^{\left( e\right) 2}s_{i}\sqrt{\frac{\hbar }{2m_{i}\tilde{v}_{i}^{\left( g\right) }}} \text {, }\varkappa _{i}^{\left( 2\right) }=\left( \frac{\tilde{v} _{i}^{\left( e\right) 2}-\tilde{v}_{i}^{\left( g\right) 2}}{4\tilde{v} _{i}^{\left( g\right) }}\right) \text {.} \end{aligned}$$

Using the fact that $\sigma _{gg}+\sigma _{ee}=I$, we can rearrange the equation to obtain

104 $$\begin{aligned} H_{S}=\hbar \omega _{g}\sigma _{gg}+\hbar \tilde{\omega }_{e}\sigma _{ee}+\sum _{i}\hbar \tilde{v}_{i}^{\left( g\right) }\left( b_{i}^{\left( g\right) \dagger }b_{i}^{\left( g\right) }+\frac{1}{2}\right) +\sum _{i}\left( \hbar \varkappa _{i}^{\left( 2\right) }\sigma _{ee}\left( b_{i}^{\left( g\right) \dagger }+b_{i}^{\left( g\right) }\right) ^{2}-\hbar \varkappa _{i}^{\left( 1\right) }\sigma _{ee}\left( b_{i}^{\left( g\right) }+b_{i}^{\left( g\right) \dagger }\right) \right) \end{aligned}$$

with shifted excited state energy $\hbar \tilde{\omega }_{e}=\left( \hbar \omega _{e}+\sum _{i}s_{i}^{2}\frac{m_{i}\tilde{v}_{i}^{\left( e\right) 2}}{2} \right)$.

## Appendix 3: General derivation of Liouvilleans

In this section, we provide general derivation that would be used to obtain all Liouvilleans including the new Liouvilleans involving different interaction Hamiltonians. Every Hamiltonian can be written in the form

105 $$\begin{aligned} \tilde{V}_{A}=-\sum _{{\alpha }}\hbar \tilde{A}_{{\alpha } }\left( \tilde{R}_{{\alpha }}+\tilde{R}_{{\alpha }}^{\dagger }\right) ,\tilde{V}_{B}=-\sum _{{\alpha }}\hbar \tilde{A}_{\mathbf { \alpha }}\left( \tilde{R}_{{\alpha }}+\tilde{R}_{{\alpha } }^{\dagger }\right) \end{aligned}$$

where $A_{{\alpha }}$ is an arbitrary system operator (or even a combination of system operators) and $R_{{\alpha }}$ some arbitrary bath/reservoir operators for phonon bath ($\alpha =\mathbf {l}$) $R_{{ \alpha }}=d_{\mathbf {l}}$ and for radiation bath ($\alpha =\mathbf {k}$) $R_{ {\alpha }}=a_{\mathbf {k}}$.

The most general form of the Liouvillean is thus

106a $$\begin{aligned} \mathscr {L}_{\mathrm {general}}&= {} -\frac{1}{\hbar ^{2}}\int _{0}^{t}dt^{ \prime }Tr_{\mathrm {R}}\left[ \tilde{V}_{A}\left( t\right) ,\left[ \tilde{V} _{B}\left( t^{\prime }\right) ,\tilde{\rho }_{S}\left( t^{\prime }\right) \otimes \rho _{\mathrm {R}}\right] \right] \end{aligned}$$

106b $$\begin{aligned}&= {} -\frac{1}{\hbar ^{2}}\int _{0}^{t}dt^{\prime }Tr_{\mathrm {R}}\left[ -\sum _{ {\alpha }}\hbar \tilde{A}_{{\alpha }}\left( t\right) \left( \tilde{R}_{{\alpha }}\left( t\right) +\tilde{R}_{{\alpha } }^{\dagger }\left( t\right) \right) ,\left[ -\sum _{{\alpha }^{\prime }}\hbar \tilde{B}_{{\alpha }^{\prime }}\left( t^{\prime }\right) \left( \tilde{R}_{{\alpha }^{\prime }}\left( t^{\prime }\right) + \tilde{R}_{{\alpha }^{\prime }}^{\dagger }\left( t^{\prime }\right) \right) ,\tilde{\rho }_{S}\left( t^{\prime }\right) \otimes \rho _{\mathrm {R}} \right] \right] \end{aligned}$$

106c $$\begin{aligned}&= {} \int _{0}^{t}dt^{\prime }\sum _{{\alpha ,\alpha }^{\prime }}Tr_{ \mathrm {R}}\left[ \tilde{A}_{{\alpha }}\left( t\right) \left( \tilde{R }_{{\alpha }}\left( t\right) +\tilde{R}_{{\alpha }}^{\dagger }\left( t\right) \right) ,\left[ \tilde{B}_{{\alpha }^{\prime }}\left( t^{\prime }\right) \left( \tilde{R}_{{\alpha }^{\prime }}\left( t^{\prime }\right) +\tilde{R}_{{\alpha }^{\prime }}^{\dagger }\left( t^{\prime }\right) \right) ,\tilde{\rho }_{S}\left( t^{\prime }\right) \otimes \rho _{\mathrm {R}}\right] \right] \end{aligned}$$

where *B* is another system operator that may be different from *A*. $Tr_{ \mathrm {R}}$ means tracing over the reservoir/bath and $\rho _{\mathrm {R}}$ is the bath density matrix at thermal equilibrium ($\mathrm {R}=\left\{ \mathrm {ph},\mathrm {rad}\right\}$) If we now expand this we get

107 $$\begin{aligned} \mathscr {L}_{\mathrm {general}}&= {} \int _{0}^{t}dt^{\prime }\sum _{\mathbf { \alpha ,\alpha }^{\prime }}\left\{ Tr_{\mathrm {R}}\left[ \tilde{A}_{\mathbf { \alpha }}\left( t\right) \tilde{R}_{{\alpha }}\left( t\right) ,\left[ \tilde{B}_{{\alpha }^{\prime }}\left( t^{\prime }\right) \tilde{R}_{ {\alpha }^{\prime }}\left( t^{\prime }\right) ,\tilde{\rho }_{S}\left( t^{\prime }\right) \otimes \rho _{\mathrm {R}}\right] \right] +Tr_{\mathrm {R}} \left[ \tilde{A}_{{\alpha }}\left( t\right) \tilde{R}_{{\alpha }}\left( t\right) ,\left[ \tilde{B}_{{\alpha }^{\prime }}\left( t^{\prime }\right) \tilde{R}_{{\alpha }^{\prime }}^{\dagger }\left( t^{\prime }\right) ,\tilde{\rho }_{S}\left( t^{\prime }\right) \otimes \rho _{ \mathrm {R}}\right] \right] \right. \nonumber \\&\left. +Tr_{\mathrm {R}}\left[ \tilde{A}_{{\alpha } }\left( t\right) \tilde{R}_{{\alpha }}^{\dagger }\left( t\right) , \left[ \tilde{B}_{{\alpha }^{\prime }}\left( t^{\prime }\right) \tilde{R}_{{\alpha }^{\prime }}\left( t^{\prime }\right) ,\tilde{\rho } _{S}\left( t^{\prime }\right) \otimes \rho _{\mathrm {R}}\right] \right] +Tr_{ \mathrm {R}}\left[ \tilde{A}_{{\alpha }}\left( t\right) \tilde{R}_{ {\alpha }}^{\dagger }\left( t\right) ,\left[ \tilde{B}_{\mathbf { \alpha }^{\prime }}\left( t^{\prime }\right) \tilde{R}_{{\alpha } ^{\prime }}^{\dagger }\left( t^{\prime }\right) ,\tilde{\rho }_{S}\left( t^{\prime }\right) \otimes \rho _{\mathrm {R}}\right] \right] \right\} \end{aligned}$$

The first and last term vanish because

$$\begin{aligned} Tr_{\mathrm {R}}\left[ \tilde{A}_{{\alpha }}\left( t\right) \tilde{R}_{ {\alpha }}\left( t\right) ,\left[ \tilde{B}_{{\alpha }^{\prime }}\left( t^{\prime }\right) \tilde{R}_{{\alpha }^{\prime }}\left( t^{\prime }\right) ,\tilde{\rho }_{S}\left( t^{\prime }\right) \otimes \rho _{ \mathrm {R}}\right] \right]\propto & {} \left\langle R_{{\alpha }}R_{ {\alpha }^{\prime }}\right\rangle _{\mathrm {R}}=0 \\ Tr_{\mathrm {R}}\left[ \tilde{A}_{{\alpha }}\left( t\right) \tilde{R}_{ {\alpha }}^{\dagger }\left( t\right) ,\left[ \tilde{B}_{\mathbf { \alpha }^{\prime }}\left( t^{\prime }\right) \tilde{R}_{{\alpha } ^{\prime }}^{\dagger }\left( t^{\prime }\right) ,\tilde{\rho }_{S}\left( t^{\prime }\right) \otimes \rho _{\mathrm {R}}\right] \right]\propto & {} \left\langle R_{{\alpha }}^{\dagger }R_{{\alpha }^{\prime }}^{\dagger }\right\rangle _{\mathrm {R}}=0 \end{aligned}$$

This left us with

108 $$\begin{aligned} \mathscr {L}_{\mathrm {general}}&= {} \int _{0}^{t}dt^{\prime }\sum _{\mathbf { \alpha ,\alpha }^{\prime }}\left\{ Tr_{\mathrm {R}}\left[ \tilde{A}_{\mathbf { \alpha }}\left( t\right) \tilde{R}_{{\alpha }}\left( t\right) ,\left[ \tilde{B}_{{\alpha }^{\prime }}\left( t^{\prime }\right) \tilde{R}_{ {\alpha }^{\prime }}^{\dagger }\left( t^{\prime }\right) ,\tilde{\rho } _{S}\left( t^{\prime }\right) \otimes \rho _{\mathrm {R}}\right] \right] +Tr_{ \mathrm {R}}\left[ \tilde{A}_{{\alpha }}\left( t\right) \tilde{R}_{ {\alpha }}^{\dagger }\left( t\right) ,\left[ \tilde{B}_{\mathbf { \alpha }^{\prime }}\left( t^{\prime }\right) \tilde{R}_{{\alpha } ^{\prime }}\left( t^{\prime }\right) ,\tilde{\rho }_{S}\left( t^{\prime }\right) \otimes \rho _{\mathrm {R}}\right] \right] \right\} \nonumber \\&= {} \int _{0}^{t}dt^{\prime }\sum _{{\alpha ,\alpha }^{\prime }}\left\{ Tr_{\mathrm {R}}\left[ \tilde{A}_{{\alpha }}\left( t\right) \tilde{R}_{ {\alpha }}\left( t\right) \tilde{B}_{{\alpha }^{\prime }}\left( t^{\prime }\right) \tilde{R}_{{\alpha }^{\prime }}^{\dagger }\left( t^{\prime }\right) \tilde{\rho }_{S}\left( t^{\prime }\right) \otimes \rho _{\mathrm {R}}\right] -Tr_{\mathrm {R}}\left[ \tilde{B}_{{\alpha } ^{\prime }}\left( t^{\prime }\right) \tilde{R}_{{\alpha }^{\prime }}^{\dagger }\left( t^{\prime }\right) \tilde{\rho }_{S}\left( t^{\prime }\right) \otimes \rho _{\mathrm {R}}\tilde{A}_{{\alpha }}\left( t\right) \tilde{R}_{{\alpha }}\left( t\right) \right] \right. \nonumber \\&\quad -\, Tr_{\mathrm {R}}\left[ \tilde{A}_{{\alpha }}\left( t\right) \tilde{R}_{{\alpha }}\left( t\right) \tilde{\rho }_{S}\left( t^{\prime }\right) \otimes \rho _{\mathrm {R}}\tilde{B}_{{\alpha } ^{\prime }}\left( t^{\prime }\right) \tilde{R}_{{\alpha }^{\prime }}^{\dagger }\left( t^{\prime }\right) \right] +Tr_{\mathrm {R}}\left[ \tilde{ \rho }_{S}\left( t^{\prime }\right) \otimes \rho _{\mathrm {R}}\tilde{B}_{ {\alpha }^{\prime }}\left( t^{\prime }\right) \tilde{R}_{\mathbf { \alpha }^{\prime }}^{\dagger }\left( t^{\prime }\right) \tilde{A}_{\mathbf { \alpha }}\left( t\right) \tilde{R}_{{\alpha }}\left( t\right) \right] \nonumber \\&\quad +\, Tr_{\mathrm {R}}\left[ \tilde{A}_{{\alpha }}\left( t\right) \tilde{R}_{{\alpha }}^{\dagger }\left( t\right) \tilde{B}_{ {\alpha }^{\prime }}\left( t^{\prime }\right) \tilde{R}_{\mathbf { \alpha }^{\prime }}\left( t^{\prime }\right) \tilde{\rho }_{S}\left( t^{\prime }\right) \otimes \rho _{\mathrm {R}}\right] -Tr_{\mathrm {R}}\left[ \tilde{B}_{{\alpha }^{\prime }}\left( t^{\prime }\right) \tilde{R}_{ {\alpha }^{\prime }}\left( t^{\prime }\right) \tilde{\rho }_{S}\left( t^{\prime }\right) \otimes \rho _{\mathrm {R}}\tilde{A}_{{\alpha } }\left( t\right) \tilde{R}_{{\alpha }}^{\dagger }\left( t\right) \right] \nonumber \\&\left. \quad -\, Tr_{\mathrm {R}}\left[ \tilde{A}_{{\alpha } }\left( t\right) \tilde{R}_{{\alpha }}^{\dagger }\left( t\right) \tilde{\rho }_{S}\left( t^{\prime }\right) \otimes \rho _{\mathrm {R}}\tilde{B} _{{\alpha }^{\prime }}\left( t^{\prime }\right) \tilde{R}_{\mathbf { \alpha }^{\prime }}\left( t^{\prime }\right) \right] +Tr_{\mathrm {R}}\left[ \tilde{\rho }_{S}\left( t^{\prime }\right) \otimes \rho _{\mathrm {R}}\tilde{B} _{{\alpha }^{\prime }}\left( t^{\prime }\right) \tilde{R}_{\mathbf { \alpha }^{\prime }}\left( t^{\prime }\right) \tilde{A}_{{\alpha } }\left( t\right) \tilde{R}_{{\alpha }}^{\dagger }\left( t\right) \right] \right\} \end{aligned}$$

Tracing out the system operators

109 $$\begin{aligned} \mathscr {L}_{\mathrm {general}}&= {} \int _{0}^{t}dt^{\prime }\sum _{\mathbf { \alpha ,\alpha }^{\prime }}\left\{ \tilde{A}_{{\alpha }}\left( t\right) \tilde{B}_{{\alpha }^{\prime }}\left( t^{\prime }\right) \tilde{\rho }_{S}\left( t^{\prime }\right) Tr_{\mathrm {R}}\left[ \tilde{R}_{ {\alpha }}\left( t\right) \tilde{R}_{{\alpha }^{\prime }}^{\dagger }\left( t^{\prime }\right) \rho _{\mathrm {R}}\right] -\tilde{B}_{ {\alpha }^{\prime }}\left( t^{\prime }\right) \tilde{\rho }_{S}\left( t^{\prime }\right) \tilde{A}_{{\alpha }}\left( t\right) Tr_{\mathrm {R} }\left[ \tilde{R}_{{\alpha }}\left( t\right) \tilde{R}_{\mathbf { \alpha }^{\prime }}^{\dagger }\left( t^{\prime }\right) \rho _{\mathrm {R}} \right] \right. \nonumber \\&\quad -\, \tilde{A}_{{\alpha }}\left( t\right) \tilde{\rho } _{S}\left( t^{\prime }\right) \tilde{B}_{{\alpha }^{\prime }}\left( t^{\prime }\right) Tr_{\mathrm {R}}\left[ \tilde{R}_{{\alpha }^{\prime }}^{\dagger }\left( t^{\prime }\right) \tilde{R}_{{\alpha }}\left( t\right) \rho _{\mathrm {R}}\right] +\tilde{\rho }_{S}\left( t^{\prime }\right) \tilde{B}_{{\alpha }^{\prime }}\left( t^{\prime }\right) \tilde{A}_{{\alpha }}\left( t\right) Tr_{\mathrm {R}}\left[ \tilde{R}_{ {\alpha }^{\prime }}^{\dagger }\left( t^{\prime }\right) \tilde{R}_{ {\alpha }}\left( t\right) \rho _{\mathrm {R}}\right] \nonumber \\&\quad +\, \tilde{A}_{{\alpha }}\left( t\right) \tilde{B}_{ {\alpha }^{\prime }}\left( t^{\prime }\right) \tilde{\rho }_{S}\left( t^{\prime }\right) Tr_{\mathrm {R}}\left[ \tilde{R}_{{\alpha } }^{\dagger }\left( t\right) \tilde{R}_{{\alpha }^{\prime }}\left( t^{\prime }\right) \rho _{\mathrm {R}}\right] -\tilde{B}_{{\alpha } ^{\prime }}\left( t^{\prime }\right) \tilde{\rho }_{S}\left( t^{\prime }\right) \tilde{A}_{{\alpha }}\left( t\right) Tr_{\mathrm {R}}\left[ \tilde{R}_{{\alpha }}^{\dagger }\left( t\right) \tilde{R}_{\mathbf { \alpha }^{\prime }}\left( t^{\prime }\right) \rho _{\mathrm {R}}\right] \nonumber \\&\left. \quad -\, \tilde{A}_{{\alpha }}\left( t\right) \tilde{ \rho }_{S}\left( t^{\prime }\right) \tilde{B}_{{\alpha }^{\prime }}\left( t^{\prime }\right) Tr_{\mathrm {R}}\left[ \tilde{R}_{{\alpha } ^{\prime }}\left( t^{\prime }\right) \tilde{R}_{{\alpha }}^{\dagger }\left( t\right) \rho _{\mathrm {R}}\right] +\tilde{\rho }_{S}\left( t^{\prime }\right) \tilde{B}_{{\alpha }^{\prime }}\left( t^{\prime }\right) \tilde{A}_{{\alpha }}\left( t\right) Tr_{\mathrm {R}}\left[ \tilde{R}_{ {\alpha }^{\prime }}\left( t^{\prime }\right) \tilde{R}_{\mathbf { \alpha }}^{\dagger }\left( t\right) \rho _{\mathrm {R}}\right] \right\} \end{aligned}$$

The bath correlation functions give

110 $$\begin{aligned} Tr_{\mathrm {R}}\left[ \tilde{R}_{{\alpha }}\left( t\right) \tilde{R}_{ {\alpha }^{\prime }}^{\dagger }\left( t^{\prime }\right) \rho _{ \mathrm {R}}\right]&= {} \delta _{\alpha ,\alpha ^{\prime }}\left[ \bar{n} \left( \omega _{\alpha }\right) +1\right] e^{-i\omega _{\alpha }\left( t-t^{\prime }\right) } \end{aligned}$$

111 $$\begin{aligned} Tr_{\mathrm {R}}\left[ \tilde{R}_{{\alpha }^{\prime }}^{\dagger }\left( t^{\prime }\right) \tilde{R}_{{\alpha }}\left( t\right) \rho _{\mathrm {R}}\right]&= {} \delta _{\alpha ,\alpha ^{\prime }}\bar{n}\left( \omega _{\alpha }\right) e^{-i\omega _{\alpha }\left( t-t^{\prime }\right) } \end{aligned}$$

Grouping up the terms with common factors

112 $$\begin{aligned} \mathscr {L}_{\mathrm {general}}&= {} \int _{0}^{t}dt^{\prime }\sum _{\mathbf { \alpha }}\left\{ \left( \begin{array}{c} \tilde{A}_{{\alpha }}\left( t\right) \tilde{B}_{{\alpha } }\left( t^{\prime }\right) \tilde{\rho }_{S}\left( t^{\prime }\right) e^{-i\omega _{\alpha }\left( t-t^{\prime }\right) }-\tilde{B}_{\mathbf { \alpha }}\left( t^{\prime }\right) \tilde{\rho }_{S}\left( t^{\prime }\right) \tilde{A}_{{\alpha }}\left( t\right) e^{-i\omega _{\alpha }\left( t-t^{\prime }\right) } \\ -\tilde{A}_{{\alpha }}\left( t\right) \tilde{\rho }_{S}\left( t^{\prime }\right) \tilde{B}_{{\alpha }}\left( t^{\prime }\right) e^{i\omega _{\alpha }\left( t-t^{\prime }\right) }+\tilde{\rho }_{S}\left( t^{\prime }\right) \tilde{B}_{{\alpha }}\left( t^{\prime }\right) \tilde{A}_{{\alpha }}\left( t\right) e^{i\omega _{\alpha }\left( t-t^{\prime }\right) } \end{array} \right) \left[ \bar{n}\left( \omega _{\alpha }\right) +1\right] \right. \nonumber \\&\left. \quad +\, \left( \begin{array}{c} \tilde{A}_{{\alpha }}\left( t\right) \tilde{B}_{{\alpha } }\left( t^{\prime }\right) \tilde{\rho }_{S}\left( t^{\prime }\right) e^{i\omega _{\alpha }\left( t-t^{\prime }\right) }-\tilde{B}_{{\alpha }}\left( t^{\prime }\right) \tilde{\rho }_{S}\left( t^{\prime }\right) \tilde{ A}_{{\alpha }}\left( t\right) e^{i\omega _{\alpha }\left( t-t^{\prime }\right) } \\ -\tilde{A}_{{\alpha }}\left( t\right) \tilde{\rho }_{S}\left( t^{\prime }\right) \tilde{B}_{{\alpha }}\left( t^{\prime }\right) e^{-i\omega _{\alpha }\left( t-t^{\prime }\right) }+\tilde{\rho }_{S}\left( t^{\prime }\right) \tilde{B}_{{\alpha }}\left( t^{\prime }\right) \tilde{A}_{{\alpha }}\left( t\right) e^{-i\omega _{\alpha }\left( t-t^{\prime }\right) } \end{array} \right) \bar{n}\left( \omega _{\alpha }\right) \right\} \end{aligned}$$

If we invoke the Markov approximation, we can extend the integration limit to $\infty$ and replace $\tilde{\rho }_{S}\left( t^{\prime }\right) \longrightarrow \tilde{\rho }_{S}\left( t\right)$

113 $$\begin{aligned} \mathscr {L}_{\mathrm {general}}&= {} \int _{0}^{\infty }dt^{\prime }\sum _{ {\alpha }}\left\{ \left( \begin{array}{c} \tilde{A}_{{\alpha }}\left( t\right) \tilde{B}_{{\alpha } }\left( t^{\prime }\right) \tilde{\rho }_{S}\left( t\right) e^{-i\omega _{\alpha }\left( t-t^{\prime }\right) }-\tilde{B}_{{\alpha }}\left( t^{\prime }\right) \tilde{\rho }_{S}\left( t\right) \tilde{A}_{{\alpha }}\left( t\right) e^{-i\omega _{\alpha }\left( t-t^{\prime }\right) } \\ -\tilde{A}_{{\alpha }}\left( t\right) \tilde{\rho }_{S}\left( t\right) \tilde{B}_{{\alpha }}\left( t^{\prime }\right) e^{i\omega _{\alpha }\left( t-t^{\prime }\right) }+\tilde{\rho }_{S}\left( t\right) \tilde{B}_{ {\alpha }}\left( t^{\prime }\right) \tilde{A}_{{\alpha } }\left( t\right) e^{i\omega _{\alpha }\left( t-t^{\prime }\right) } \end{array} \right) \left[ \bar{n}\left( \omega _{\alpha }\right) +1\right] \right. \nonumber \\&\left. \quad +\, \left( \begin{array}{c} \tilde{A}_{{\alpha }}\left( t\right) \tilde{B}_{{\alpha } }\left( t^{\prime }\right) \tilde{\rho }_{S}\left( t\right) e^{i\omega _{\alpha }\left( t-t^{\prime }\right) }-\tilde{B}_{{\alpha }}\left( t^{\prime }\right) \tilde{\rho }_{S}\left( t\right) \tilde{A}_{{\alpha }}\left( t\right) e^{i\omega _{\alpha }\left( t-t^{\prime }\right) } \\ -\tilde{A}_{{\alpha }}\left( t\right) \tilde{\rho }_{S}\left( t\right) \tilde{B}_{{\alpha }}\left( t^{\prime }\right) e^{-i\omega _{\alpha }\left( t-t^{\prime }\right) }+\tilde{\rho }_{S}\left( t\right) \tilde{B}_{ {\alpha }}\left( t^{\prime }\right) \tilde{A}_{{\alpha } }\left( t\right) e^{-i\omega _{\alpha }\left( t-t^{\prime }\right) } \end{array} \right) \bar{n}\left( \omega _{\alpha }\right) \right\} \end{aligned}$$

$A_{{\alpha }}$ and $B_{{\alpha }}$ are Hermitian ($A_{ {\alpha }}=A_{{\alpha }}^{\dagger }$, $B_{{\alpha }}=B_{ {\alpha }}^{\dagger }$). We can just write half of the terms

114 $$\begin{aligned} \mathscr {L}_{\mathrm {general}}&= {} \int _{0}^{\infty }dt^{\prime }\sum _{ {\alpha }}\left\{ \left[ \tilde{\rho }_{S}\left( t\right) \tilde{B}_{ {\alpha }}\left( t^{\prime }\right) \tilde{A}_{{\alpha } }\left( t\right) -\tilde{A}_{{\alpha }}\left( t\right) \tilde{\rho } _{S}\left( t\right) \tilde{B}_{{\alpha }}\left( t^{\prime }\right) \right] e^{i\omega _{\alpha }\left( t-t^{\prime }\right) }\left[ \bar{n}\left( \omega _{\alpha }\right) +1\right] \right. \nonumber \\&\left. \quad +\, \left[ \tilde{\rho }_{S}\left( t\right) \tilde{B}_{ {\alpha }}\left( t^{\prime }\right) \tilde{A}_{{\alpha } }\left( t\right) -\tilde{A}_{{\alpha }}\left( t\right) \tilde{\rho } _{S}\left( t\right) \tilde{B}_{{\alpha }}\left( t^{\prime }\right) \right] e^{-i\omega _{\alpha }\left( t-t^{\prime }\right) }\bar{n}\left( \omega _{\alpha }\right) +H.c.\right\} \end{aligned}$$

115 $$\begin{aligned}&= {} -\int _{0}^{\infty }dt^{\prime }\sum _{\mathbf {\beta }}\left\{ O_{\beta }\left( A_{\mathbf {\beta }},t;B_{\mathbf {\beta }},t^{\prime }\right) \left[ e^{i\omega _{\mathbf {\beta }}\left( t-t^{\prime }\right) }\left[ n\left( \omega _{\mathbf {\beta }}\right) +1\right] +e^{-i\omega _{\mathbf {\beta } }\left( t-t^{\prime }\right) }n\left( \omega _{\mathbf {\beta }}\right) \right] +H.c.\right\} \end{aligned}$$

where one only needs to calculate

116 $$\begin{aligned} O_{\beta }\left( A,t;B,t^{\prime }\right) =\left[ \tilde{\rho }_{S}\left( t\right) B_{\mathbf {\beta }}\left( t^{\prime }\right) A_{\mathbf {\beta } }\left( t\right) -A_{\mathbf {\beta }}\left( t\right) \tilde{\rho }_{S}\left( t\right) B_{\mathbf {\beta }}\left( t^{\prime }\right) \right] \end{aligned}$$

## Appendix 4: Derivation of decay terms and spectral density

We invoke the Markov approximation now by extending the integration limit to $\infty$ and replace $\tilde{\rho }_{S}\left( t-\tau \right) \longrightarrow \tilde{\rho }_{S}\left( t\right)$

We change the summation $\sum _{\mathbf {k}}$ to an integration by introducing a density of states $\mathscr {D}\left( \omega \right)$ such that

117 $$\begin{aligned} \sum _{\mathbf {k}}\cdots \longrightarrow \int d^{3}k~\mathscr {D}\left( \mathbf {k} \right) \cdots \rightarrow \int d^{3}k\rightarrow \int _{0}^{\infty }d\omega _{k}\int _{0}^{\pi }\sin \theta ~d\theta \int _{0}^{2\pi }d\phi \frac{\omega _{k}^{2}V}{8\pi ^{3}c^{3}} \end{aligned}$$

For isotropic free space the integration is evaluated by changing to spherical coordinates in $\mathbf {k}$-space $\mathscr {D}\left( \mathbf {k} \right) d^{3}k\longrightarrow \frac{\omega ^{2}V}{8\pi ^{3}c^{3}}d\omega \sin \theta ~d\theta ~d\phi$ where we use the density of states $\mathscr {D} \left( \mathbf {k}\right)$ of the radiation bath given in^34^. Inserting the expression for $\varsigma _{i,\mathbf {k}}^{\left( ge\right) }$ and $\varsigma _{i,\mathbf {k}}^{\left( ge\right) *}$

118 $$\begin{aligned} \text { }\Gamma _{\left\{ \cdots \right\} }\left( \omega _{eg}\right)&= {} \sum _{i,i^{\prime }}\int d^{3}k~D\left( \mathbf {k}\right) \varsigma _{i, \mathbf {k}}^{\left( ge\right) }\varsigma _{i^{\prime },\mathbf {k}}^{\left( ge\right) }\pi \delta \left( \omega _{k}-\omega _{eg}\right) \left\{ \cdots \right\} \nonumber \\&= {} \sum _{i,i^{\prime }}\sum _{\lambda }\int _{0}^{\infty }d\omega _{k}\int _{0}^{\pi }\sin \theta ~d\theta \int _{0}^{2\pi }d\phi \frac{\omega _{k}^{2}V}{8\pi ^{3}c^{3}}\left( \frac{1}{\hbar }\mathbf {M}_{i}^{\left( eg\right) 0}\cdot E _{\mathbf {k}}\right) \left( \frac{1}{\hbar } \mathbf {M}_{i^{\prime }}^{\left( eg\right) 0}\cdot E _{\mathbf {k} }\right) \pi \delta \left( \omega _{k}-\omega _{eg}\right) \left\{ \cdots \right\} \nonumber \\&= {} \sum _{i,i^{\prime }}\sum _{\lambda }\int _{0}^{\infty }d\omega _{k}\int _{0}^{\pi }\sin \theta ~d\theta \int _{0}^{2\pi }d\phi \frac{\omega _{k}^{2}V}{8\pi ^{3}c^{3}}\frac{\hbar \omega _{k}}{2\varepsilon _{0}V}\left( \frac{1}{\hbar }\mathbf {M}_{i}^{\left( eg\right) 0}\cdot {\varepsilon }_{ \mathbf {k}}\right) \left( \frac{1}{\hbar }\mathbf {M}_{i^{\prime }}^{\left( eg\right) 0}\cdot {\varepsilon }_{\mathbf {k}}\right) \pi \delta \left( \omega _{k}-\omega _{eg}\right) \left\{ \cdots \right\} \end{aligned}$$

The terms $\exp \left[ \pm i\mathbf {k}\cdot \mathbf {r}\right]$ cancel. If we evaluate the Dirac delta function, we get

119 $$\begin{aligned} \Gamma _{\left\{ \cdots \right\} }\left( \omega _{eg}\right) =\sum _{i,i^{\prime }}\sum _{\lambda }\int _{0}^{\pi }\sin \theta ~d\theta \int _{0}^{2\pi }d\phi \frac{1}{2}\frac{\omega _{eg}^{3}}{8\varepsilon _{0}\pi ^{2}\hbar c^{3}}\left( \mathbf {M}_{i}^{\left( eg\right) 0}\cdot {\varepsilon }_{\mathbf {k}}\right) \left( \mathbf {M}_{i^{\prime }}^{\left( eg\right) 0}\cdot {\varepsilon }_{\mathbf {k}}\right) \left\{ \cdots \right\} \end{aligned}$$

To evaluate the term $\left( \mathbf {M}_{i}^{\left( eg\right) 0}\cdot {\varepsilon }_{\mathbf {k}}\right) ^{2}$, we make the same assumption as in^34^ and^47^ such that for each $\mathbf {k}$ we choose the orthogonal polarization state $\lambda _{1}$ and $\lambda _{2}$, such that the first polarization state $\lambda _{1}$ gives $\mathbf {M }_{i}^{\left( eg\right) 0}\cdot {\varepsilon }_{\mathbf {k}}=0$. Then, for the second polarization state $\lambda _{2}$, we find

120 $$\begin{aligned} \left( \mathbf {M}_{i}^{\left( eg\right) 0}\cdot {{\varepsilon }}_{\mathbf {k }}\right) \left( \mathbf {M}_{i^{\prime }}^{\left( eg\right) 0}\cdot {{\varepsilon }}_{\mathbf {k}}\right) =\left| M_{i}^{\left( eg\right) 0}\right| \left| M_{i^{\prime }}^{\left( eg\right) 0}\right| \left( 1-\cos ^{2}\theta \right) \end{aligned}$$

For each $\mathbf {k}$ we have two orthogonal polarization directions, $\lambda _{1}$ and $\lambda _{2}$. The free space spontaneous emission rate $\Gamma =\frac{\tilde{\omega }_{eg}^{3}\left| {\mu }_{ge}^{\left( 0\right) }\right| ^{2}}{3\pi \varepsilon _{0}\hbar c^{3}}$ and we have used $\int _{0}^{\pi }\sin \theta ~d\theta \int _{0}^{2\pi }d\phi \left( 1-\cos ^{2}\theta \right) =\frac{8\pi }{3}$ hence we have expressions for the decay constants $\Gamma _{\left\{ \cdots \right\} }$, $\tilde{\Gamma } _{\left\{ \cdots \right\} }^{\left( \alpha ,\alpha ^{\prime },i,i^{\prime }\right) }$, $\hat{\Gamma }_{\left\{ \cdots \right\} }^{\left( \alpha ,i,i^{\prime }\right) }$ and $\check{\Gamma }_{\left\{ \cdots \right\} }^{\left( \alpha ,i,i^{\prime }\right) }$, where each one follows the same procedure by simply replacing the coupling constants;

121a $$\begin{aligned} \Gamma _{\left\{ \cdots \right\} }\left( \omega _{eg}\right)&= {} \sum _{i,i^{\prime }}\frac{1}{2}\frac{1}{4\varepsilon _{0}\pi }\frac{ 4\omega _{eg}^{3}\left| \mathbf {M}_{i}^{\left( eg\right) 0}\right| \left| \mathbf {M}_{i^{\prime }}^{\left( eg\right) 0}\right| }{3\hbar c^{3}}\left\{ \cdots \right\} \end{aligned}$$

121b $$\begin{aligned} \tilde{\Gamma }_{\left\{ \cdots \right\} }^{\left( *\right) }&= {} \tilde{ \Gamma }_{\left\{ \cdots \right\} }^{\left( \alpha ,\alpha ^{\prime },i,i^{\prime }\right) }=\frac{1}{2}\frac{1}{4\varepsilon _{0}\pi }\frac{ 4v^{3}\left| \mathbf {M}_{i}^{\left( eg\right) \alpha }\right| \left| \mathbf {M}_{i^{\prime }}^{\left( eg\right) \alpha ^{\prime }}\right| }{3\hbar c^{3}}\left\{ \cdots \right\} \end{aligned}$$

121c $$\begin{aligned} \hat{\Gamma }_{\left\{ \cdots \right\} }^{\left( \alpha ,i,i^{\prime }\right) }&= {} \frac{1}{2}\frac{1}{4\varepsilon _{0}\pi }\frac{4v^{3}\left| \mathbf {M}_{i}^{\left( eg\right) 0}\right| \left| \mathbf {M} _{i^{\prime }}^{\left( eg\right) \alpha }\right| }{3\hbar c^{3}}\left\{ \cdots \right\} \end{aligned}$$

121d $$\begin{aligned} \check{\Gamma }_{\left\{ \cdots \right\} }^{\left( \alpha ,i,i^{\prime }\right) }&= {} \frac{1}{2}\frac{1}{4\varepsilon _{0}\pi }\frac{ 4v^{3}\left| \mathbf {M}_{i}^{\left( eg\right) \alpha }\right| \left| \mathbf {M}_{i^{\prime }}^{\left( eg\right) 0}\right| }{3\hbar c^{3}}\left\{ \cdots \right\} \end{aligned}$$

Note that the summation over *i* and $i^{\prime }$ is not included in the definitions of $\tilde{\Gamma }_{\left\{ \cdots \right\} }^{\left( \alpha ,\alpha ^{\prime },i,i^{\prime }\right) }$, $\hat{\Gamma }_{\left\{ \cdots \right\} }^{\left( \alpha ,i,i^{\prime }\right) }$ and $\check{\Gamma } _{\left\{ \cdots \right\} }^{\left( \alpha ,i,i^{\prime }\right) }$ because the system operators associated with their respective Liouvillean have *i* and $i^{\prime }$ dependence unlike that of $\Gamma _{\left\{ \cdots \right\} }$.

For interactions with phonon bath, we replace the summation over phonon density of states

122 $$\begin{aligned} \int d^{3}l~\digamma \left( \mathbf {l}\right) \Theta _{i\mathbf {l}}^{\left( \alpha \right) }\Theta _{i^{\prime }\mathbf {l}}^{\left( \alpha ^{\prime }\right) }\cdots \longrightarrow \int d\omega ~J\left( \omega \right) \ldots \end{aligned}$$

where we assumed the same spectral density $J\left( \omega \right)$ applies for different electronic state $\alpha$ and vibrational mode *i*. This gives us

123 $$\begin{aligned} \Upsilon _{\left\{ \cdots \right\} }^{\left( *\right) }\left( \tilde{v} _{i^{\prime }}^{\left( \alpha ^{\prime }\right) }\right) =\int d\omega ~J\left( \omega \right) \pi \delta \left( \omega _{l}-\tilde{v}_{i^{\prime }}^{\left( \alpha ^{\prime }\right) }\right) \left\{ \cdots \right\} =\pi J\left( \tilde{v}_{i^{\prime }}^{\left( \alpha ^{\prime }\right) }\right) \left\{ \cdots \right\} _{\tilde{v}_{i^{\prime }}^{\left( \alpha ^{\prime }\right) }} \end{aligned}$$

In the case where the vibrational motion of the molecule is coupled to a three-dimensional acoustic phonon bath, the spectral density $J\left( \omega \right)$ takes the functional form of a super-Ohmic bath^12^

124 $$\begin{aligned} J\left( \omega \right) =\lambda \frac{\omega ^{3}}{\omega _{c}^{2}}\exp \left( -\omega /\omega _{c}\right) \end{aligned}$$

with the phonon cut-off frequency $\omega _{c}$ (typically the same order of magnitude as vibrational modes frequency) and a parameter $\lambda$ for the overall coupling strength of the interaction. The value of $\lambda$ is usually determined by the fitting process. This functional form has been applied for DBT molecule in thin nano-crystals of anthracene^11^ , electron-phonon coupling of color center in diamond^54^ and InAs/GaAs quantum dots.

## Appendix 5: Lamb shift terms

In this section we give the full expressions of the Lamb shift terms that come with each Liouvilleans. For $\mathscr {L}_{\mathrm {el-vib-ph}}^{\left( \alpha ,\alpha ^{\prime }\right) }$, we have the Lamb shift

125 $$\begin{aligned} \Lambda _{\mathrm {el-vib-ph}}^{\left( *\right) }&= {} i\left\{ \Delta Y_{\left( +,-\right) }^{\left( *\right) }\left[ e^{i\Delta \tilde{v} _{i,i^{\prime }}^{\left( +\right) \left( \alpha ,\alpha ^{\prime }\right) }t}\left( S_{\alpha ,i}^{\dagger }\tilde{\rho }_{S}\left( t\right) S_{\alpha ^{\prime },i^{\prime }}^{\dagger }-\tilde{\rho }_{S}\left( t\right) S_{\alpha ^{\prime },i^{\prime }}^{\dagger }S_{\alpha ,i}^{\dagger }\right) \right. \right. \nonumber \\&\left. \quad +\, e^{-i\Delta \tilde{v}_{i,i^{\prime }}^{\left( -\right) \left( \alpha ,\alpha ^{\prime }\right) }t}\left( S_{\alpha ,i} \tilde{\rho }_{S}\left( t\right) S_{\alpha ^{\prime },i^{\prime }}^{\dagger }- \tilde{\rho }_{S}\left( t\right) S_{\alpha ^{\prime },i^{\prime }}^{\dagger }S_{\alpha ,i}\right) \right] \nonumber \\&\quad +\, \Delta Y_{\left( -,+\right) }^{\left( *\right) }\left[ e^{i\Delta \tilde{v}_{i,i^{\prime }}^{\left( -\right) \left( \alpha ,\alpha ^{\prime }\right) }t}\left( S_{\alpha ,i}^{\dagger }\tilde{\rho }_{S}\left( t\right) S_{\alpha ^{\prime },i^{\prime }}-\tilde{\rho }_{S}\left( t\right) S_{\alpha ^{\prime },i^{\prime }}S_{\alpha ,i}^{\dagger }\right) \right. \nonumber \\&\left. \left. \quad +\, e^{-i\Delta \tilde{v}_{i,i^{\prime }}^{\left( +\right) \left( \alpha ,\alpha ^{\prime }\right) }t}\left( S_{\alpha ,i}\tilde{\rho }_{S}\left( t\right) S_{\alpha ^{\prime },i^{\prime }}-\tilde{\rho }_{S}\left( t\right) S_{\alpha ^{\prime },i^{\prime }}S_{\alpha ,i}\right) \right] \right\} +H.c. \end{aligned}$$

where we have defined

126 $$\begin{aligned} \Delta Y_{\left( \pm ,\mp \right) }^{\left( *\right) }&= {} Y_{\left\{ n+1\right\} \pm }^{\left( *\right) }-Y_{\left\{ n\right\} \mp }^{\left( *\right) } \end{aligned}$$

127 $$\begin{aligned} Y_{\left\{ \cdots \right\} \pm }^{\left( *\right) }&= {} Y_{\left\{ \cdots \right\} }^{\left( \alpha ,\alpha ^{\prime },i,i^{\prime }\right) }\left( \pm \tilde{v}_{i^{\prime }}^{\left( \alpha ^{\prime }\right) }\right) =\int d^{3}l~\digamma \left( \mathbf {l}\right) \Theta _{i\mathbf {l}}^{\left( \alpha \right) }\Theta _{i^{\prime }\mathbf {l}}^{\left( \alpha ^{\prime }\right) }\int _{0}^{t}d\tau \sin \left( \omega _{l}\mp \tilde{v}_{i^{\prime }}^{\left( \alpha ^{\prime }\right) }\right) \tau \left\{ \cdots \right\} \nonumber \\&= {} \int d^{3}l~\digamma \left( \mathbf {l}\right) \Theta _{i\mathbf {l} }^{\left( \alpha \right) }\Theta _{i^{\prime }\mathbf {l}}^{\left( \alpha ^{\prime }\right) }\frac{P}{\left( \omega _{l}\mp \tilde{v}_{i^{\prime }}^{\left( \alpha ^{\prime }\right) }\right) }\left\{ \cdots \right\} \end{aligned}$$

with $$\int _{0}^{t}d\tau e^{\pm ix\tau }\cdots =\int _{0}^{t}d\tau (\cos x\tau \pm i\sin x\tau )\ldots $$

Next the Lamb shift in $\mathscr {L}_{\mathrm {el-rad}}$ is

128 $$\begin{aligned} \Lambda _{\mathrm {el-rad}}&= {} -i\sum _{i,i^{\prime }}\left[ \begin{array}{c} \Im _{\left\{ n+1\right\} }^{\left( i,i^{\prime }\right) +}\left( \tilde{\rho }_{S}\left( t\right) \sigma ^{\dagger }\sigma -\sigma \tilde{\rho }_{S}\left( t\right) \sigma ^{\dagger }-e^{i2\omega _{eg}t}\sigma ^{\dagger }\tilde{\rho } _{S}\left( t\right) \sigma ^{\dagger }\right) \\ -\Im _{\left\{ n\right\} }^{\left( i,i^{\prime }\right) -}\left( \tilde{\rho } _{S}\left( t\right) \sigma ^{\dagger }\sigma -\sigma \tilde{\rho }_{S}\left( t\right) \sigma ^{\dagger }-e^{i2\omega _{eg}t}\sigma ^{\dagger }\tilde{\rho } _{S}\left( t\right) \sigma ^{\dagger }\right) \\ -\Im _{\left\{ n\right\} }^{\left( i,i^{\prime }\right) +}\left( \tilde{\rho } _{S}\left( t\right) \sigma \sigma ^{\dagger }-\sigma ^{\dagger }\tilde{\rho } _{S}\left( t\right) \sigma -e^{-i2\omega _{eg}t}\sigma \tilde{\rho } _{S}\left( t\right) \sigma \right) \\ +\Im _{\left\{ n+1\right\} }^{\left( i,i^{\prime }\right) -}\left( \tilde{ \rho }_{S}\left( t\right) \sigma \sigma ^{\dagger }-\sigma ^{\dagger }\tilde{ \rho }_{S}\left( t\right) \sigma -e^{-i2\omega _{eg}t}\sigma \tilde{\rho } _{S}\left( t\right) \sigma \right) \end{array} \right] +H.c. \end{aligned}$$

129 $$\begin{aligned}&= {} i\sum _{i,i^{\prime }}\left\{ \Delta \Im _{\left( +,-\right) }^{\left( i,i^{\prime }\right) }\left( \sigma \tilde{\rho }_{S}\left( t\right) \sigma ^{\dagger }-\tilde{\rho }_{S}\left( t\right) \sigma ^{\dagger }\sigma +e^{i2\omega _{eg}t}\sigma ^{\dagger }\tilde{\rho }_{S}\left( t\right) \sigma ^{\dagger }\right) \right. \nonumber \\&\left. \quad +\, \Delta \Im _{\left( -,+\right) }^{\left( i,i^{\prime }\right) }\left( \sigma ^{\dagger }\tilde{\rho }_{S}\left( t\right) \sigma -\tilde{\rho }_{S}\left( t\right) \sigma \sigma ^{\dagger }+e^{-i2\omega _{eg}t}\sigma \tilde{\rho }_{S}\left( t\right) \sigma \right) \right\} +H.c. \end{aligned}$$

with

130 $$\begin{aligned} \Delta \Im _{\left( \pm ,\mp \right) }^{\left( i,i^{\prime }\right) }&= {} \Im _{\left\{ n+1\right\} \pm }^{\left( i,i^{\prime }\right) }-\Im _{\left\{ n\right\} \mp }^{\left( i,i^{\prime }\right) } \end{aligned}$$

131 $$\begin{aligned} \Im _{\left\{ \cdots \right\} \pm }^{\left( i,i^{\prime }\right) }\left( \pm \omega _{eg}\right)&= {} \int d^{3}k~D\left( \mathbf {k}\right) \varsigma _{i, \mathbf {k}}^{\left( ge\right) }\varsigma _{i^{\prime },\mathbf {k}}^{\left( ge\right) }P\left( \omega _{k}\mp \omega _{eg}\right) ^{-1}\left\{ \cdots \right\} _{\mathbf {k}} \end{aligned}$$

For Herzberg–Teller interaction the Lamb shift in $\mathscr {L}_{\mathrm { el-vib-rad}}^{\left( \alpha ,\alpha ^{\prime }\right) }$ contains more terms

132 $$\begin{aligned} \Lambda _{\mathrm {el-vib-rad}}^{\left( *\right) }=\Lambda _{\mathrm { el-vib-rad}}^{\left( *\right) \left( +\right) }+\Lambda _{\mathrm { el-vib-rad}}^{\left( *\right) \left( -\right) }+\Lambda _{\mathrm { el-vib-rad}}^{\left( *\right) \left( +\right) \left( cr\right) }+\Lambda _{\mathrm {el-vib-rad}}^{\left( *\right) \left( -\right) \left( cr\right) } \end{aligned}$$

where

133 $$\begin{aligned} \Lambda _{\mathrm {el-vib-rad}}^{\left( *\right) \left( +\right) }&= {} i\sum _{i,i^{\prime }}\left\{ \Delta \tilde{\Im }_{\left( +,-\right) }^{\left( *\right) \left( +\right) }\left[ e^{i\Delta \tilde{v} _{i,i^{\prime }}^{\left( +\right) \left( \alpha ,\alpha ^{\prime }\right) }t}\left( C_{\alpha i}^{\dagger }\tilde{\rho }_{S}\left( t\right) B_{\alpha ^{\prime }i^{\prime }}^{\dagger }-\tilde{\rho }_{S}\left( t\right) B_{\alpha ^{\prime }i^{\prime }}^{\dagger }C_{\alpha i}^{\dagger }\right) \right. \right. \nonumber \\&\left. \quad +\, e^{-i\Delta \tilde{v} _{i,i^{\prime }}^{\left( -\right) \left( \alpha ,\alpha ^{\prime }\right) }t}\left( B_{\alpha i}\tilde{\rho }_{S}\left( t\right) B_{\alpha ^{\prime }i^{\prime }}^{\dagger }-\tilde{\rho }_{S}\left( t\right) B_{\alpha ^{\prime }i^{\prime }}^{\dagger }B_{\alpha i}\right) \right] \nonumber \\&\quad +\, \Delta \tilde{\Im }_{\left( -,+\right) }^{\left( *\right) \left( +\right) }\left[ e^{i\Delta \tilde{v}_{i,i^{\prime }}^{\left( -\right) \left( \alpha ,\alpha ^{\prime }\right) }t}\left( B_{\alpha i}^{\dagger }\tilde{\rho }_{S}\left( t\right) B_{\alpha ^{\prime }i^{\prime }}-\tilde{\rho }_{S}\left( t\right) B_{\alpha ^{\prime }i^{\prime }}B_{\alpha ^{\prime }i^{\prime }}^{\dagger }\right) \right. \nonumber \\&\left. \left. \quad +\, e^{-i\Delta \tilde{v}_{i,i^{\prime }}^{\left( +\right) \left( \alpha ,\alpha ^{\prime }\right) }t}\left( C_{\alpha i}\tilde{\rho }_{S}\left( t\right) B_{\alpha ^{\prime }i^{\prime }}-\tilde{\rho }_{S}\left( t\right) B_{\alpha ^{\prime }i^{\prime }}C_{\alpha i}\right) \right] \right\} +H.c. \end{aligned}$$

with

134 $$\begin{aligned}&\Delta \tilde{\Im }_{\left( \pm ,\mp \right) }^{\left( *\right) \left( u\right) }=\tilde{\Im }_{\left\{ n+1\right\} \pm }^{\left( *\right) \left( u\right) }-\tilde{\Im }_{\left\{ n\right\} \mp }^{\left( *\right) \left( u\right) } \end{aligned}$$

135 $$\begin{aligned}&\tilde{\Im }_{\left\{ \cdots \right\} \pm }^{\left( *\right) \left( u\right) }=\tilde{\Im }_{\left\{ \cdots \right\} }^{\left( *\right) }\left( \pm \Delta \tilde{v}_{eg,i^{\prime }}^{\left( u\right) \left( \alpha ^{\prime }\right) }\right) =\int d^{3}k~D\left( \mathbf {k}\right) \xi _{ \mathbf {k},i}^{\left( eg\right) \left( \alpha \right) }\xi _{\mathbf {k} ,i^{\prime }}^{\left( eg\right) \left( \alpha ^{\prime }\right) }P\left( \omega _{k}\mp \tilde{v}_{eg,i^{\prime }}^{\left( u\right) \left( \alpha ^{\prime }\right) }\right) ^{-1}\left\{ \cdots \right\} _{\mathbf {k}} \end{aligned}$$

A note for clarification, the superscript $u=\left\{ +,-\right\}$ follows from $\tilde{v}_{eg,i^{\prime }}^{\left( \pm \right) \left( \alpha ^{\prime }\right) }=\omega _{eg}\pm \tilde{v}_{i^{\prime }}^{\left( \alpha ^{\prime }\right) }$ and it tells us if the electronic frequency and vibrational frequency are being sum over or subtract; while the subscript ± tells us the sign in the Cauchy Principal value.

Similarly for the minus term

136 $$\begin{aligned} \Lambda _{\mathrm {el-vib-rad}}^{\left( *\right) \left( -\right) }&= {} i\left\{ \Delta \tilde{\Im }_{\left( -,+\right) }^{\left( *\right) \left( -\right) }\left[ e^{i\Delta \tilde{v}_{i,i^{\prime }}^{\left( +\right) \left( \alpha ,\alpha ^{\prime }\right) }t}\left( B_{\alpha i}^{\dagger }\tilde{\rho }_{S}\left( t\right) C_{\alpha ^{\prime }i^{\prime }}^{\dagger }-\tilde{\rho }_{S}\left( t\right) C_{\alpha ^{\prime }i^{\prime }}^{\dagger }B_{\alpha i}^{\dagger }\right) \right. \right. \nonumber \\&\left. \quad +\, e^{-i\Delta \tilde{v} _{i,i^{\prime }}^{\left( -\right) \left( \alpha ,\alpha ^{\prime }\right) }t}\left( C_{\alpha i}\tilde{\rho }_{S}\left( t\right) C_{\alpha ^{\prime }i^{\prime }}^{\dagger }-\tilde{\rho }_{S}\left( t\right) C_{\alpha ^{\prime }i^{\prime }}^{\dagger }C_{\alpha i}\right) \right] \nonumber \\&\quad +\, \Delta \tilde{\Im }_{\left( +,-\right) }^{\left( *\right) \left( -\right) }\left[ e^{i\Delta \tilde{v}_{i,i^{\prime }}^{\left( -\right) \left( \alpha ,\alpha ^{\prime }\right) }t}\left( C_{\alpha i}^{\dagger }\tilde{\rho }_{S}\left( t\right) C_{\alpha ^{\prime }i^{\prime }}-\tilde{\rho }_{S}\left( t\right) C_{\alpha ^{\prime }i^{\prime }}C_{\alpha i}^{\dagger }\right) \right. \nonumber \\&\left. \left. \quad +\, e^{-i\Delta \tilde{v}_{i,i^{\prime }}^{\left( +\right) \left( \alpha ,\alpha ^{\prime }\right) }t}\left( B_{\alpha i}\tilde{\rho }_{S}\left( t\right) C_{\alpha ^{\prime }i^{\prime }}-\tilde{\rho }_{S}\left( t\right) C_{\alpha ^{\prime }i^{\prime }}B_{\alpha i}\right) \right] \right\} +H.c. \end{aligned}$$

and the counter-rotating terms

137 $$\begin{aligned} \Lambda _{\mathrm {el-vib-rad}}^{\left( *\right) \left( +\right) \left( cr\right) }&= {} i\left\{ \Delta \tilde{\Im }_{\left( +,-\right) }^{\left( *\right) \left( +\right) }\left[ e^{i\left( \Delta \tilde{v}_{i,i^{\prime }}^{\left( +\right) \left( \alpha ,\alpha ^{\prime }\right) }+2\omega _{eg}\right) t}B_{\alpha i}^{\dagger }\tilde{\rho }_{S}\left( t\right) B_{\alpha ^{\prime }i^{\prime }}^{\dagger }+e^{-i\left( \Delta \tilde{v} _{i,i^{\prime }}^{\left( -\right) \left( \alpha ,\alpha ^{\prime }\right) }-2\omega _{eg}\right) t}C_{\alpha i}\tilde{\rho }_{S}\left( t\right) B_{\alpha ^{\prime }i^{\prime }}^{\dagger }\right] \right. \nonumber \\&\left. \quad +\, \Delta \tilde{\Im }_{\left( -,+\right) }^{\left( *\right) \left( +\right) }\left[ e^{i\left( \Delta \tilde{v}_{i,i^{\prime }}^{\left( -\right) \left( \alpha ,\alpha ^{\prime }\right) }-2\omega _{eg}\right) t}C_{\alpha i}^{\dagger }\tilde{\rho }_{S}\left( t\right) B_{\alpha ^{\prime }i^{\prime }}+e^{-i\left( \Delta \tilde{v}_{i,i^{\prime }}^{\left( +\right) \left( \alpha ,\alpha ^{\prime }\right) }+2\omega _{eg}\right) t}B_{\alpha i}\tilde{\rho }_{S}\left( t\right) B_{\alpha ^{\prime }i^{\prime }}\right] \right\} +H.c. \end{aligned}$$

and

138 $$\begin{aligned} \Lambda _{\mathrm {el-vib-rad}}^{\left( *\right) \left( -\right) \left( cr\right) }&= {} i\left\{ \Delta \tilde{\Im }_{\left( -,+\right) }^{\left( *\right) \left( -\right) }\left[ e^{i\left( \Delta \tilde{v}_{i,i^{\prime }}^{\left( +\right) \left( \alpha ,\alpha ^{\prime }\right) }-2\omega _{eg}\right) t}C_{\alpha i}^{\dagger }\tilde{\rho }_{S}\left( t\right) C_{\alpha ^{\prime }i^{\prime }}^{\dagger }+e^{-i\left( \Delta \tilde{v} _{i,i^{\prime }}^{\left( -\right) \left( \alpha ,\alpha ^{\prime }\right) }+2\omega _{eg}\right) t}B_{\alpha i}\tilde{\rho }_{S}\left( t\right) C_{\alpha ^{\prime }i^{\prime }}^{\dagger }\right] \right. \nonumber \\&\left. \quad +\, \Delta \tilde{\Im }_{\left( +,-\right) }^{\left( *\right) \left( -\right) }\left[ e^{-i\left( \Delta \tilde{v} _{i,i^{\prime }}^{\left( +\right) \left( \alpha ,\alpha ^{\prime }\right) }-2\omega _{eg}\right) t}C_{\alpha i}\tilde{\rho }_{S}\left( t\right) C_{\alpha ^{\prime }i^{\prime }}+e^{i\left( \Delta \tilde{v}_{i,i^{\prime }}^{\left( -\right) \left( \alpha ,\alpha ^{\prime }\right) }+2\omega _{eg}\right) t}B_{\alpha i}^{\dagger }\tilde{\rho }_{S}\left( t\right) C_{\alpha ^{\prime }i^{\prime }}\right] \right\} +H.c. \end{aligned}$$

The number of terms in cr are half the number of terms in RWA due to vanishing of the terms with $\sigma \sigma$ and $\sigma ^{\dagger }\sigma ^{\dagger }$.

The Lamb shift for cross term $\mathscr {L}_{\mathrm {(el\times el-vib)-rad} }^{\left( \alpha \right) }$

139 $$\begin{aligned} \Lambda _{\mathrm {(el\times el-vib)-rad}}^{\left( \alpha ,i^{\prime }\right) }&= {} i\left\{ \Delta \hat{\Im }_{\left( +,-\right) }^{\left( \alpha ,i^{\prime }\right) \left( +\right) }\left[ e^{i\tilde{v}_{i^{\prime }}^{\left( \alpha \right) }t}\left( \sigma \tilde{\rho }_{S}\left( t\right) B_{\alpha i^{\prime }}^{\dagger }-\tilde{\rho }_{S}\left( t\right) B_{\alpha i^{\prime }}^{\dagger }\sigma \right) +e^{i\left( \tilde{v}_{i^{\prime }}^{\left( \alpha \right) }+2\omega _{eg}\right) t}\sigma ^{\dagger }\tilde{ \rho }_{S}\left( t\right) B_{\alpha i^{\prime }}^{\dagger }\right] \right. \nonumber \\&\left. \quad +\, \Delta \hat{\Im }_{\left( -,+\right) }^{\left( \alpha ,i^{\prime }\right) \left( +\right) }\left[ e^{-i\tilde{v}_{i^{\prime }}^{\left( \alpha \right) }t}\left( \sigma ^{\dagger }\tilde{\rho }_{S}\left( t\right) B_{\alpha i^{\prime }}-\tilde{\rho }_{S}\left( t\right) B_{\alpha i^{\prime }}\sigma ^{\dagger }\right) +e^{-i\left( \tilde{v}_{i^{\prime }}^{\left( \alpha \right) }+2\omega _{eg}\right) t}\sigma \tilde{\rho }_{S}\left( t\right) B_{\alpha i^{\prime }}\right] \right\} \nonumber \\&\quad \times \, i\left\{ \Delta \hat{\Im }_{\left( -,+\right) }^{\left( \alpha ,i^{\prime }\right) \left( -\right) }\left[ e^{i\tilde{v}_{i^{\prime }}^{\left( \alpha \right) }t}\left( \sigma ^{\dagger }\tilde{\rho }_{S}\left( t\right) C_{\alpha i^{\prime }}^{\dagger }-\tilde{\rho }_{S}\left( t\right) C_{\alpha i^{\prime }}^{\dagger }\sigma ^{\dagger }\right) +e^{i\left( \tilde{v} _{i^{\prime }}^{\left( \alpha \right) }-2\omega _{eg}\right) t}\sigma \tilde{ \rho }_{S}\left( t\right) C_{\alpha i^{\prime }}^{\dagger }\right] \right. \nonumber \\&\left. \quad +\, \Delta \hat{\Im }_{\left( +,-\right) }^{\left( \alpha ,i^{\prime }\right) \left( -\right) }\left[ e^{-i\tilde{v}_{i^{\prime }}^{\left( \alpha \right) }t}\left( \sigma \tilde{\rho }_{S}\left( t\right) C_{\alpha i^{\prime }}-\tilde{\rho }_{S}\left( t\right) C_{\alpha i^{\prime }}\sigma \right) +e^{-i\left( \tilde{v}_{i^{\prime }}^{\left( \alpha \right) }-2\omega _{eg}\right) t}\sigma ^{\dagger }\tilde{\rho }_{S}\left( t\right) C_{\alpha i^{\prime }}\right] \right\} +H.c. \end{aligned}$$

where we have defined with

140 $$\begin{aligned}&\Delta \hat{\Im }_{\left( \pm ,\mp \right) }^{\left( \alpha ,i^{\prime }\right) \left( u\right) }=\hat{\Im }_{\left\{ n+1\right\} \pm }^{\left( \alpha ,i^{\prime }\right) \left( u\right) }-\hat{\Im }_{\left\{ n\right\} \mp }^{\left( \alpha ,i^{\prime }\right) \left( u\right) } \end{aligned}$$

141 $$\begin{aligned}&\hat{\Im }_{\left\{ \cdots \right\} \pm }^{\left( \alpha ,i^{\prime }\right) (u)}=\hat{\Im }_{\left\{ \cdots \right\} }^{\left( \alpha ,i^{\prime }\right) }\left( \pm \tilde{v}_{eg,i^{\prime }}^{\left( u\right) \left( \alpha \right) }\right) =\int d^{3}k~D\left( \mathbf {k}\right) \xi _{\mathbf {k} ,i^{\prime }}^{\left( eg\right) \left( \alpha \right) }\sum _{i}\varsigma _{i, \mathbf {k}}^{\left( ge\right) }P\left( \omega _{k}\mp \tilde{v} _{eg,i^{\prime }}^{\left( u\right) \left( \alpha \right) }\right) ^{-1}\left\{ \cdots \right\} _{\mathbf {k}} \end{aligned}$$

with $$u=\left\{ +,-\right\} .$$

The Lamb shift for the reversed cross term in $\mathscr {L}_{\mathrm { (el-vib\times el)-rad}}^{\left( \alpha \right) }$ follows similarly

142 $$\begin{aligned} \Lambda _{\mathrm {(el-vib\times el)-rad}}^{\left( \alpha ,i\right) }&= {} i\left\{ \Delta \check{\Im }_{\left( +,-\right) }^{\left( \alpha ,i\right) \left( +\right) }e^{i\tilde{v}_{i}^{\left( \alpha \right) }t}\left( C_{\alpha i}^{\dagger }\tilde{\rho }_{S}\left( t\right) \sigma ^{\dagger }- \tilde{\rho }_{S}\left( t\right) \sigma ^{\dagger }C_{\alpha i}^{\dagger }\right) \right. \nonumber \\&\left. \quad +\, \Delta \check{\Im }_{\left( -,+\right) }^{\left( \alpha ,i\right) \left( +\right) }e^{-i\tilde{v}_{i}^{\left( \alpha \right) }t}\left( C_{\alpha i}\tilde{\rho }_{S}\left( t\right) \sigma -\tilde{\rho }_{S}\left( t\right) \sigma C_{\alpha i}\right) \right\} \nonumber \\&\quad \times \, i\left\{ \Delta \check{\Im }_{\left( -,+\right) }^{\left( \alpha ,i\right) \left( -\right) }e^{-i\tilde{v}_{i}^{\left( \alpha \right) }t}\left( B_{\alpha i}\tilde{\rho }_{S}\left( t\right) \sigma ^{\dagger }-\tilde{\rho } _{S}\left( t\right) \sigma ^{\dagger }B_{\alpha i}\right) \right. \nonumber \\&\left. \quad +\, \Delta \check{\Im }_{\left( +,-\right) }^{\left( \alpha ,i\right) \left( -\right) }e^{i\tilde{v}_{i}^{\left( \alpha \right) }t}\left( B_{\alpha i}^{\dagger }\tilde{\rho }_{S}\left( t\right) \sigma -\tilde{\rho } _{S}\left( t\right) \sigma B_{\alpha i}^{\dagger }\right) \right\} +H.c. \end{aligned}$$

where we have defined

143 $$\begin{aligned}&\check{\Im }_{\left( \pm ,\mp \right) }^{\left( \alpha ,i\right) \left( u\right) }=\check{\Im }_{\left\{ n+1\right\} \pm }^{\left( \alpha ,i\right) \left( u\right) }-\check{\Im }_{\left\{ n\right\} \mp }^{\left( \alpha ,i\right) \left( u\right) } \end{aligned}$$

144 $$\begin{aligned}&\check{\Im }_{\left\{ \cdots \right\} \pm }^{\left( \alpha ,i\right) (u)}= \check{\Im }_{\left\{ \cdots \right\} }^{\left( \alpha ,i\right) }\left( \pm \tilde{v}_{eg,i}^{\left( u\right) \left( \alpha \right) }\right) =\int d^{3}k~D\left( \mathbf {k}\right) \xi _{\mathbf {k},i}^{\left( eg\right) \left( \alpha \right) }\sum _{i^{\prime }}\varsigma _{i^{\prime },\mathbf {k} }^{\left( ge\right) }P\left( \omega _{k}\mp \tilde{v}_{eg,i}^{\left( u\right) \left( \alpha \right) }\right) ^{-1}\left\{ \cdots \right\} _{ \mathbf {k}}. \end{aligned}$$
